# Template-Confined Synthesis of 1 nm High-Entropy-Alloy Nanoparticle Library for Electrocatalysis

**DOI:** 10.1021/acsnano.6c06368

**Published:** 2026-06-24

**Authors:** Chun-Wei Chang, Yueh-Chun Hsiao, Jui-Tai Lin, Zong-Ying He, Cheng-Kuang Lin, Yi Chen, Wen-Yang Huang, Wei-Hsiang Huang, Li-Yu Wang, Han-Yuan Liu, Yun-Shan Tsai, Chia-Che Chang, Wei-Chih Hsiao, Wen-Jing Zeng, Ying-Rui Lu, Kun-Han Lin, Sung-Fu Hung, Chih-Wen Pao, Chia-Min Yang, Alexander J. Cowan, Tung-Han Yang

**Affiliations:** † Department of Chemical Engineering, 34881 National Tsing Hua University, Hsinchu 300044, Taiwan; ‡ Stephenson Institute for Renewable Energy and Department of Chemistry, 4591 University of Liverpool, Liverpool L69 7ZF United Kingdom; § Department of Applied Chemistry and Center for Emergent Functional Matter Science, 34914 National Yang Ming Chiao Tung University, Hsinchu 300093, Taiwan; ∥ 57815 National Synchrotron Radiation Research Center, Hsinchu 300092, Taiwan; ⊥ Department of Chemistry, 34881 National Tsing Hua University, Hsinchu 300044, Taiwan; # College of Semiconductor Research, 34881 National Tsing Hua University, Hsinchu 300044, Taiwan; ∇ High Entropy Materials Center, National Tsing Hua University, Hsinchu 300044, Taiwan

**Keywords:** high-entropy-alloy nanoparticles, size control, autocatalytic reduction, *op*erando synchrotron X-ray absorption spectroscopy, electrocatalysis

## Abstract

High-entropy-alloy (HEA) nanoparticles containing at least five elements are an emerging family of catalysts. Due to the increased complexity of multicomponent atomic mixing, the size reduction of HEA nanoparticles down to around 1 nm is a substantial challenge, which is crucial to enhance the efficiency of atom utilization. Herein, we report a template-confined synthesis strategy for constructing a library of ultrasmall HEA nanoparticles with sizes of approximately 1 nm and compositions containing up to ten elements, including Pt, Pd, Ru, Rh, Fe, Co, Ni, Cu, Mo, and Zn. By leveraging the confinement effect of the mesoporous carbon structure and the autocatalytic reduction of the metal-precursor mixture at a low reaction temperature, we successfully achieve the uniform embedding of a diverse array of ultrasmall multicomponent HEA nanoparticles within the mesoporous framework. Additionally, we demonstrate that the 1 nm PtRuFeCoNi nanoparticles exhibit enhanced mass activity and superior atom utilization in both hydrogen evolution and hydrogen oxidation reactions, surpassing other quinary HEA and commercial Pt/C catalysts. Furthermore, *operando* X-ray absorption spectroscopy and theoretical calculations suggest a cooperative multielement effect among the five constituent elements, in which Pt- and Ru-containing local environments provide favorable sites for H-intermediate adsorption and contribute to the enhanced catalytic performance.

## Graphical abstract

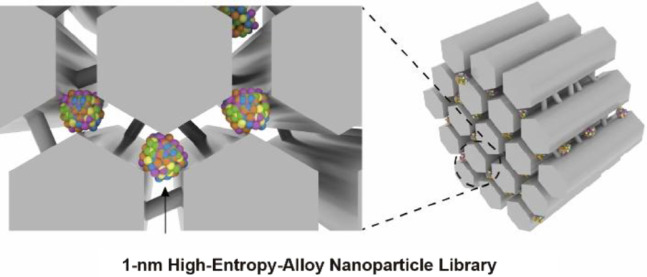

## Introduction

High-entropy-alloy (HEA) nanoparticles, which consist of five or more metallic elements in a single phase, have attracted considerable interest in electrocatalysis because of their unique properties.^1–32^ virtually limitless compositional variations, abundant active sites, and synergistic interactions at the atomic scale. For example, recent theoretical calculations have substantiated a robust synergistic effect arising from the multielement composition of PtFeCoNiCu HEA nanoparticles.^14^ This effect holds promise in optimizing the hydrogen adsorption-free energy, ΔG_H*_, which serves as a widely utilized descriptor for the ability of the hydrogen evolution reaction (HER). It achieves an ideal ΔG_H*_ value by judiciously selecting combinations of metals with varying degrees of hydrogen adsorption energies, in accordance with the Sabatier principle. Similarly, theoretical studies on PtRuNiCoFeMo nanowires have shown that synergistic interactions among different metal sites can significantly modulate the binding of protons and hydroxyl species, thereby enhancing hydrogen oxidation reaction (HOR) activity.^15^ HOR is a key anodic reaction in anion-exchange membrane fuel cells (AEMFCs), which have attracted increasing attention owing to their ability to directly convert chemical energy into electricity under alkaline conditions.^33,34^ In alkaline media, HOR typically proceeds via the Tafel–Volmer or Heyrovsky–Volmer pathways, involving adsorbed hydrogen and hydroxyl species.^35,36^ Although the elementary steps are well established, the dominant reaction pathway and rate-determining step remain under debate, as HOR kinetics are strongly influenced by hydrogen binding strength, hydroxide interaction, and interfacial water structure. Consequently, catalysts that can simultaneously optimize hydrogen adsorption and local surface coordination environments are highly desirable for enhancing HOR kinetics in alkaline electrolytes. To date, two primary synthetic approaches including thermal annealing treatments^12,14,16–18,21–25^ (e.g., carbothermal shock, fast-moving bed pyrolysis, and hydrogen spillover-driven synthesis) and wet-chemical synthesis^15,19,20,26–32^ (e.g., solvothermal and polyol synthesis) have been demonstrated for the synthesis of diverse HEA nanoparticles with the mixing of multiple chemical elements. However, their particle sizes typically exceed 5 nm. In catalytic applications, an ideal size range for HEA nanoparticles is proposed to be between 1 and 5 nm. Reducing particle size below 5 nm leads to a substantial increase in atom utilization efficiency, particularly in terms of the proportion of atoms occupying the surface. As an illustrative example, consider the significant increase in utilization efficiency from 35% to 76% when the size of a Pt nanoparticle is reduced from 7.0 to 1.2 nm. This observation justifies the widespread use of Pt nanoparticles with diameters around 3–5 nm as commercial catalysts for various catalytic processes.

The challenge of achieving precise size control down to approximately 1 nm while maintaining a high-entropy mixing state is significant, mainly due to the increased complexity of multicomponent atomic mixing in HEA nanoparticles. Typically, thermal annealing treatments are utilized at temperatures exceeding 500 °C to facilitate high-entropy mixing by accelerating the diffusion of metal atoms.^12,14,16–18,21–25^ However, these processes often trigger substantial sintering, leading to the formation of nonuniformly sized HEA nanoparticles, typically larger than 5 nm, especially on thermally resistant substrates like carbon and Al_2_O_3_. In contrast, several research groups have successfully reduced the size of HEA nanoparticles and nanostructures to below 5 nm using wet-chemical synthesis under mild conditions.^15,29,31,32^ For example, ultrasmall PdPtRhIrRu HEA nanoparticles with an average size of 1.32 ± 0.41 nm have been successfully synthesized through a colloidal approach using continuous-flow liquid-phase reduction.^29^ In addition to nearly spherical HEA nanoparticles, one-dimensional (1D) HEA nanowires or nanotubes with sub-1 nm wall thicknesses have recently been engineered to maximize atom efficiency.^15,31,32^ However, liquid-phase synthesis typically involves colloidal stabilizers, such as organic macromolecules and polymers, as well as organic solvents and reagents, which can help prevent nanoparticle aggregation and control particle growth. Despite repeated washing after synthesis, these organic species often remain chemisorbed on the surface of HEA nanoparticles. This presence can potentially impact the performance of HEA nanoparticles in heterogeneous catalysis. Therefore, it is crucial to develop a general yet robust method for preparing ultrasmall HEA nanoparticles with a narrow size distribution and a clean surface.

Here, we successfully synthesized a family of ultrasmall (∼1 nm) HEA nanoparticles free of colloidal stabilizer embedded uniformly in plate-like nitrogen-doped ordered mesoporous carbon (N-doped CMK-3). By combining a pore-volume-matched dry impregnation strategy with the confinement effect of the ordered mesoporous structure and an autocatalytic formation mechanism, we achieved the synthesis of more than 10 types of homogeneous 1 nm HEA nanoparticles. In contrast to conventional impregnation followed by thermal reduction, which typically leads to severe particle growth when multiple metals are involved, our strategy enables simultaneous size confinement and atomic-level mixing at the ultrasmall size regime (Table S1). This capability is crucial for maintaining a high-entropy mixing state at around 1 nm, where multicomponent diffusion and phase separation are otherwise difficult to suppress. These nanoparticles can incorporate up to 10 different elements, including Pt, Pd, Ru, Rh, Fe, Co, Ni, Cu, Mo, and Zn, as these elements are commonly found in catalysts used across a wide range of catalytic reactions. Notably, this synthesis occurs at a very low temperature, highlighting the efficacy of this strategy in achieving HEA nanoparticles with diverse combinations of metals at an extremely small size. Utilizing this nanoparticle family, we can obtain a systematic understanding of the electrocatalytic activities of diverse HEA nanoparticles toward HER and HOR due to their uniform size of approximately 1 nm. We demonstrate that the 1 nm PtRuFeCoNi nanoparticles exhibit high mass activity and atom utilization in both HER and HOR, surpassing other quinary HEA and commercial Pt/C catalysts. Moreover, to decouple intrinsic size effects from compositional stability and to validate the generality of our synthesis strategy, a noble-metal-only HEA system (PdPtRhIrRu) was synthesized under identical conditions as a control catalyst. This comparison allows us to distinguish size- and coordination-driven catalytic effects from the potential influence of 3d metal dissolution, thereby providing deeper insight into structure-performance relationships in ultrasmall HEA catalysts. Importantly, electronic interactions among the five mixed elements create Pt- and Ru-containing local environments that are favorable for H-intermediate adsorption, thereby contributing to the enhanced catalytic performance of PtRuFeCoNi nanoparticles. This synergistic effect is confirmed through *operando* synchrotron X-ray absorption spectroscopy (XAS) and density functional theory (DFT) simulations.

## Results and Discussion

### Template-Confined Synthesis of 1 nm HEA Nanoparticles

In this study, we prepared ultrasmall HEA nanoparticles within plate-like ordered mesoporous N-doped CMK-3, as illustrated in Figure 1a. The porous structure of N-doped CMK-3 can be utilized to immobilize and disperse HEA nanoparticles, as well as to control their size. Figure 1b and Table S2 summarize the nanoparticle diameters of HEA nanoparticles obtained by thermal annealing treatments,^12,14,16–25^ wet chemical synthesis,^15,19,20,26–32^ laser ablation,^9,37^ aerosol synthesis,^38^ and template-confined synthesis obtained in this work. We achieved 1.12 nm ultrasmall HEA nanoparticles free of colloidal stabilizer via template-confined synthesis. Using the synthesis of PtRuFeCoNi nanoparticles as an example, the protocol involves two key steps: preparation of the N-doped CMK-3 by the modified impregnation method and formation of PtRuFeCoNi nanoparticles embedded in the N-doped CMK-3 through the chemical reduction method under the reducing environment (see the Experimental Section for details). Note that the metal precursors for Pt, Ru, Fe, Co, and Ni elements exhibit significant differences (Table S3), potentially leading to varying reduction rates. This discrepancy poses a challenge to the formation of PtRuFeCoNi nanoparticles.

**Figure 1:**
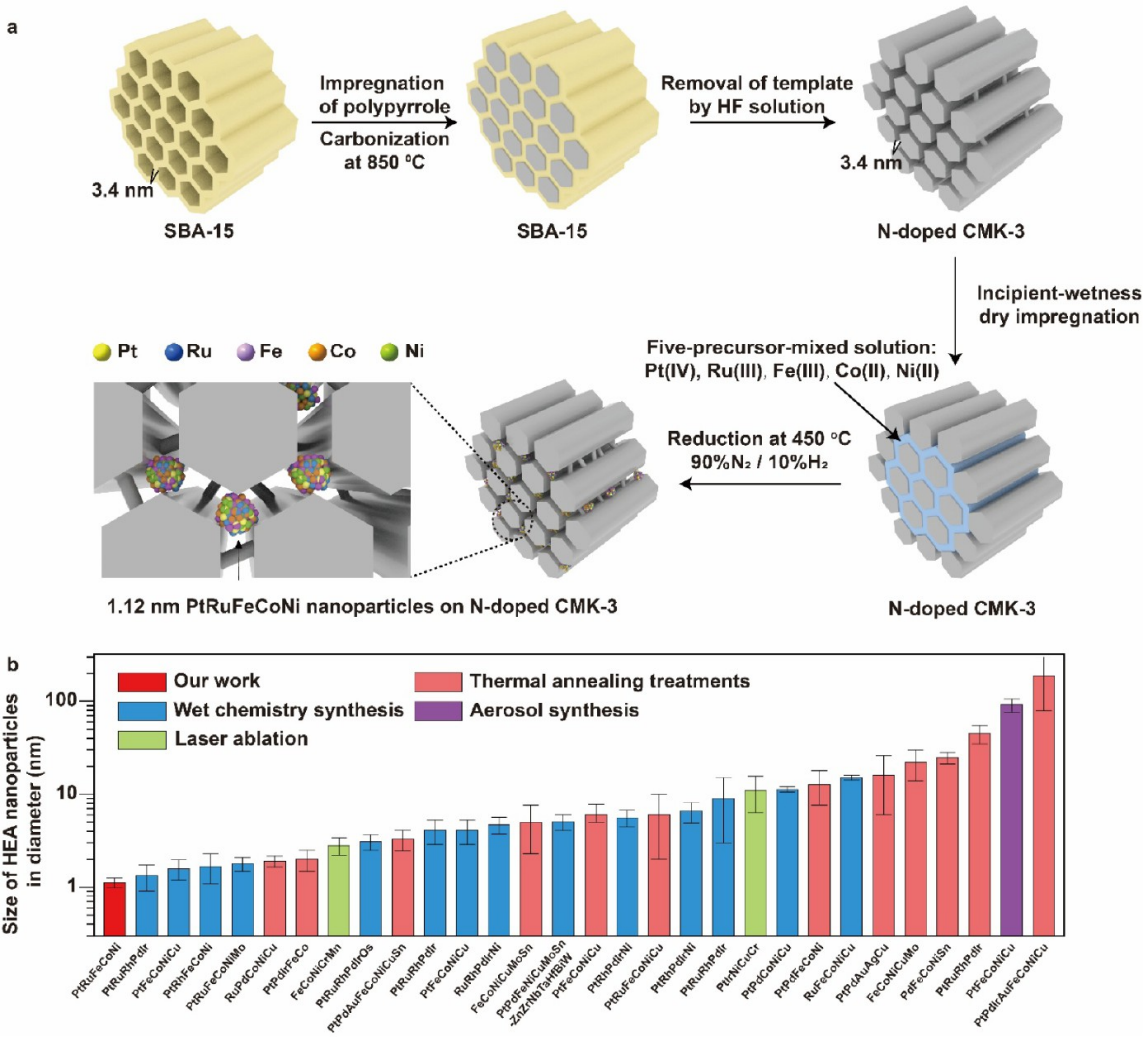
Template-confined synthesis of HEA nanoparticles with an average diameter of ∼1 nm. (a) Schematic diagram for size control of PtRuFeCoNi HEA nanoparticles using mesoporous N-doped CMK-3 as a template. (b) Summary of size distributions (in diameter) of HEA nanoparticles prepared by various bottom-up methods.

In the first step, the polypyrrole as the precursors of both carbon and nitrogen was first infiltrated into a presynthesized Santa Barbara Amorphous-15 (SBA-15) silica platelets, followed by carbonization at 850 °C for 4 h. After that, the carbon-silica composites obtained were dissolved by diluted HF solution to remove the silica template for the formation of a nitrogen-rich mesoporous framework rigidly interconnected by smaller carbon rods (i.e., N-doped CMK-3). Figure S1 shows the characterizations of SBA-15, the small-angle X-ray diffraction (XRD) patterns confirmed a 2D hexagonal MCM-41 type lattice structure, with observed 1 0, 1 1, and 2 0 reflection peaks. Scanning electron microscopy (SEM) images revealed a plate thickness of ∼170 nm with uniform morphology and size distribution. High-resolution transmission electron microscopy (HR-TEM) images along the pore axis of SBA-15 reveal that the bright areas correspond to the cylindrical pores, while the dark regions represent the silica walls. Furthermore, the SEM, HR-TEM, and XRD show that the N-doped CMK-3 exhibits a highly ordered 2D hexagonal (*p6 mm* space group) mesoporous structure, with a carbon rod spacing of 3.55 nm, as shown in Figures 2a–c and S2. The N_2_ adsorption–desorption isotherm reveals that N-doped CMK-3 holds a uniform mesopore diameter of 3.4 nm, a large specific surface area of 1036 m^2^ g^–1^, and a total pore volume of 1.253 cm^3^ g^–1^ (Figure 2d and e). X-ray photoelectron spectroscopy (XPS) confirms that N-doped CMK-3 contains a relatively high nitrogen content (2.08 at %), predominantly as graphitic nitrogen (Figure S3 and Table S4). This graphitic nitrogen enhances metal–support interactions, facilitates interfacial electron transfer, and helps suppress migration during high-temperature treatment.^39^ In addition, we observed that the intensity ratio of D and G peaks (I_D_/I_G_) of the CMK-3 after the nitrogen doping increased in the Raman spectra (Figure S4), which can be attributed to the doping of nitrogen and thus the increase of defects in the graphene structure. In the second step, the five-precursor-mixed ethanol solution including Pt­(IV), Ru­(III), Fe­(III), Co­(II), and Ni­(II) metal chlorides was introduced into the mesoporous N-doped CMK-3 through an improved incipient wetness method. This method facilitates precise control over both the quantity and uniform distribution of the mixed precursors within the material. Subsequently, PtRuFeCoNi nanoparticles embedded in the N-doped CMK-3 were obtained by the chemical reduction of the mixed precursors at 450 °C for 2 h in the forming gas (10% H_2/_90% N_2_) flow.

**Figure 2:**
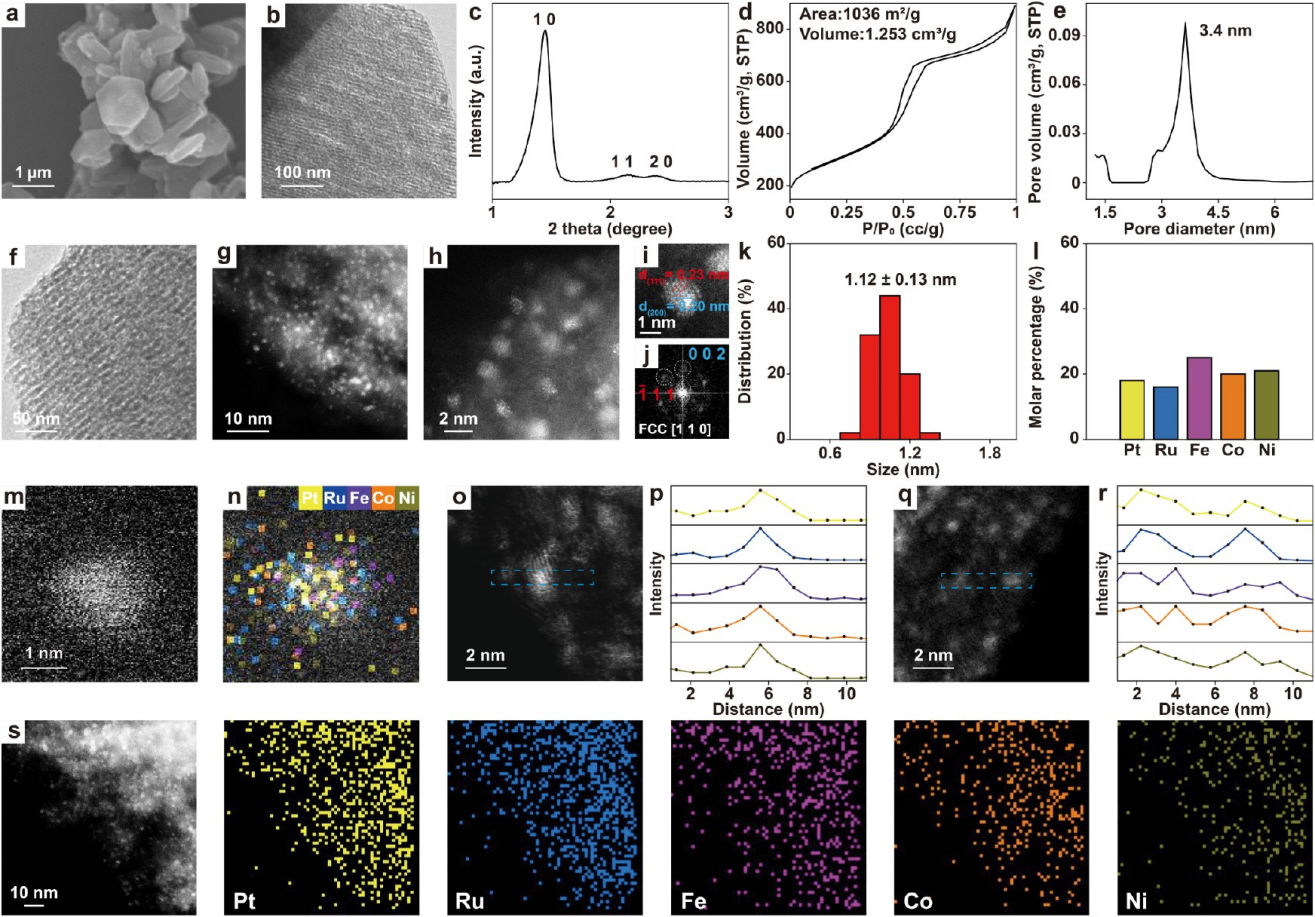
Material characterizations of N-doped CMK-3 and 1 nm PtRuFeCoNi HEA nanoparticles. (a) SEM, (b) TEM images, (c) small angle XRD pattern, (d) nitrogen adsorption–desorption isotherm, and (e) NLDFT pore size distribution of N-doped CMK-3. (f) TEM image, (g) low and (h, (i) high magnification HAADF-STEM images, (j) FFT analysis, (k) size distribution, (l) ICP-OES analysis. (m) HAADF-STEM image and the (n) corresponding EDS mapping of individual 1 nm PtRuFeCoNi HEA nanoparticle. The EDS line scans of (o, p) single and (q, r) paired 1 nm PtRuFeCoNi HEA nanoparticles. (s) Low-magnification EDS mappings of PtRuFeCoNi HEA nanoparticles supported on N-doped CMK-3 (note that the total metal loading amount is 3.49%).

Figure 2f and g show the low-magnification transmission electron microscopy (TEM) and high-angle annular dark-field scanning electron microscopy (HAADF-STEM) images of the resultant PtRuFeCoNi nanoparticles loaded on the N-doped CMK-3, respectively, indicating the uniform distribution of ultrasmall PtRuFeCoNi nanoparticles without aggregation. As shown in Figure 2h, the high-magnification HAADF-STEM image exhibits the low-crystalline structure of PtRuFeCoNi nanoparticles, which could be attributed to the ultrasmall size of nanoparticles and different atomic radii of five mixed elements. Figure 2i and j reveal the atomic-resolution HAADF-STEM image and the corresponding fast Fourier transform (FFT) pattern of a single PtRuFeCoNi nanoparticle. The observations suggest the presence of a face-centered cubic (FCC) structure. The measured lattice spacings of 0.20 and 0.23 nm correspond to the (002) and (111) crystallographic planes of the FCC lattice, respectively, corresponding to an estimated lattice constant of approximately 4.0 Å. This experimentally derived lattice parameter is larger than the theoretical value predicted by Vegard’s law based on the ICP-OES result (Table S5). Such lattice expansion is commonly observed in HEA nanoparticles and can be attributed to the severe atomic size mismatch among constituent elements, finite-size effects associated with ultrasmall nanoparticles, and/or partial surface oxidation of more oxophilic elements. As shown in Figure 2k, the PtRuFeCoNi nanoparticles exhibit an average diameter of 1.12 ± 0.13 nm, showcasing their ultrasmall size and narrow size distribution. Based on this average size, each nanoparticle is estimated to contain approximately 43 atoms. Notably, this represents the ultrasmall and highly size-confined among reported HEA nanoparticles (Figure 1b and Table S2), while maintaining a surface-to-volume ratio exceeding 70%. The total metal loading amount of PtRuFeCoNi nanoparticles embedded in the N-Doped CMK-3 is 3.49 wt % and atomic percentages of Pt, Ru, Fe, Co, and Ni elements are 17.8, 15.6, 24.9, 20.4, and 21.3 at %, respectively, which are determined by the inductively coupled plasma optical emission spectrometry (ICP-OES) (Figure 2l). The quantitative analysis is performed using well-established calibration curves with excellent linearity (Figure S5), and the characteristic emission lines of the constituent elements are well separated without spectral overlap (Figure S6), ensuring reliable and accurate elemental quantification. In addition, no distinct metal diffraction peaks are observed in the XRD patterns (Figures S7 and S8), which can be attributed to the combined effects of low metal loading, the carbon support background, severe size-induced peak broadening, and limited long-range crystallographic coherence within the approximately 1 nm confined nanoparticles. In general, such ultrasmall crystalline domains produce broadened and weakened diffraction features, making them difficult to distinguish from the background in XRD analysis. The configurational entropy of PtRuFeCoNi nanoparticles is calculated as 1.59R (where R is the gas constant), which meets the definition of HEA. We further analyzed the individual PtRuFeCoNi nanoparticle by EDS mappings, along with the corresponding spectrum, to examine the element distribution, as shown in Figure 2m, n, and S9. The homogeneous distributions of all five elements throughout the entire area of the nanoparticle were observed, indicating the formation of high-entropy mixing state. It is important to note that no significant metal signals were detected in the EDS spectrum for the region corresponding to the N-doped CMK-3 without PtRuFeCoNi nanoparticles (Figure S9). Additionally, the line scan analysis of both single and paired HEA nanoparticles (Figure 2p and r) shows distinct single and double peaks corresponding to the signals of the five elements, further confirming their homogeneous distributions within the nanoparticles. These results are also consistent with low-magnification EDS mappings (Figure 2s), supporting the formation of HEA nanoparticles. As a chemically robust control system, noble-metal-only PdPtRhIrRu HEA nanoparticles were synthesized under identical conditions. As shown in Figure S10, TEM and HAADF-STEM images reveal uniformly dispersed ultrasmall nanoparticles with an average diameter of 1.48 ± 0.33 nm. High-magnification HAADF-STEM imaging and FFT analysis of individual nanoparticles confirm an FCC structure, with lattice spacings of 0.23 and 0.20 nm corresponding to the (111) and (200) planes, respectively. ICP-OES analysis further confirms a near-equiatomic elemental composition of Pd, Pt, Rh, Ir, and Ru. In addition, EDS elemental mapping images demonstrate homogeneous and overlapping spatial distributions of all five noble metals throughout the nanoparticles, indicating the absence of elemental segregation and confirming the formation of chemically uniform PdPtRhIrRu HEA nanoparticles. This noble-metal HEA system thus serves as an ideal reference for decoupling size effects from compositional stability in subsequent electrocatalytic studies.

Importantly, even with an increased loading amount of 9.16 wt % (Figure 3) at the same temperature of 450 °C, Figure 3a and b show the HAADF-STEM of high-loading PtRuFeCoNi nanoparticles, which continue to exhibit a small and uniform distribution, measuring 1.21 ± 0.19 nm, as shown in Figure 3c. EDS mapping (Figure 3d) also shows the homogeneous distributions of five elements in high-loading PtRuFeCoNi nanoparticles. Additionally, to investigate the impact of thermal annealing treatments on the size of HEA nanoparticles, we performed the synthesis of quinary PtRuFeCoNi nanoparticles at varying temperatures (250, 650, and 850 °C) under the forming-gas atmosphere, as shown in Figure 3e–g. At the low temperature of 250 °C, the PtRuFeCoNi nanoparticles display a narrow size distribution of 1.02 ± 0.17 nm within the mesoporous carbon matrix (Figure 3h). As the temperature increases to 650 and 850 °C, the PtRuFeCoNi nanoparticles exhibit a slight increase in size to 1.81 ± 0.19 nm (Figure 3i) and 2.34 ± 0.20 nm (Figure 3j), respectively, while remaining uniformly dispersed within the pores of N-doped CMK-3. In contrast, when using conventional powdered carbon black (Vulcan XC-72R) as the support without the confinement effect, as shown in Figure 3k and l, the formation of larger HEA nanoparticles (3.96 ± 1.68 nm) irregularly distributed on the carbon surface was observed (Figure 3m) at 450 °C under the forming-gas atmosphere. Their EDS mappings were also provided, as shown in Figure 3n. These findings highlight the significant advantages of using mesoporous N-doped CMK-3 as a template, enabling the production of small-sized HEA nanoparticles with uniform distribution.

**Figure 3:**
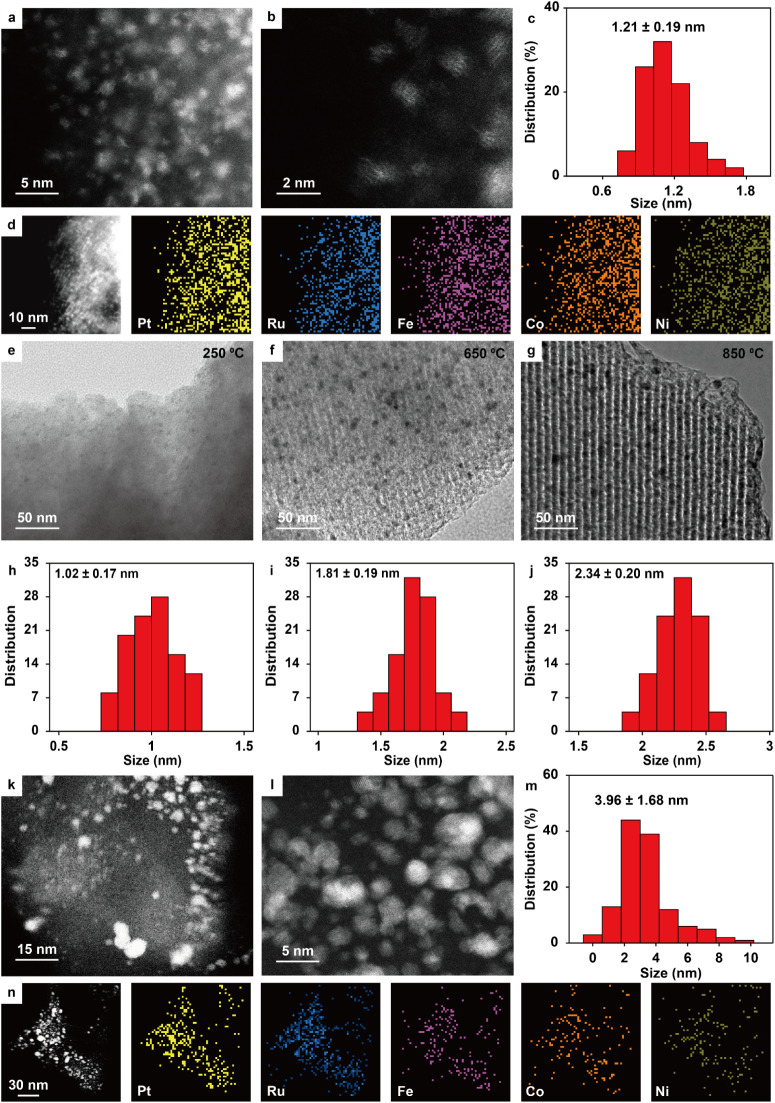
Effect of parameters on the size of PtRuFeCoNi HEA nanoparticles. Loading amount: (a) Low and (b) high magnification HAADF-STEM images, (c) size distribution, and (d) EDS mappings for 1 nm PtRuFeCoNi nanoparticles supported on N-doped CMK-3 with the total metal loading amount of 9.16 wt %. Temperature: (e to g) TEM images for PtRuFeCoNi supported on N-doped CMK-3 synthesized in 10% H_2/_90% N_2_ flow at (e) 250 °C, (f) 650 °C, and (g) 850 °C. (h to j) Size distributions of PtRuFeCoNi supported on N-doped CMK-3 synthesized in 10% H_2/_90% N_2_ flow at (h) 250 °C, (i) 650 °C, and (j) 850 °C. Carbon support: (k) Low and (l) high magnification HAADF-STEM images, (m) size distribution, (n) EDS mappings of PtRuFeCoNi nanoparticles supported on XC-72R with the total metal loading amount of 8.5 wt %.

The chemical states of the representative 1 nm PtRuFeCoNi nanoparticles embedded in the N-doped CMK-3 were analyzed by XPS, as shown in Figures 4a–f and S11. Given that the PtRuFeCoNi HEA nanoparticles are approximately 1 nm in diameter, comparable to or smaller than the typical inelastic mean free path of photoelectrons under Al Kα radiation (ca. 1–3 nm), XPS is expected to effectively probe nearly the entire nanoparticle. It is worth noting that the XPS signals of Fe, Co, and Ni appear relatively weaker compared to those of Pt and Ru, which can be primarily attributed to their substantially lower XPS sensitivity factors (SFs) (Table S6), rather than a lower elemental abundance. The XPS survey spectrum shows the presence of Pt, Ru, Fe, Co, Ni, C, O, and N elements (Figure 4a). In the high-resolution XPS spectra of Pt 4f, Pt exists mainly in the form of Pt^0^ and a small part of Pt^2+^, and the corresponding peak positions of Pt^0^ 4f_7/2_, Pt^0^ 4f_5/2_, Pt^2+^ 4f_7/2_, and Pt^2+^4f_5/2_ are at 71.7, 75.0, 73.0, and 76.3 eV, respectively, similar to bulk Pt (Figure 4b). The Ru 3p spectrum shows that Ru exists in the states of both Ru^0^ (Ru^0^ 3p_3/2_ at 462.8 eV and Ru^0^ 3p_3/2_ at 485.0 eV) and Ru^4+^ (Ru^4+^ 3p_3/2_ at 467.7 eV and Ru^4+^ 3p_3/2_ at 489.3 eV) (Figure 4c). For the Fe 2p spectrum, the main doublets at 706.8, 710.7, 712.7, 720.4, 723.7, and 725.8 eV correspond to Fe^0^ 2p_3/2_, Fe^2+^ 2p_3/2_, Fe^3+^ 2p_3/2_, Fe^0^ 2p_1/2_, Fe^2+^ 2p_1/2_, and Fe^3+^ 2p_1/2_, respectively (Figure 4d). For Co, the presence of Co^0^ and Co^2+^ species were observed, and the peaks at 778.6, 781.9, 793.6, and 796.9 eV are ascribed to Co^0^ 2p_3/2_, Co^2+^ 2p_3/2_, Co^0^ 2p_1/2_, Co^2+^ 2p_1/2_, respectively (Figure 4e). The Ni 2p spectrum suggests the coexistence of Ni^0^ 2p_3/2_ (854.0 eV), Ni^2+^ 2p_3/2_ (856.4 eV), Ni^0^ 2p_1/2_ (871.4 eV), and Ni^2+^ 2p_1/2_ (873.7 eV) (Figure 4f). The corresponding satellite peaks of Fe, Co, and Ni 2p were also observed in the spectra. It is noteworthy that the core-level binding energy shift occurs in all of the constituent elements of PtRuFeCoNi nanoparticles (Table S7), implying the intrinsic charge transfer between elements.^40,41^ In addition, the high-resolution XPS spectra of all metal elements reveal the coexistence of metal and oxidation states, which could be ascribed to the spontaneous formation of the oxide layer on the surface of PtRuFeCoNi nanoparticles upon exposure to air.^42^ Importantly, the elemental ratios derived from XPS analysis are in good agreement with those obtained from bulk-sensitive ICP-OES measurements (Figure S12), indicating that the surface composition is largely representative of the overall nanoparticle composition, with only minor deviations arising from the different probing depths and measurement principles of the two techniques. For comparison, the surface chemical states of the noble-metal-only PdPtRhIrRu HEA nanoparticles were also examined by XPS (Figure S13). In contrast to PtRuFeCoNi, the high-resolution spectra are dominated by metallic components with only minor oxidized contributions, indicating a largely metallic surface.

**Figure 4:**
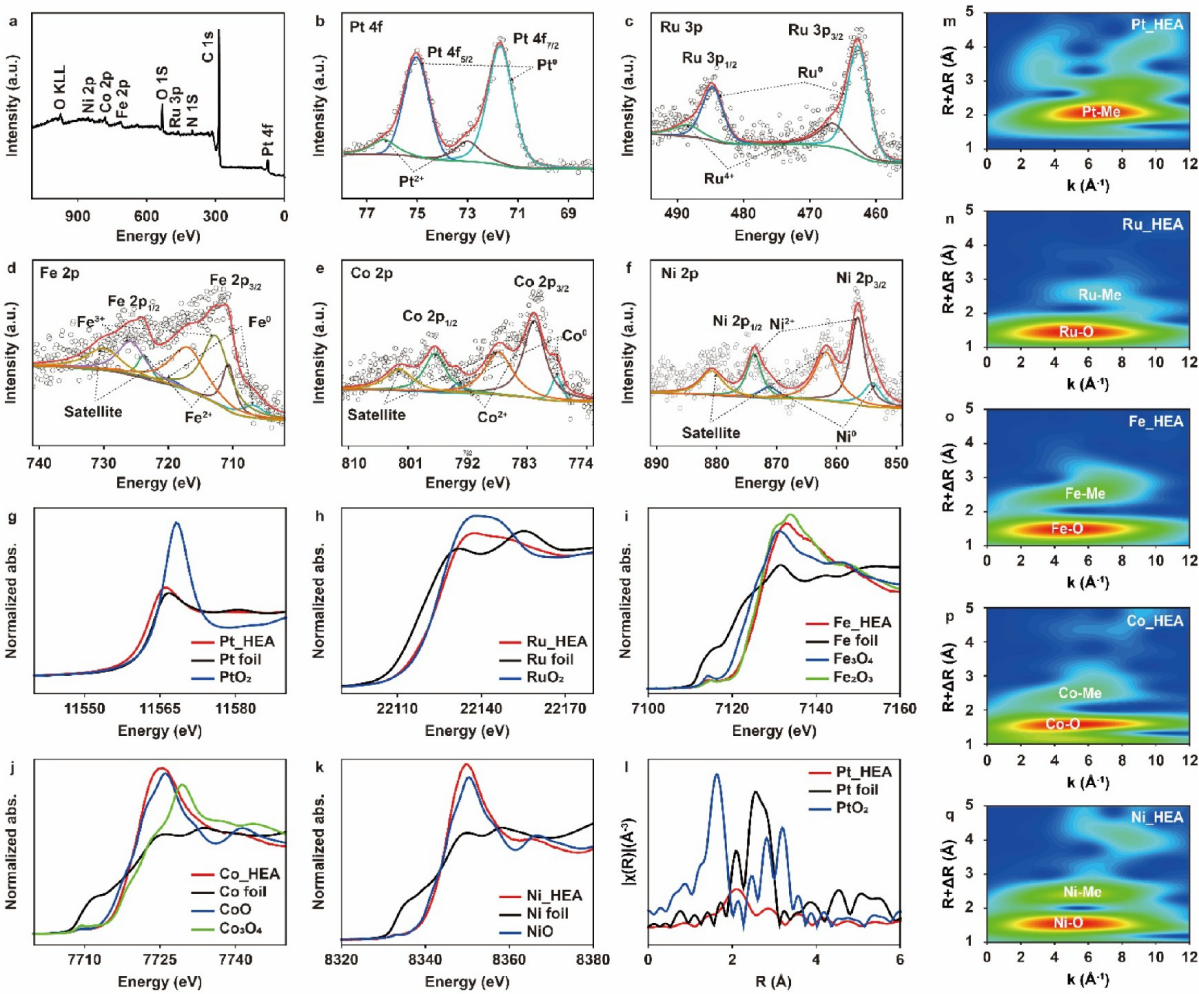
Chemistry and local coordination environment of 1 nm PtRuFeCoNi HEA nanoparticles. (a) Survey XPS spectrum and high-resolution XPS spectrum of (b) Pt 4f, (c) Ru 3p, (d) Fe 2p, (e) Co 2p, and (f) Ni 2p. XANES spectra of PtRuFeCoNi HEA nanoparticles and the corresponding metallic foils and oxides at the (g) Pt L_3_-edge, (h) Ru K-edge, (i) Fe K-edge, (j) Co K-edge, and (k) Ni K-edge. (l) FT-EXAFS spectra of Pt in 1 nm PtRuFeCoNi HEA nanoparticles along with the corresponding metallic foils and oxides. (m to q) WT-EXAFS analysis of PtRuFeCoNi HEA nanoparticles. (m) Pt L_3_-edge, (n) Ru K-edge, (o) Fe K-edge, (p) Co K-edge, and (q) Ni K-edge.

Furthermore, the electronic structures and local coordination environments of PtRuFeCoNi nanoparticles and their corresponding pure metals and oxides as references were analyzed by synchrotron XAS analysis. In the X-ray absorption near-edge structure (XANES) spectra, Pt in the 1 nm HEA nanoparticles was examined by integrating the area of the white-line peak in the L_3_-edge XANES spectra (Figure S14), which indicates its presence in a valence state of approximately +0.65, as compared to reference samples of pure Pt foil. The absorption K-edge positions of Ru, Fe, Co, and Ni elements in the 1 nm HEA nanoparticles were also determined by identifying the inflection points of the pre-edges (Figure S15). Subsequently, the chemical states were derived by establishing a linear relationship between the edge positions and the chemical states of the control samples including metallic foils and oxides (Figure S16). The near-edge shifts observed at the Ru, Fe, Co, and Ni K-edges imply that these elements exhibit positive oxidation states under ambient conditions. This phenomenon is likely influenced by the large surface-to-volume ratio of the 1 nm nanoparticles and the inherently oxidizing nature of these elements (Figure 4h–k), which in agreement with the above XPS results. The aforementioned results highlight the redistribution of electrons and the precise tuning of the electronic structure within the PtRuFeCoNi HEA nanoparticles.^43^

The extended X-ray absorption fine structure (EXAFS) spectra of five elements with 1 nm HEA nanoparticles were provided in Figures 4l and S17. To gain a deeper understanding of the high-entropy mixing state within the 1 nm PtRuFeCoNi HEA nanoparticles, FT-EXAFS spectra fitting for the five mixed elements were performed. This analysis provided insights into the radial distances and local coordination environments of the mixed elements in the 1 nm PtRuFeCoNi nanoparticles (Figures S18, S19, Table S8, and S9). Based on the fitting results, the radial distance of Pt within the PtRuFeCoNi HEA nanoparticles is 2.78 Å for Pt–Pt coordination, which is slightly longer than that of Pt–Ru (2.75 Å) and Pt-3d (2.62 Å). In addition, Pt–O bonds emerge at 2.03 Å, indicating partial surface oxidation. Notably, the Pt–Pt radial distance (2.78 Å) is marginally longer than that in Pt foil (2.76 Å). This slight expansion can be reasonably attributed to pronounced lattice distortion effects in ultrasmall (∼1 nm) HEA nanoparticles, where reduced coordination numbers, high surface-to-volume ratios, and severe atomic-scale strain lead to deviations from bulk bond lengths. For Ru, the Ru–Pt and Ru–Ru radial distances are approximately 2.88 Å and 2.73 Å, respectively. The Ru-3d coordination distance (2.58 Å) is noticeably longer than the corresponding metal–metal distances in 3d transition-metal foils (Fe–Fe: 2.46 Å; Co–Co: 2.48 Å; Ni–Ni: 2.47 Å), while Ru–O bonds appear at 1.99 Å. These results indicate that the local coordination environment of Ru is strongly perturbed by heteroatomic interactions and surface oxidation within the HEA framework. For the IGM elements (Fe, Co, and Ni), the radial distances of Fe–Pt, Fe–Ru, Co–Pt, Co–Ru, Ni–Pt, and Ni–Ru are all significantly longer than those in the corresponding metallic foils, suggesting the formation of alloyed metallic bonds between 3d metals and PGM atoms (Pt and Ru). In contrast, the Fe-3d, Co-3d, and Ni-3d coordination distances are broadly distributed around 3.04–3.08 Å, accompanied by Fe–O, Co–O, and Ni–O bonds at approximately 2.01–2.14 Å, indicating partial oxidation of 3d metals.^44^ Overall, the coexistence of both expanded and contracted bond lengths across different atomic pairs reflects severe lattice distortion in the ultrasmall PtRuFeCoNi HEA nanoparticles. Such bond-length heterogeneity is a characteristic feature of high-entropy systems at the subnanometer scale and arises from combined effects of atomic size mismatch, compositional complexity, and size-induced strain.

The local coordination environments of the constituent elements in the PtRuFeCoNi HEA nanoparticles were elucidated by EXAFS fitting. The coordination numbers (CNs) for Me-Pt/Me-Ru/Me-3d interactions are determined as 2.5/1.6/1.7 for Pt, 1.4/2.8/1.5 for Ru, 1.7/1.3/2.5 for Fe, 2.7/0.9/2.2 for Co, and 2.0/2.0/2.1 for Ni. These results clearly demonstrate extensive intermetallic bonding among Pt, Ru, and 3d elements, supporting a homogeneous high-entropy mixing state rather than elemental segregation within the nanoparticles. The coordination number of Pt–O bonds is relatively low (CN = 0.7) compared with those of Pt–Pt, Pt–Ru, and Pt-3d pairs, indicating that Pt remains predominantly in a metallic state with only minor surface oxidation. In contrast, Ru, Fe, Co, and Ni exhibit comparable coordination numbers for metallic and oxygen-coordinated bonds, suggesting the coexistence of metallic and partially oxidized environments. For example, the summed metallic coordination of Fe (Fe–Pt + Fe–Ru + Fe–3d ≈ 5.5) is comparable to its Fe–O coordination (≈4.5), consistent with partial oxidation of 3d metals. In the fitting model, taking Fe as an example, the 12 nearest-neighbor sites in the Fe-centered first shell were initialized as 3 Pt, 3 Ru, and 6 3d-metal neighbors. Because Fe, Co, and Ni have highly similar EXAFS backscattering functions and strong parameter correlations, their contributions were grouped into a single Fe-3d term, and a Fe–O contribution was further included based on the XANES results. These results are consistent with the XPS results (Figure 4). Additionally, the coordination numbers of all five elements in the HEA nanoparticles are lower than those of their foil references, reflecting the exceptionally small size of these HEA nanoparticles. The partial oxidation could be attributed to the spontaneous oxide formation on the surface of PtRuFeCoNi nanoparticles upon exposure to air, a phenomenon observed in several studies on HEA nanoparticles, particularly those containing easily oxidized elements such as Fe, Co, and Ni.^15,42^ Again, the wavelet analysis of the k^2^-weighted EXAFS (WT-EXAFS) shows the significant difference in local coordination environments between PtRuFeCoNi nanoparticles (Figure 4m–q) and the corresponding references (Figure S20), showing all the elements were surrounded by different species.

### Autocatalytic Reduction for Crafting 1 nm HEA Nanoparticle Family

It is worth noting that the standard reduction potentials for Pt­(IV), Ru­(III), Fe­(III), Co­(II), and Ni­(II) precursors are 0.680, 0.758, −0.037, −0.280, and −0.257 V_SHE_, respectively. These values suggest significantly different reducibilities, which may result in varying reduction rates. This discrepancy may make it easier to form phase-separated nanoparticles rather than the high-entropy mixing nanoparticles obtained in this work. To clarify this issue and investigate the reduction kinetics of metal precursors, and consequently, the growth mechanism of HEA nanoparticles confined in the mesopore space of N-doped CMK-3, the H_2_ temperature-programmed reduction (H_2_-TPR) profiles were obtained from the monocomponent Pt, Pd, Ru, Rh, Fe, Co, Ni, Mo, Zn, and Cu, ternary FeCoNi, quaternary PtFeCoNi, PdFeCoNi, RuFeCoNi, and RhFeCoNi, quinary PtRuFeCoNi, and denary PtRuFeCoNiCuMoRhPdZn nanoparticles, as shown in Figures 5a, S21, S22, and S23. Due to the high reduction potentials (Table S3), Pt­(IV), Pd­(II), Ru­(III), and Rh­(III) metal precursors were reduced at low temperatures of around 122, 153, 156, and 130 °C, respectively, while the other metal precursors reduced at higher temperatures (Figure S21). The reduction of ternary-component precursors including mixed Fe­(III), Co­(II), and Ni­(II) ions with relatively low reduction potentials started at high temperature and was continuously increased up to a maximum at 436 °C. The quaternary-component precursors for the RuFeCoNi and RhFeCoNi nanoparticles displayed low-temperature peaks, with broad and multiple peaks ranging from 120 to 430 °C. This indicates that Ru and Rh could catalyze the partial reduction of Fe­(III), Co­(II), and Ni­(II) precursors. Most importantly, with the further introduction of the Pt precursor for the equimolar PtRuFeCoNi nanoparticles, the quinary-component precursors showed only a single, sharp reduction peak with a maximum temperature of 189 °C, suggesting a synchronized and highly cooperative coreduction behavior of the five mixed-metal precursors. To elucidate the role of the noble metal elements in directing this low-temperature coreduction via an autocatalytic pathway, a control experiment was performed using a noble-metal-diluted Pt_0.05_Ru_0.05_Fe_0.3_Co_0.3_Ni_0.3_ (Figure S22f). Crucially, upon decreasing the relative content of Pt and Ru, the single-step reduction behavior was disrupted and split into a distinct multistage profile, where the initial reduction onset was delayed to 206 °C, followed by the emergence of a massive, broad reduction peak centered at 386 °C. This dramatic high-temperature shift and peak split upon noble-metal dilution firmly demonstrate that the early stage reduced Pt/Ru species serve as indispensable catalytic seeds that pull the reduction of the remaining 3d transition metal precursors (Fe, Co, and Ni) down to lower temperatures. When these seeds are intentionally diluted, the autocatalytic efficiency drops significantly, forcing the transition metals to undergo reduction at their intrinsic, much higher thermal regimes.

**Figure 5:**
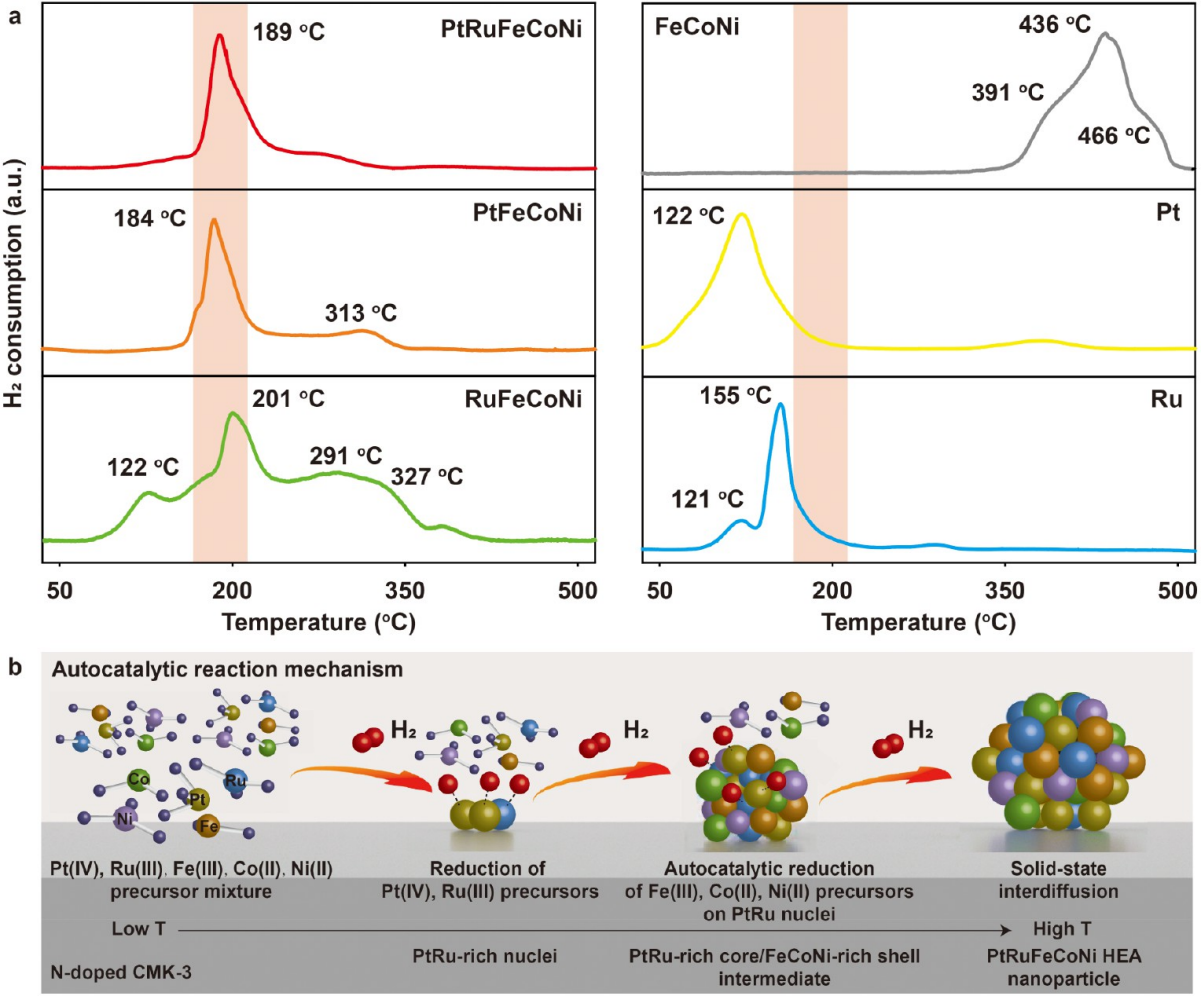
Autocatalytic reaction mechanism of 1 nm PtRuFeCoNi HEA nanoparticles. (a) H_2_-TPR profiles for the mono-, ternary, quaternary, and quinary component samples after the impregnation of the metal precursors into N-doped CMK-3. (b) Schematic diagram of the formation mechanism of PtRuFeCoNi HEA nanoparticles under 10% H_2/_90% N_2_ atmosphere.

To further realize the subsequent structural evolution driven by this sequential reduction, intermediate-state quenching experiments were performed. Because the PtRuFeCoNi nanoparticles embedded in N-doped CMK-3 are extremely small, with an average size of 1.12 ± 0.13 nm, their diffraction signals are too weak for reliable XRD-based intermediate-state analysis (Figures S7 and S8). Therefore, Vulcan XC-72R carbon was strategically employed as a model support for these control experiments. Under otherwise identical reduction conditions, the absence of strong spatial confinement on Vulcan XC-72R leads to larger PtRuFeCoNi nanoparticles with an average size of 3.96 ± 1.68 nm (Figure 3k–n), providing sufficient diffraction intensity to monitor phase evolution by XRD (Figure S23). As shown in the phase-tracking XRD patterns (Figure S23), the intermediate state isolated after holding at 150 °C for 30 min shows a broad diffraction peak at 2θ = 40.78°, which can be assigned to the FCC(111) reflection and corresponds to a relatively large lattice spacing. This feature is consistent with the preferential reduction and nucleation of a Pt/Ru-rich intermediate phase (Pt 1.39 Å and Ru 1.34 Å), in agreement with the initial reduction onset observed in H_2_-TPR. After full thermal treatment at 450 °C, the diffraction peak of FCC(111) intensifies and shifts to a higher angle of 2θ = 41.57°, indicating lattice contraction. This shift supports the thermal interdiffusion and incorporation of smaller 3d transition metals, including Fe, Co, and Ni (Fe = 1.26 Å, Co = 1.25 Å, and Ni = 1.24 Å), into the parent lattice, leading to the formation of a more homogenized FCC HEA solid-solution phase.

Based on these experiments, the corresponding formation mechanism of the PtRuFeCoNi nanoparticles could be illustrated in Figure 5b. The synthesis of quinary PtRuFeCoNi nanoparticles proceeds through a staged pathway directed by a strategic thermal protocol. It begins with a hold at 150 °C under 10% H_2/_90% N_2_, which promotes the early reduction of Pt­(IV) and Ru­(III) precursors to form Pt/Ru nuclei that serve as efficient H_2_ activation sites. As the temperature ramps from 150 to 450 °C at 10 °C min^–1^, these preformed nuclei trigger the autocatalytic reduction of the Fe­(III), Co­(II), and Ni­(II) precursors, resulting in the concurrent nucleation and growth of nanoparticles containing all five elements. This staged interpretation is corroborated by H_2_-TPR results showing a single sharp reduction feature at 189 °C, which is consistent with a synchronized reduction of all precursors once metallic nuclei are present to activate H_2_. Finally, annealing at 450 °C for 120 min facilitates thermally activated atomic diffusion, a process that progressively erases transient compositional gradients and drives the system toward a chemically homogeneous HEA phase (Figure 2) rather than a segregated core–shell structure.

To further verify that such synchronized reduction behavior is an intrinsic feature of HEA formation rather than a peculiarity associated with 3d metals, we additionally performed H_2_-TPR analysis on a noble-metal-only HEA system, PdPtRhIrRu (Figure S24). While the monometallic Pd, Pt, Rh, Ir, and Ru precursors exhibit distinct and element-specific reduction temperatures (ranging from ∼ 99 to 167 °C), the PdPtRhIrRu HEA nanoparticles display a single dominant reduction peak centered at 142 °C. This temperature lies intermediate to those of the constituent monometallic catalysts, indicating that strong interelement interactions within the HEA framework significantly modify the individual reduction behaviors and drive a cooperative reduction process. Notably, despite the absence of low-potential 3d metals, the noble-metal HEA still exhibits a narrowed reduction window, further confirming that entropy-driven mixing and intermetallic interactions play a decisive role in synchronizing reduction kinetics. Similar reduction kinetics with a narrow reduction temperature window of about 198 °C were also observed for the denary PtRuFeCoNiCuMoRhPdZn nanoparticles (Figure S22), suggesting the autocatalytic reduction facilitated by the catalytic metals during the formation of nanoparticles. These results indicate that even though certain single metal precursors, like pure Fe­(III), Co­(II), and Cu­(II), have reduction temperatures exceeding 450 °C, the presence of catalytic elements during synthesis can significantly lower the reduction temperature, causing the reduction to occur within a much lower and specific temperature range. To further verify the completeness of precursor reduction during synthesis, control H_2_-TPR experiments were conducted (Figure S25). When the fully reduced PtRuFeCoNi precursors were cooled to room temperature under an Ar atmosphere and immediately subjected to a second TPR measurement without air exposure, no discernible H_2_ consumption peaks were observed over the entire temperature range, confirming that the metal precursors had been completely reduced during the initial treatment. In contrast, samples intentionally exposed to air prior to the second TPR measurement exhibited distinct low- and intermediate-temperature reduction peaks, which can be attributed to surface reoxidation of oxophilic Fe, Co, and Ni species. These results unambiguously demonstrate that the observed oxidation arises from postsynthesis air exposure rather than incomplete reduction during the formation of PtRuFeCoNi HEA nanoparticles.

In addition, to investigate the importance of autocatalytic reduction in the synthesis of HEA nanoparticles in a forming gas environment, we conducted control experiments for the synthesis of quinary PtRuFeCoNi nanoparticles at four representative temperatures (250, 450, 650, and 850 °C) in the Ar atmosphere. Importantly, upon switching the atmosphere environment from forming gas to argon, we observed significant changes in the size and dispersion of the PtRuFeCoNi nanoparticles at all temperatures (Figure S26). These alterations resulted in a much larger particle size (∼5–50 nm) and nonuniform particle dispersion. Figure S27a shows the low-magnification HAADF-STEM image of PtRuFeCoNi nanoparticles synthesized at 450 °C in the Ar atmosphere. The resultant PtRuFeCoNi nanoparticles diffused outside the porous structure of N-doped CMK-3 and thus sintered into larger particles. Importantly, the phase separation with clear phase boundaries of the single nanoparticle was observed in the atomic-resolution HAADF-STEM image, as shown in Figure S27b. The brightest region at the core could be attributed to the presence of a Pt-based phase due to the highest Z-contrast of Pt in the nanoparticle. In addition, as shown in Figure S27c, the XRD pattern exhibits the diffraction peaks at 2θ values of 39.8, 46.5, and 67.8°, which matches perfectly with the 111, 200, and 220 characteristic peaks of FCC Pt, respectively. There is a small shoulder peak from the Ru, Fe, Co, and Ni metals in the range of 41.2–44.5°. The EDS mappings (Figure S27d and e) also reveal that the Pt element forms a core structure, while other metal elements are inhomogeneously distributed in the outer layers. This result could be attributed to the relatively low pyrolysis temperature of Pt­(II) precursor compared with the other four metal precursors.^45–48^ These results suggest the phase separation of PtRuFeCoNi nanoparticles synthesized at 450 °C in the Ar atmosphere.

The pyrolysis process of the metal-precursor mixture was further tracked by thermogravimetric analysis (TGA) and differential scanning calorimetry (DSC) analysis in the Ar atmosphere (Figure S28). The weight loss and endothermal signals show continuous drops rather than a distinct peak at a specific temperature, revealing that the thermal decomposition with dehydration and dichlorination of the metal-precursor mixture was over a rather wide temperature range from 300 to 650 °C. Since the thermal decomposition temperatures of the metal precursors are much higher than the reduction temperatures (Table S10), the metal precursors may diffuse outside the mesopores before reaching the thermal decomposition temperature during the thermal annealing treatments and thus pyrolyze at the surface of N-doped CMK-3, resulting in greater particle size and a nonuniform dispersion. Taken together, when the atmosphere was switched from forming gas (10% H_2/_90% N_2_) to Ar, the synthesis mechanism shifted from autocatalytic reduction to thermal decomposition of the metal-precursor mixture. This conclusion is supported by TGA and DSC analyses conducted in an Ar atmosphere (Figure S28). These results also emphasize the importance of autocatalytic reduction of metal-precursor mixture in the synthesis of HEA nanoparticles in the forming gas, which can significantly reduce the annealing temperature to avoid the diffusion of metal precursors and/or atoms out of the mesopores and thus the achievement of size reduction down to 1 nm.

Furthermore, we showcase the universality of template-confined synthesis for 1 nm HEA nanoparticles via autocatalytic reduction at 450 °C in the forming gas, as shown in Figures 6 and S29. The identical protocols were employed for preparing various HEA nanoparticles incorporating up to ten mixed elements, with the only variations being the types and concentrations of the metal precursors utilized. In particular, we selected at least two catalytic elements (e.g., Pt, Ru, Rh, and Pd) to form HEA nanoparticles with other elements, as these two elements can catalyze the autocatalytic reduction of other metal precursors with relatively lower reduction potentials (e.g., Fe­(III), Co­(II), Ni­(II), Cu­(II), Mo­(V), and Zn­(II)). We generated a family of ultrasmall HEA nanoparticles, including quinary (PtRuFeCoNi, PtPdFeCoNi, PdRhFeCoNi, PtRhFeCoNi, RuPdFeCoNi, and RuRhFeCoNi; Figure 6a–e), senary (PtRuFeCoNiCu; Figure 6f), septenary (PtPdFeCoNiCuMo; Figure 6g), octonary (PtRuFeCoNiCuMoRh; Figure 6h), nonary (PtRuFeCoNiCuMoRhPd; Figure 6i), and denary (PtRuFeCoNiCuMoRhPdZn; Figure 6j) HEA nanoparticles. Their TEM images, ICP-OES results, and EDS elemental mappings confirmed the high-entropy mixing state of HEA nanoparticles dispersed uniformly in the N-doped CMK-3. Importantly, all of the nanoparticles have a size of approximately 1 nm with a narrow size distribution, as confirmed by the statistical size distributions.

**Figure 6:**
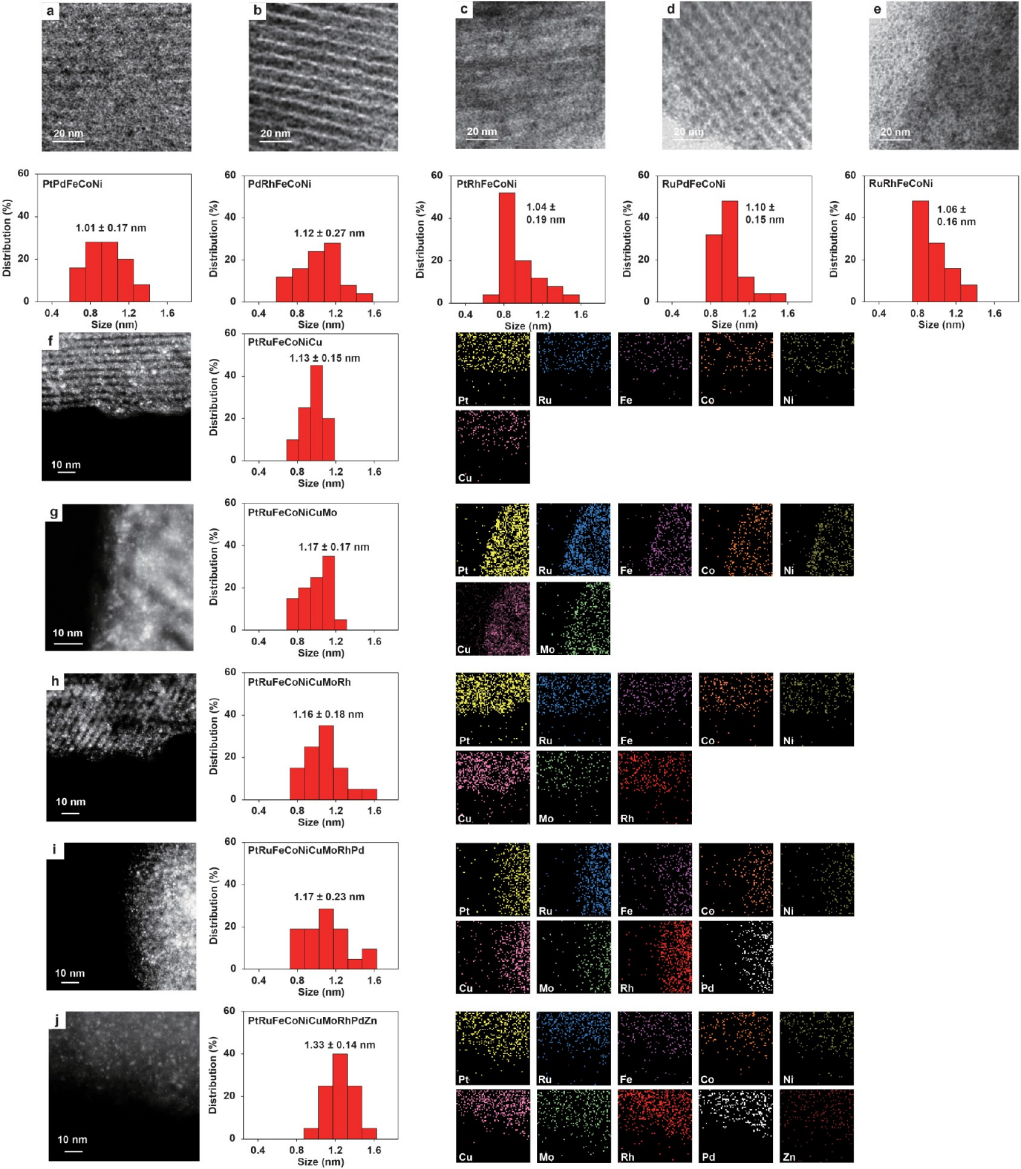
Autocatalytic reaction mechanism for crafting 1 nm HEA nanoparticle family. TEM images and particle size distributions of quinary (a) PtPdFeCoNi, (b) PdRhFeCoNi, (c) PtRhFeCoNi, (d) RuPdFeCoNi, and (e) RuRhFeCoNi. HAADF-STEM images, particle size distributions, and EDS mappings of (f) senary PtRuFeCoNiCu, (g) senary PtRuFeCoNiCuMo, (h) septenary PtRuFeCoNiCuMoRh, (i) nonary PtRuFeCoNiCuMoRhPd, and (j) denary PtRuFeCoNiCuMoRhPdZn.

### Catalytic Performance and Synergistic Effect Revealed by *Operando* XAS

As a proof of concept, six different quinary 1 nm sized PtRuFeCoNi, PtPdFeCoNi, PdRhFeCoNi, PtRhFeCoNi, RuPdFeCoNi, and RuRhFeCoNi nanoparticles by mixing three elements of iron-group metals (IGMs; Fe, Co, and Ni) with two elements of platinum-group metals (PGMs; Pt, Pd, Rh, and Ru) were used as electrocatalysts to examine the catalytic performances for HER and HOR, as shown in Figure 7. The reproducibility is also shown in Figure S30. A summary of the electrocatalytic HER and HOR measurements, including the specific protocols, potential ranges, and electrolyte conditions, can be found in Tables S11. The alloying of PGMs with earth-abundant IGMs to form single-phase HEA nanoparticles has garnered significant attention. These HEA nanoparticles, serving as catalysts, have the potential to reduce the reliance on PGMs. Additionally, the substantial difference in electronegativity between IGMs and PGMs may lead to electronic interactions among the mixed elements, thereby generating a strong synergistic effect. Very recent, theoretical calculations also suggest that localized electronic redistributions and orbital hybridizations between these 3d, 4d, and 5d transition metals in the HEA nanoparticles may result in preferential adsorption sites for reactants, intermediates, and/or products.^13,14^ This phenomenon contributes to enhanced catalytic performance. Therefore, in this study, we strategically selected a combination of IGMs mixed with PGMs to form HEA nanoparticles with an extremely small size of 1 nm for HER and HOR, with the goal of further enhancing mass activity and atom utilization.

**Figure 7:**
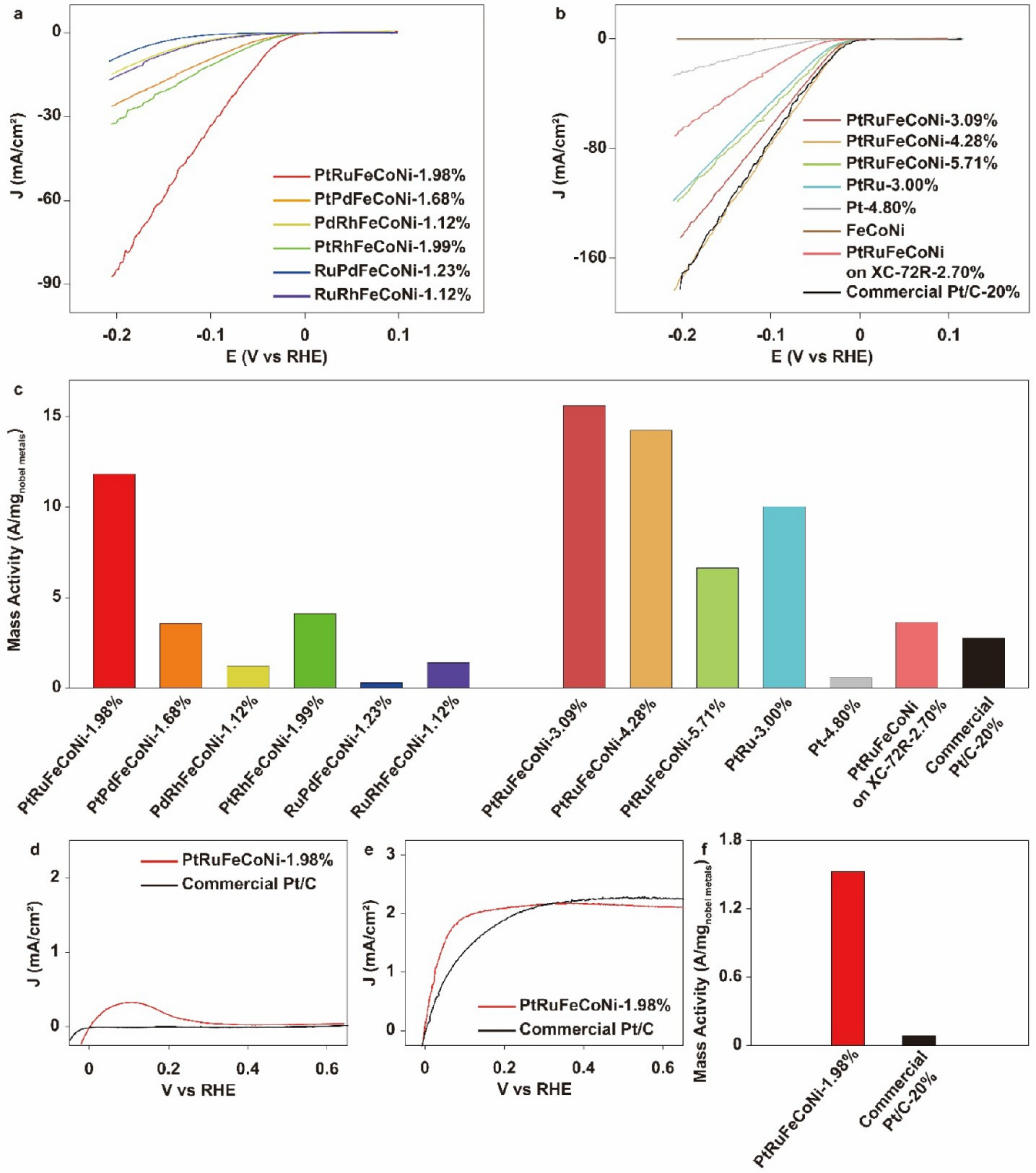
Catalytic performances of 1 nm HEA nanoparticles. (a to c) Electrocatalytic HER in the 0.5 M H_2_SO_4_. Polarization curves of (a) different composition and (b) different noble-metal loading. (c) Mass activities of all the catalysts under −0.05 V. (d to f) Electrocatalytic HOR in the 0.1 M KOH. Polarization curves at (d) N_2_-saturated and (e) H_2_-saturated atmosphere. (f) Mass activities of the catalysts under 0.05 V. Note that the symbol “%” following the labels represents the weight percentage of the noble metals in the catalysts.

In the first set of catalytic measurements, we surveyed the HER performance of six different quinary 1 nm HEA nanoparticles with the noble-metal loading of around 1–2 wt % embedded in N-doped CMK-3 support in acidic 0.5 M H_2_SO_4_ electrolyte. Figure 7a shows the linear sweep voltammetry (LSV) curves using a standard three-electrode system (where the current is normalized by geometric electrode area). Among these HEA catalysts, the PtRuFeCoNi nanoparticles with 1.98 wt % noble-metal loading (denoted as PtRuFeCoNi-1.98% in Figure 7) exhibit the smallest overpotential of 46 mV at a current density of 10 mA cm^–2^. Given the good performance, we further prepared the PtRuFeCoNi nanoparticles with higher noble-metal loadings of 3.09, 4.28, and 5.71 wt % embedded in N-doped CMK-3 support to find the optimal loading amount to maximize the utilization efficiency of atoms in the second set of catalytic measurements. Also, monometallic Pt, bimetallic PtRu, and trimetallic FeCoNi nanoparticles embedded in the N-doped CMK-3 as well as the commercial Pt/C and the PtRuFeCoNi nanoparticles prepared on the commonly used XC-72R carbon support were tested for comparison. As shown in Figure 7b, the PtRuFeCoNi nanoparticles with a noble-metal loading of 4.28 wt % exhibited the highest current density of 77.8 mA cm^–2^ at −0.1 V_RHE_ among all the catalysts. This performance is slightly superior to that of the commercial Pt/C catalyst, which has a particle size range of 2–3 nm and a high noble-metal loading of 20 wt % (74.3 mA cm^–2^ at −0.1 V) on the carbon support under identical testing conditions. To quantitatively compare the HER activity of each catalyst, mass activities were normalized based on the precise noble-metal loading amounts determined by ICP-OES analysis (Table S12). As shown in Figure 7c and Table S13, the PtRuFeCoNi-3.09% nanoparticles reach the highest mass activity of 15.5 A mg^–1^_Pt+Ru_ among all the catalysts with different compositions and loadings, which is 6.4 times higher than that of the commercial Pt/C (2.5 A mg^–1^_Pt_). In addition, they also show 4.2-time better mass activity than the PtRuFeCoNi nanoparticles (3.8 A mg^–1^_Pt+Ru_) prepared on the XC-72R carbon support, further suggesting that the size reduction of HEA nanoparticles down to 1 nm could greatly enhance the utilization efficiency of atoms. By comparing the mass activities of the 1 nm PtRuFeCoNi HEA nanoparticles (15.5 A mg^–1^_Pt+Ru_) obtained in this work with other advanced noble metal-based and HEA catalysts reported in recent literature (Table S14) for HER in acidic 0.5 M H_2_SO_4_ electrolyte at various applied potentials, it can be concluded that the 1 nm PtRuFeCoNi HEA nanoparticles rank among the top performers and exhibit highly competitive activity. In addition, the electrochemically active surface areas (ECSAs) were determined by CO-stripping experiments (Figures S31 and S32), and the HER currents were normalized to the corresponding ECSA values to obtain the specific activities (Figure S33). After ECSA normalization, the PtRuFeCoNi-based catalyst still exhibits the highest specific activity among the tested catalysts. To further elucidate the reaction kinetics, Tafel slopes were analyzed for PtRuFeCoNi catalysts with different loadings (Figure S34). The PtRuFeCoNi catalyst with a 3.09 wt % noble-metal loading exhibited the lowest Tafel slope of 25.9 mV dec^–1^, consistent with a Tafel-determining step and an overall Volmer–Tafel HER pathway.^47^

In the second set of catalytic measurements, we examined the catalytic performances of representative 1 nm PtRuFeCoNi nanoparticles and commercial Pt/C catalysts for the alkaline HOR, a critical process in anion-exchange membrane fuel cell technology. The HOR performances were evaluated through linear sweep voltammetry (LSV) polarization curves in both N_2_ and H_2_-saturated 0.1 M KOH solution environments. As shown in Figure 7d, a minimal anodic current was observed in the N_2_-saturated electrolyte. Conversely, a significant anodic current starting from 0 V_RHE_ was recorded upon H_2_ saturation of the electrolyte, indicating the initiation of hydrogen oxidation (Figure 7e). Notably, the 1 nm PtRuFeCoNi nanoparticles exhibited lower HOR onset overpotentials compared with the commercial Pt/C catalysts. While both catalysts approach similar diffusion-limiting current plateaus at higher overpotentials due to mass-transport constraints, the intrinsic electron-transfer kinetics of PtRuFeCoN are substantially accelerated. To extract the pure kinetic descriptors decoupled from these diffusion limitations, a rigorous mass-transport correction was applied to the rotation-speed-dependent polarization curves (Figure S35). The decoupled kinetic current densities (J_k_) were subsequently analyzed via Tafel plots (Figure S36). Crucially, the 1 nm PtRuFeCoNi nanoparticles manifest a significantly lowered Tafel slope of 94.6 mV dec^–1^ compared to that of commercial Pt/C (120.2 mV dec^–1^), highlighting a drastically reduced kinetic barrier for interfacial charge transfer. To establish the most definitive intrinsic parameter, the exchange current densities (j_0_) were quantitatively evaluated by fitting the linear micropotential region (20 mV_RHE_) using the simplified Butler–Volmer relation (Figure S37). Benefiting from the electronically optimized multielement configurations, the 1 nm PtRuFeCoNi HEA catalyst yields an outstanding j_0_ value of 1.06 mA cm^–2^, which nearly doubles that of the commercial Pt/C benchmark (0.56 mA cm^–2^). This intrinsic kinetic superiority is further complemented by its outstanding macroscopic performance; as plotted in Figure 7f, the mass activity determined at a potential of 0.05 V_RHE_ for the 1 nm PtRuFeCoNi nanoparticles (1.5 A mg^–1^_Pt+Ru_) far surpassed that of the commercial Pt/C (0.09 A mg^–1^_Pt_).

To unravel the mechanism behind this alkaline HOR acceleration, electrochemical CO stripping voltammetry was conducted to probe the interfacial oxophilicity (Figure S38). In the Langmuir–Hinshelwood framework of alkaline HOR, the removal of H* intermediates relies on the timely, low-potential adsorption of OH* species to achieve bifunctional synergy. As resolved in the stripping profiles, commercial Pt/C-20% exhibits a sharp, symmetric anodic oxidation peak centered at 0.66 V_RHE_, indicating synchronized CO clearing on a periodic, single-site metallic surface. Conversely, the PtRuFeCoNi-1.98% manifests an exceptionally flattened and broad oxidation envelope spanning from a premature onset of 0.42 V_RHE_ without prominent current spikes. This distinct CO-stripping profile suggests a broad distribution of OH-associated adsorption environments across diverse local coordinated ensembles. In the quinary HEA lattice, the atomic-level mixing of Pt with oxophilic Fe, Co, Ni, and Ru may facilitate OH* formation at lower overpotentials. These OH* species can then react with CO* adsorbed on neighboring Pt-containing sites. Because different local configurations may become activated over a range of potentials, their overlapping oxidation responses can lead to the broad and flattened CO-stripping profile. This behavior implies that the chemical heterogeneity of the high-entropy configuration provides a multisite adsorption landscape, which may contribute to H/OH-related bifunctional behavior during alkaline HOR.

These findings show that the 1 nm PtRuFeCoNi nanoparticles supported on the mesoporous carbon network act as efficient electrocatalysts for both the HER and HOR with improved noble-metal atom utilization. To evaluate the possible contribution from the support itself, the pristine N-doped CMK-3 matrix was examined as a baseline control (Figure S39). The pure N-doped CMK-3 shows negligible catalytic response toward both the HER and HOR, indicating that the carbon support itself is not the primary origin of the observed activity. Nevertheless, the enhanced performance should not be attributed solely to the HEA effect, but rather to the combined contributions of the ultrasmall particle size, high metal dispersion, possible confinement effects, and multielement alloying within the PtRuFeCoNi nanoparticles.

To evaluate electrochemical durability, long-term chronoamperometric (CA) measurements were conducted for both HER and HOR conditions. For acidic HER (Figure S40), CA tests were performed at −0.07 V_RHE_ for 120 h. The 1 nm PtRuFeCoNi HEA nanoparticles showed a modest decrease in current density from approximately −10.9 to −7.5 mA/cm^2^ over 120 h, while PdPtRhIrRu-3.00% remains more constant, changing from approximately −16.7 to −16.1 mA/cm^2^. Although PdPtRhIrRu-3.00% exhibits slightly higher geometric HER activity, its mass activity is lower than that of PtRuFeCoNi-1.98%, suggesting more efficient noble-metal utilization in the Fe/Co/Ni-containing HEA catalyst. Although some current decay was observed, both catalysts maintained acceptable current retention over prolonged operation, and postreaction characterizations indicated notable differences in their structural evolution (Figure S41). HAADF-STEM analysis showed particle coarsening of PtRuFeCoNi after 120 h of HER operation, accompanied by decreases in the Fe, Co, and Ni atomic fractions, as confirmed by ICP-OES, consistent with the preferential dissolution of 3d transition metals under acidic conditions.

In contrast, the noble-metal-only control catalyst, PdPtRhIrRu, maintained a near-equiatomic composition and showed minimal particle growth after the stability test, indicating its higher resistance to acidic dissolution due to the absence of acid-soluble 3d transition metals. This comparison highlights an important trade-off between improving mass activity through the incorporation of earth-abundant 3d elements and maintaining compositional stability under harsh acidic HER conditions. For the PtRuFeCoNi catalyst, the decreased Fe, Co, and Ni fractions after the prolonged 120 h acidic HER test suggest preferential dissolution of less noble 3d metals, accompanied by dynamic surface restructuring and relative enrichment of noble-metal components near the surface. Therefore, the HER durability of PtRuFeCoNi should be interpreted as operational stability with compositional evolution rather than complete chemical invariance.

For alkaline HOR durability, chronoamperometric measurements conducted at 0.05 V_RHE_ for 24 h show that PtRuFeCoNi exhibits better activity retention than commercial Pt/C (Figure S42). To further evaluate its durability under dynamic operating conditions, an electrochemical accelerated durability test (ADT) was performed through continuous potential cycling up to 3000 cycles, with HOR polarization curves recorded at selected intervals (Figure S43). After 3000 cycles, the 1 nm PtRuFeCoNi nanoparticles show a current-density decay of 5.5% at 0.1 V_RHE_, whereas commercial Pt/C-20% shows a larger decay of 20.0% under the same testing conditions. These results indicate that PtRuFeCoNi possesses improved resistance to potential-cycling-induced performance degradation during alkaline HOR. Collectively, these results indicate that PtRuFeCoNi HEA nanoparticles exhibit competitive operational stability for HER and improved potential-cycling durability for HOR relative to commercial Pt/C. Notably, the post-HER analysis reveals particle coarsening and partial dissolution of Fe, Co, and Ni, suggesting that the durability is accompanied by compositional evolution rather than complete chemical invariance. Thus, the observed stability should be attributed to the combined effects of noble-metal utilization, dynamic surface restructuring, nanoscale confinement, and multielement alloying, rather than solely to the high-entropy configuration.^12,15,23,25^

*Operando* XAS was further employed to provide microscopic insights into the catalytic mechanism and to validate the synergistic electronic interactions previously suggested by the kinetic analysis. The measurements focused on the representative 1 nm PtRuFeCoNi nanoparticles during the acidic HER (Figures 8 and S44), with spectra recorded under open circuit potential (OCP) and applied potentials from 0.05 to −0.15 V_RHE_. As shown in Figure S44, the stable edge positions and white-line intensities across *ex-situ* and *in situ* conditions indicate that the average oxidation states of the constituent elements remain largely unchanged, with minor variations attributed to typical surface reconstruction or adsorption processes in the electrolyte. The XANES spectra provided direct evidence of the electronic coupling among the elements. As the potential shifted from 0.05 to −0.05 V_RHE_, the Fe K-edge shifted to higher energy, whereas the Ru K-edge moved in the opposite direction, indicating potential-induced redistribution of electron density at Fe- and Ru-containing local environments (Figure 8a and b). Upon reaching the HER onset potential of −0.15 V_RHE_, the decreased white-line intensity of the Pt L_3_-edge further suggests electron accumulation in the Pt 5d orbitals (Figure 8c). In contrast, the Co and Ni absorption edges remained nearly unchanged during HER operation (Figure 8d and e). These observations collectively suggest potential-induced charge redistribution on the PtRuFeCoNi HEA surface, with Pt- and Ru-containing local environments becoming relatively more electron-rich under HER-relevant conditions. This spectroscopic trend is consistent with the kinetic behavior observed in the Tafel analysis. However, because *operando* XAS alone cannot directly assign the active centers, we interpret the nearly unchanged Co and Ni absorption edges more conservatively as evidence that Co and Ni are mainly involved in modulating the multielement electronic environment, rather than undergoing pronounced potential-dependent redox changes during HER.^49^ This is further supported by control experiments where the PtRuFeCoNi nanoparticles achieved a mass activity (11.8 A mg^–1^_Pt+Ru_) approximately 1.5 times higher than that of the trimetallic PtRuFe counterpart (Figure S34). The incorporation of Co and Ni increases the configurational entropy and induces a fine-tuned electronic redistribution, which lowers the reaction barriers and accelerates HER kinetics. Collectively, the synergistic interplay among the quinary elements, unveiled by *operando* XAS, explains the exceptional catalytic performance and highlights the critical role of high-entropy mixing in advanced catalyst design.^25,39,50,51^

**Figure 8:**
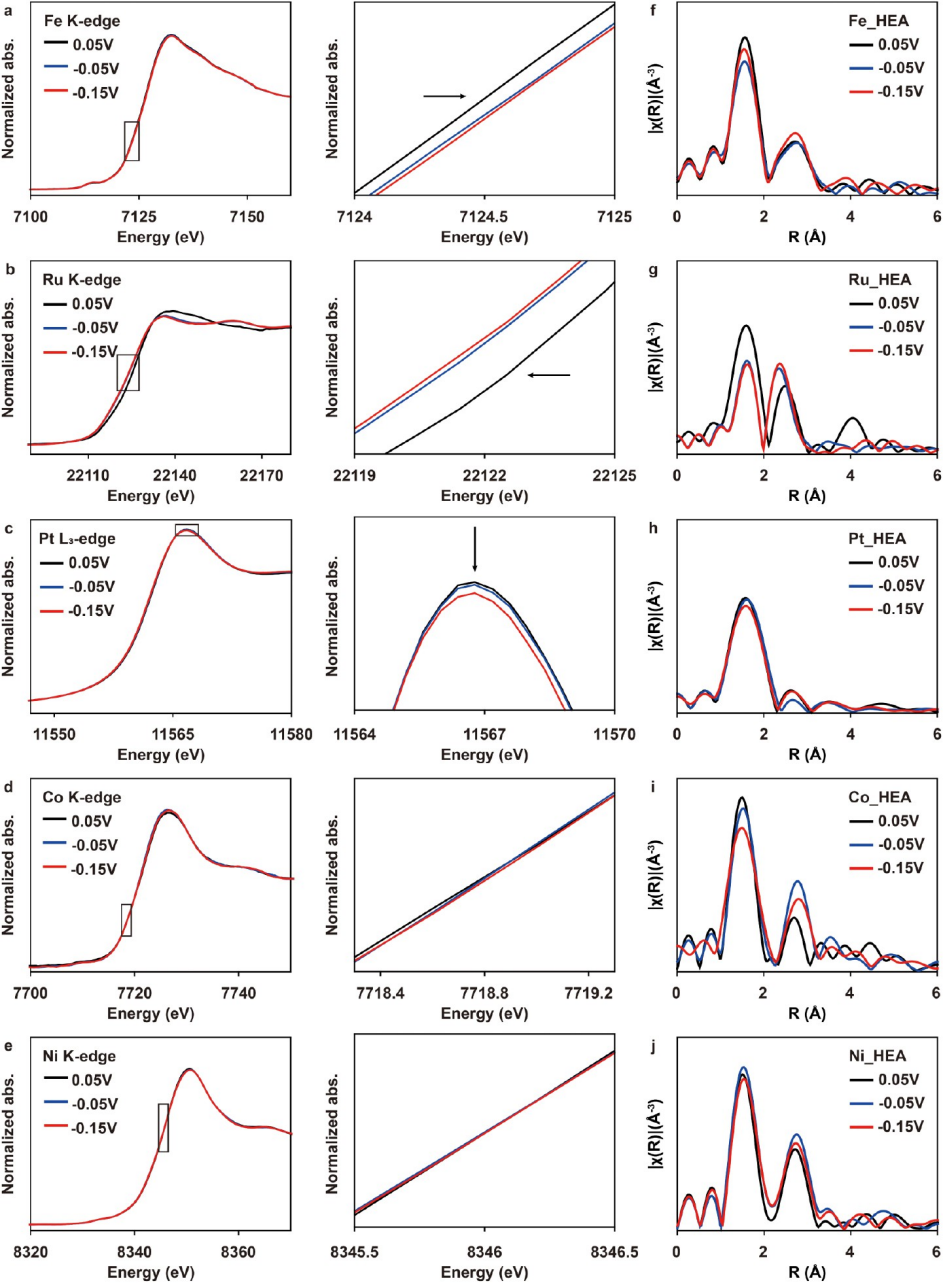
*Operando* XAS analysis for revealing the synergistic effect under HER. XANES spectra of (a) Fe K-edge, (b) Ru K-edge, (c) Pt L_3_-edge, (d) Co K-edge, and (e) Ni K-edge. FT-EXAFS spectra of (f) Fe K-edge, (g) Ru K-edge, (h) Pt L_3_-edge, (i) Co K-edge, and (j) Ni K-edge.

To provide deeper theoretical insights into the exceptional HER performance of the PtRuFeCoNi HEA nanoparticles, extensive DFT simulations were performed (Figure 9 and Figures S45–S52). Initially, the structural integrity of the HEA model was validated using Warren–Cowley short-range order (SRO) parameters (Figure S45). The negative SRO values for heteroatomic pairs and positive values for homoatomic pairs across multiple structural models confirm a chemically homogeneous, well-mixed alloy configuration without elemental clustering, which is essential for cocktail effect. In our DFT calculations, hydrogen adsorption was modeled using the conventional atomic H adsorbate placed on the surface. The hydrogen adsorption free energy (Δ*G*_H*_), a key descriptor for HER activity, was first evaluated on the PtRuFeCoNi (111) slab model (Figure S46). By sampling 180 adsorption configurations (resulting in 154 unique optimized sites), we observed a median Δ*G*_H*_ value of −0.172 eV (Figure 9a). Notably, the most favorable sites were identified as hollow sites (e.g., PtRuFe), where the multiatom interactions yield a near-zero Δ*G*_H*_ of −0.005 eV (Figure 9b). To further elucidate this site-specific variability, we analyzed the statistical distribution of Δ*G*_H*_ across trimeric ensembles (Figure S47). This analysis reveals a broad energy landscape, highlighting the exceptional configurational diversity and the “cocktail effect” inherent to the quinary HEA surface.^3,15,43,52,53^ These findings echo our *operando* XAS observations regarding the electronic synergy between Pt and Ru, as well as the promotional role of Fe. Given the 1 nm size of our nanoparticles, we further employed 55-atom cluster models (SQS-01 to SQS-20) to examine low-coordination environments (Figures S48 and S49). As illustrated in the parity plot (Figure S49a), the hydrogen adsorption free energies (ΔG_H*_) values for the 55-atom cluster are systematically more negative than those obtained from the (111) slab model, confirming that the reduced coordination environment and finite size effects in nanoparticles significantly enhance hydrogen adsorption strength. This trend is further supported by the site-specific analysis in Figure S49b, where even identical trimeric configurations (e.g., CoFeRu or CoRuRu) exhibit stronger binding in the cluster model compared to the periodic slab surface, reflecting the profound influence of the global electronic environment of the nanoparticle. The electronic origin of this optimization was elucidated through projected density of states (PDOS) analysis (Figure S50). The d-band centers of Pt and Ru are positioned at lower energies relative to the Fermi level, facilitating moderate H-binding. In contrast, the iron-group metals (Fe, Co, Ni) exhibit higher d-band centers, acting as electron donors that tune the local electronic environment of the noble metals.^23,30,54,55^ This cooperative electronic modulation, characteristic of the high-entropy state, effectively lowers the reaction barriers. Finally, to evaluate the impact of local surface environments, we further incorporated partial surface oxidation at Fe sites into the slab calculations (Figures S51 and S52). The results show that nearby oxygen coordination weakens hydrogen adsorption. This effect arises because oxygen preferentially binds to Fe-associated intrinsically strong-binding sites, thereby passivating excessively strong H-adsorption centers.^56^ These findings further highlight the need to consider the role of oxygen species in future evaluations of catalytic performance under realistic operating conditions.^57^ Collectively, the combined evidence from *operando* XAS, DFT calculations, and electrochemical control experiments suggests that the quinary HEA system optimizes Δ*G*_H*_ through cooperative electronic redistribution across the multielement surface, thereby contributing to the enhanced catalytic performance observed in this work.

**Figure 9:**
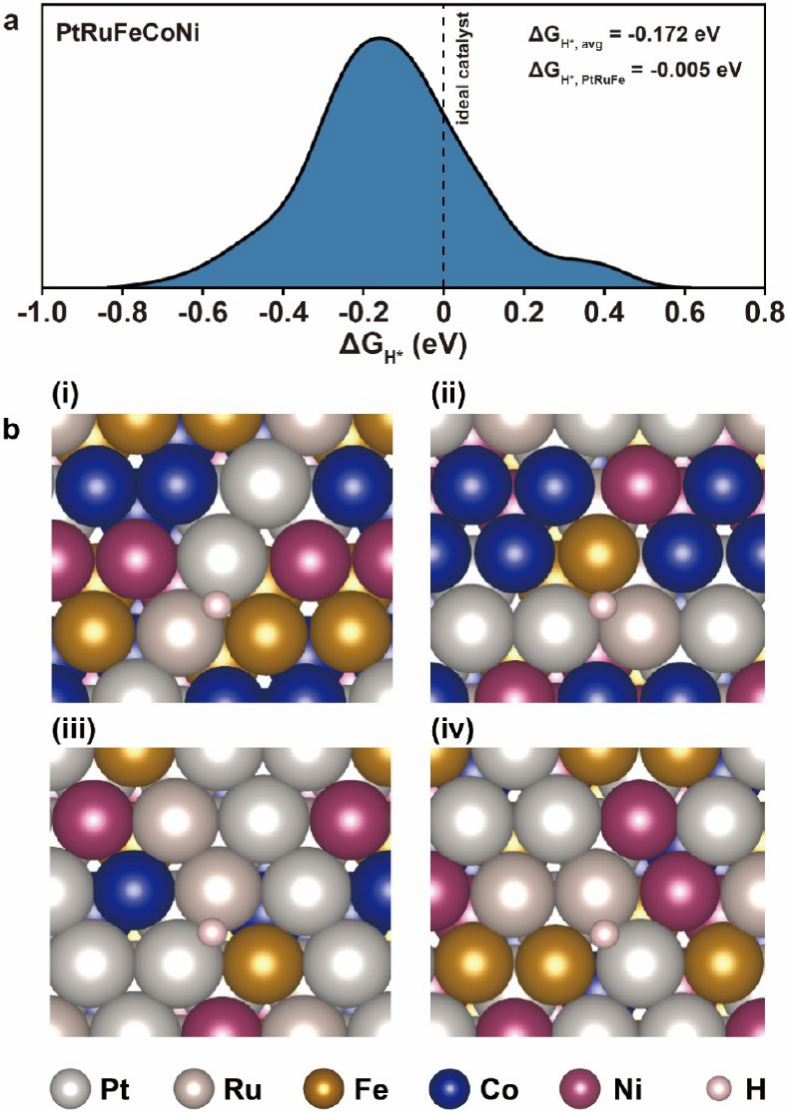
DFT calculations for hydrogen adsorption behaviors on PtRuFeCoNi HEA nanoparticles. (a) The distribution of hydrogen-adsorption free energy Δ*G*_H*_ for the PtRuFeCoNi HEA surface. (b) The four representative H-adsorption hollow sites between Pt, Ru, and Fe atoms on the PtRuFeCoNi HEA surface with optimal H-adsorption strength of ΔG_H_* = −0.005 eV for HER.

## Conclusion

We successfully developed a catalyst family comprising diverse 1 nm HEA nanoparticles with up to ten elements using the template-confined strategy. A key aspect of this strategy involves facilitating the autocatalytic reduction of the precursor mixture within mesoporous materials acting as nanoreactors under reducing conditions at low temperatures, as confirmed by H_2_-TPR experiments. Leveraging this family, we investigated quinary HEA nanoparticles with various metal combinations for HER and HOR, all constrained to a uniform size of approximately 1 nm. Among these, PtRuFeCoNi nanoparticles demonstrated notably improved catalytic activity. Furthermore, *operando* XAS analysis and DFT simulations suggest that the enhanced HER activity originates from cooperative electronic interactions among the constituent elements in the HEA nanoparticles. The Pt- and Ru-containing local environments provide favorable H-intermediate adsorption energetics, while the neighboring Fe, Co, and Ni atoms help modulate the local electronic structure through multielement coupling. These results highlight the potential of high-entropy alloying as a versatile strategy for designing ultrasmall nanoparticles with tunable composition, electronic structure, and catalytic performance.

## Experimental and Computational Details

### Template-Confined Synthesis of 1 nm PtRuFeCoNi Nanoparticles Using Mesoporous N-Doped CMK-3 as a Template

FeCl_3_·6H_2_O (0.001 mmol), CoCl_2_·6H_2_O (0.001 mmol), NiCl_2_·6H_2_O (0.001 mmol), RuCl_3_·xH_2_O (0.001 mmol), and H_2_PtCl_6_·6H_2_O (0.001 mmol) were dissolved in 16.7 μL ethanol. The vortex mixer was then used to obtain a 0.06 M homogeneous metal precursor solution. Subsequently, the mixture was dripped onto the CMK-3 at a rate of 0.9 mL/g for impregnation. The sample was then dried at 60 °C for 2 h and 90 °C for 1 h in an oven and transferred to a precleaned quartz boat through ultrasonication in DI water and ethanol for 15 min, respectively. The furnace was then purged with 10% H_2/_90% N_2_ at a rate of 40 mL/min for 30 min to remove air. The temperature was gradually increased to 150 °C for 30 min to ensure the evaporation of ethanol and 450 °C for 120 min for the chemical reduction. Finally, the furnace was naturally cooled to room temperature, and uniform-sized PtRuFeCoNi nanoparticles were obtained. The procedures for synthesizing other HEA nanoparticles with different compositions and loadings were similar to those of PtRuFeCoNi nanoparticles except for changing the type and concentration of metal precursors used.

### Computational Methods

#### Density Functional Theory Calculations

To simulate the PtRuFeCoNi surface, we constructed a 3 × 3, four-layer (111) slab. The lattice constant for this slab was determined by first optimizing the structures of Fe, Co, Ni, Pt, and Ru individually. Each metal’s lattice constant was converted into its corresponding metallic radius, and these radii were averaged to calculate the mean radius (*r*_ave_).^55^ This mean radius was then used to establish the final lattice constant, ensuring a balanced representation of the mixed-metal properties. For sampling H-adsorption configurations, our preliminary results indicated that H predominantly adsorbs on hollow sites. Consequently, we focused our sampling on hollow sites formed by all possible three-atom combinations (M_1_M_2_M_3_) within the PtRuFeCoNi alloy. We generated ten unique PtRuFeCoNi slabs, identifying 18 potential hollow H-adsorption sites on each PtRuFeCoNi(111) slab (Figure S53), yielding 180 possible positions. After geometry optimization, several configurations converged into the same adsorption state, reducing the total number of unique configurations to 154. Overall, our sampling comprehensively covers all possible hollow site combinations (Figure S54). The adsorption free energy of hydrogen (Δ*G*_H*_) is a crucial descriptor for assessing catalytic activity in HER. The closer Δ*G*_H*_ is to zero, the more favorable the reaction conditions. To estimate Δ*G*_H*_, we use the following equation:

$$\Delta G_{H*}=G\left(H^{*}\right)-G\left(*\right)-1/2G\left(H_{2}\right)\approx E\left(H^{*}\right)-E\left(*\right)-1/2E\left(H_{2}\right)+0.24\;(\mathrm{in eV})$$

Here, *E*(H*) and *E*(*) represent the electronic energies of [slab with hydrogen adsorbed] and [slab only], respectively. The value 0.24 eV accounts for zero-point energy corrections and entropy effects. This equation helps us gauge the HER performance of different materials, aiding in the design of more efficient catalysts.

All computations were carried out using density functional theory (DFT) with the Perdew–Burke–Ernzerhof (PBE) functional,^58,59^ as implemented in the Vienna Ab initio Simulation Package (VASP).^58^ The valence electron wave functions were expanded in a plane-wave basis set with a 390 eV cutoff,^60^ and the projector augmented wave (PAW) method was applied to account for core–electron interactions.^61^ A 7 × 7 × 1 k-point grid was sampled using the Monkhorst–Pack scheme for each calculation. All structures were fully optimized until the residual forces on each atom were below 0.02 eV/Å.

## Supplementary Material



## Notes

SEM, TEM images, Raman spectra, XRD, XPS, H_2_-TPR, TGA, DSC, XAS, electrocatalytic data, and DFT calculations (PDF)
