# Supplementary material for: Template-Confined Synthesis of 1 nm High-Entropy-Alloy Nanoparticle Library for Electrocatalysis
Source: ACS Nano. 2026 Jun 24;20(26):19037–55. doi: 10.1021/acsnano.6c06368 (PMC13348174; doi:10.1021/acsnano.6c06368)
Supplement: Supplementary file 1 [file nn6c06368_si_001.pdf]

Supporting information

Template-Confined Synthesis of 1-nm High-Entropy-Alloy Nanoparticle Library for Electrocatalysis

Chun-Wei Chang,[1,†] Yueh-Chun Hsiao,[1,2,†] Jui-Tai Lin,[1] Zong-Ying He,[6] Cheng-Kuang Lin,[1] Yi Chen,[1] Wen-Yang Huang,[3] Wei-Hsiang Huang,[4] Li-Yu Wang,[5] Han-Yuan Liu,[6] Yun-Shan Tsai,[1] Chia-Che Chang,[4] Wei-Chih Hsiao,[5] Wen-Jing Zeng,[3] Ying-Rui Lu,[4] Kun-Han Lin,[1] Sung-Fu Hung,[3] Chih-Wen Pao,[4] Chia-Min Yang,[5,6] Alexander J. Cowan,[2] and Tung-Han Yang[1,6,7,*]

[1]Department of Chemical Engineering, National Tsing Hua University, Hsinchu 300044, Taiwan.

[2]Stephenson Institute for Renewable Energy and Department of Chemistry, University of Liverpool, Liverpool L69 7ZF, United Kingdom.

[3]Department of Applied Chemistry and Center for Emergent Functional Matter Science, National Yang Ming Chiao Tung University, Hsinchu 300093, Taiwan.

[4]National Synchrotron Radiation Research Center, Hsinchu 300092, Taiwan.

[5]Department of Chemistry, National Tsing Hua University, Hsinchu 300044, Taiwan.

[6]College of Semiconductor Research, National Tsing Hua University, Hsinchu 300044, Taiwan.

[7]High Entropy Materials Center, National Tsing Hua University, Hsinchu 300044, Taiwan.

[§]Chun-Wei Chang and Yueh-Chun Hsiao contributed equally to this work.

*Corresponding author. Email: tunghanyang@mx.nthu.edu.tw

This PDF file includes:
Materials and Methods, Figures S1 to S48, Tables S1 to S14.

MATERIALS AND METHODS

Materials

All the chemicals used in the experiment are reagent grade. Pluronic triblock copolymer (P123), pyrrole (Ppy), iron(III) chloride ($FeCl_3$), iron(III) chloride anhydrous ($FeCl_3 \cdot 6H_2O$), cobalt(II) chloride hexahydrate ($CoCl_2 \cdot 6H_2O$), nickel(II) chloride hexahydrate ($NiCl_2 \cdot 6H_2O$), molybdenum(V) chloride ($MoCl_5$), copper(II) chloride ($CuCl_2$), zinc chloride ($ZnCl_2$), ruthenium chloride hydrate ($RuCl3 \cdot xH_2O$), rhodium chloride hydrate ($RhCl_3 \cdot xH_2O$), chloroplatinic acid hexahydrate ($H_2PtCl_6 \cdot 6H_2O$), sodium tetrachloropalladate (Na_2PdCl_4), hydrochloric acid (HCl, 37%), 5wt% Nafion solution, and sulfuric acid (H_2SO_4, 95-97%) were all obtained from Sigma-Aldrich. Tetraethoxysilane (TEOS, 98%) and zirconyl chloride octahydrate ($ZrOCl_2 \cdot 8H_2O$) were obtained from Acros. Hydrofluoric acid (HF) was obtained from Union Chemical. Ethanol (C_2H_5OH, 99.5%) was purchased from J. T. Baker. Commercial 20% Pt nanoparticles dispersed on Vulcan XC-72 with a particle size range of 2-3 nm were purchased from Premetek Company. Deionized (DI) water with a resistivity of 18.2 MΩ cm was used for all the experiments.

Synthesis of short-channel mesoporous silica SBA-15

The mesoporous SBA-15 was synthesized by adding TEOS into a DI water solution containing a mixture of P123, HCl, and $ZrOCl_2$. The solution was continuously stirred at 35 °C overnight to dissolve the mixture. Then, TEOS was slowly injected, stood for 24 hours at 35 °C, and aged at 90 °C for 24 hours. After cooling to room temperature, 23.2 g of H_2SO_4 was added and again heated to 90 °C for 24 hours with stirring. The resultant solid was filtered, washed with a large amount of water, dried, and then calcined at 300 °C to derive the mesoporous silica. Note that the molar ratio of TEOS:H_2O:P123:HCl:$ZrOCl_2$ = 1:193:0.017:5.9:0.05.

Synthesis of N-doped CMK-3

The preparation of N-doped carbon using polypyrrole as carbon and nitrogen source. 1g of short-channel SBA-15 was first impregnated with 0.8 g of $FeCl_3$ (oxidizing agent) dissolved in 1.5 mL ethanol and then dried at 50 °C with a vacuum suction system for 3 hours. The oxidizing agent-loaded sample was put under the saturated-pyrrole vapor at 35 °C overnight. After polymerization, the black powder was then collected and carbonized at 850 °C under nitrogen flow for 4 hours with a rate of 5 °C/min. The silica template and iron species were removed after washing with hydrofluoric (HF) acid several times at room temperature. The obtained N-doped carbon product was collected and filtered, washed with a large amount of ethanol and water, and dried at 90 °C.

Material characterizations

We collected the X-ray diffraction (XRD) patterns using an X-ray diffractometer (Bruker, D8A25) using a copper target. We performed the scanning electron microscopy (SEM) images using a field emission scanning electron microscope (FE-SEM) operated at 30 kV (Hitachi, SU8010). We studied the transmission electron microscopy (TEM) and high-angle annular dark-field scanning electron microscopy (HAADF-STEM) images and energy-dispersive spectroscopy (EDS) mapping using spherical-aberration corrected field emission TEM (JEOL, JEM-ARM200FTH) operated at 200 kV. For the EDS mappings of a single HEA nanoparticle along with the corresponding spectrum (Figures 2m, n, and S9), we used a spherical-aberration corrected field emission transmission electron microscope (Thermo Fisher Spectra 300) operating at 300 kV. We analyzed the element contents using an elemental analyzer (EA) (Elementar, vario EL cube for NCSH) and an inductively coupled plasma optical emission spectrometer (ICP-OES) (Thermo Scientific, iCAP 7200 Duo). We used confocal micro-Raman spectroscopy (Horiba Jobin Yvon, LabRAM HR 800 UV) to record the Raman spectra. We measured the X-ray photoelectron spectroscopy (XPS) spectra using a high-resolution X-ray photoelectron spectrometer (HRXPS) (ULVAC-PHI, PHI Quantera II) with a probing depth of approximately 5 nm. We performed the temperature-programmed reaction (TPR) using a

chemisorption analyzer (Micromeritics, Autochem II 2920). We measured the BET specific surface area using a porosimetry analyzer (Micromeritics, Tristar II Plus). X-ray photoelectron spectroscopy (XPS) measurements were carried out to analyze the surface chemical composition of the samples. The XPS spectra were obtained using a high-resolution X-ray photoelectron spectrometer (HRXPS) (ULVAC-PHI, PHI Quantera II) with X-ray microprobe with 7.5 μm spatial resolution, 20 kV Ar Gas cluster ion beam gun and Al monochromator (1486.6 eV) X-ray source. The binding energies of the spectra were calibrated using the C 1s peak at 284.8 eV. The XPS fittings were carried out by using the software XPSPEAK41, and the Shirley background function was used to fit the spectra.

Wide angle X-ray scattering analysis

The crystal structures of PtRuFeCoNi HEA nanoparticles were determined at Beamline 17A1 of the National Synchrotron Radiation Research Center (NSRRC), Taiwan. X-ray diffraction experiments were performed using an X-ray source with an energy of 9 keV. A triangular bent Si (111) single crystal was utilized to generate a monochromatic beam with a wavelength of λ=1.320545 Å. The diffraction patterns were recorded using image plates (Fuji BAS III) with an area of 20x40 cm^2 and a pixel resolution of 100 μm. The sample-to-detector distance during data acquisition was set at 167.7 mm.

Temperature programmed reduction of hydrogen (H_2-TPR)

The TPR measurements were performed with the chemisorption analyzer (Micromeritics, Autochem II 2920). The samples (0.1 g) were loaded in a quartz boat and 10% H_2/90% N_2 was purged at the rate of 40 mL/min until the baseline on the recorder remained unchanged. The samples were heated from 50 to 600 °C under the mixture gas flow (10% H_2/90% N_2). When the temperature was raised to 150 °C and 450 °C, the temperature would be held for 30 minutes and 120 minutes, respectively, which is at the same conditions as the HEA nanoparticles synthesized. H_2 consumption was measured using an

online thermal conductivity detector (TCD).

X-ray absorption spectroscopy (XAS)

The XAS measurements were conducted using a three-electrode setup in a Teflon container equipped with a Kepton-sealed window. The experimental conditions were identical to those used for the HER measurements mentioned above. The XAS signals were obtained in total-fluorescence-yield mode at the National Synchrotron Radiation Research Center (NSRRC), Taiwan. The *ex situ* XAS spectra were recorded at beamline TPS 44A of NSRRC (Figure 4). For *operando* XAS measurements of PtRuFeCoNi HEA nanoparticles (Figure 8), Ru and Fe were analyzed at beamline TLS01C1 of NSRRC, while Pt, Co, and Ni were analyzed at beamline TLS17C1 of NSRRC. The scan ranges were set to 11410-12140 eV for Pt L_3-edge, 21865-22765 eV for Ru K-edge, 6930-7630 eV for Fe K-edge, 7525-8285 eV for Co K-edge, and 8165-8860 eV for Ni K-edge. Each element was measured separately. The spectra were obtained by subtracting the pre-edge baseline and normalizing the post-edge. The beam pattern was optimized before operando XAS, with metal foil references used for energy calibration. Each measurement took approximately 40 minutes. To enhance signal quality, *operando* XAS was conducted at various applied voltages (OCP, 0.05 V, -0.05 V, and -0.15 V) and repeated 3-5 times, with the results averaged to further improve signal clarity and reliability. The XANES and EXAFS data, along with the corresponding post-edge background subtraction and normalization, were analyzed using the widely used “Athena software”. EXAFS analysis involved Fourier transform of k^2-weighted EXAFS oscillations. EXAFS fitting was performed using the IFFEFIT software package. Using Fe as an example, the theoretical scattering paths were generated by first importing the Fe FCC crystal structure from the ICSD database into Artemis. Following the initial atomic potential calculation (Run Atoms), the first coordination shell was manually modified to approximate the nominal equiatomic local environment while allowing for finite-size compositional fluctuations. Specifically, the 12 nearest-neighbor sites in the Fe-centered first shell were initialized as

3 Pt, 3 Ru, and 6 3d neighbors (representing Fe, Co, and Ni) to serve as the starting configuration. Because the 3d elements (Fe, Co, and Ni) exhibit highly similar EXAFS backscattering functions and strong parameter correlations, we grouped their contributions into a single Fe-3d term, where Fe-3d denotes coordination with 3d neighbors. The FEFF6 code was then executed to calculate the theoretical scattering paths used for the subsequent structural refinement (Run Feff).

Electrocatalytic HER and HOR

Electrochemical measurements (Nova system) were conducted using glassy carbon (GC). Pt disk as the counter electrode and Ag/AgCl was used as a reference electrode for hydrogen evolution reaction (HEA) while Hg/HgO was used for hydrogen oxidation reaction (HOR). GC electrode is carefully polished before dispersing the catalytic ink every time. The electrocatalytic ink was made of 1 mg HEA/C dispersed in 500 μL DI water, 500 μL isopropanol, and 20 μL 5%Nafion. The mixture was then transferred to an ultrasonicator in the ice bath. After 30 minutes of sonicating, the well-mixed electrocatalytic ink was formed. The 3.6 μL homogeneous solution was pipped on the GC electrode and dried at isopropanol-saturated atmosphere overnight. The electrochemical measurements for HER were performed in 0.5 M sulfuric acid and N_2-saturated atmosphere. The potential of working electrode was cycled several times and then maintained under -5 mV_{RHE} for 120 seconds to remove the contaminants and oxidants on the surface. After the pretreatments, the CV was recorded between 0.05 and 1.1 V RHE with a scan rate of 50 mV/s. The LSV curve was recorded from 0.1 to -0.23 V_{RHE} under 1600 rpm with a scan rate of 1 mV/s and 80% IR compensation. For HOR electrocatalytic measurements, the electrolyte was first purged with N_2 gas for a duration of 30 minutes. To clean the electrode surface, 50 cycles of CV testing are conducted, with the potential range spanning from 0.05 to 1.05 V_{RHE}, and employing a scan rate of 500 mV s^{-1}. After that, catalytic measurements are performed within a 0.1 M KOH electrolyte, which had been purged independently with N_2 and H_2 gases.CV curves are recorded from 0.05 to 1.05 V_{RHE} at a scan rate of 10 mV s^{-1}. LSV curves are

systematically recorded over a potential range spanning from -0.05 V to 0.7 V_{RHE} under a rotational speed of 1600 rpm at the scan rate of 5 mV s^{-1}, respectively. Table S11 provides a summary of the electrochemical protocol used for the electrocatalytic HER and HOR measurements, outlining the key conditions and steps involved in the experiments.

Structural models of 55-atom clusters

The catalyst models were constructed as 55-atom icosahedral nanoparticles, representing a characteristic size for sub-nanometer clusters. To systematically investigate the influence of local chemical environments on catalytic activity, the surface atoms of the cluster were categorized into three distinct types of active sites based on their coordination numbers and geometric positions: vertex, edge, and terrace sites. The vertex sites consist of atoms at the twelve corners of the icosahedron, characterized by the lowest coordination number. The edge sites are located along the boundaries connecting the vertices, while the terrace sites correspond to the atoms situated on the (111)-like triangular facets of the cluster surface. This classification allows for a high-throughput analysis of site-specific adsorption behaviors across the complex configurational space of the HEA systems. Hydrogen adsorption was evaluated at various positions, including top sites on vertex atoms, bridge sites across edges, and hollow sites on the terraces, to identify the most energetically favorable configurations for the HER.

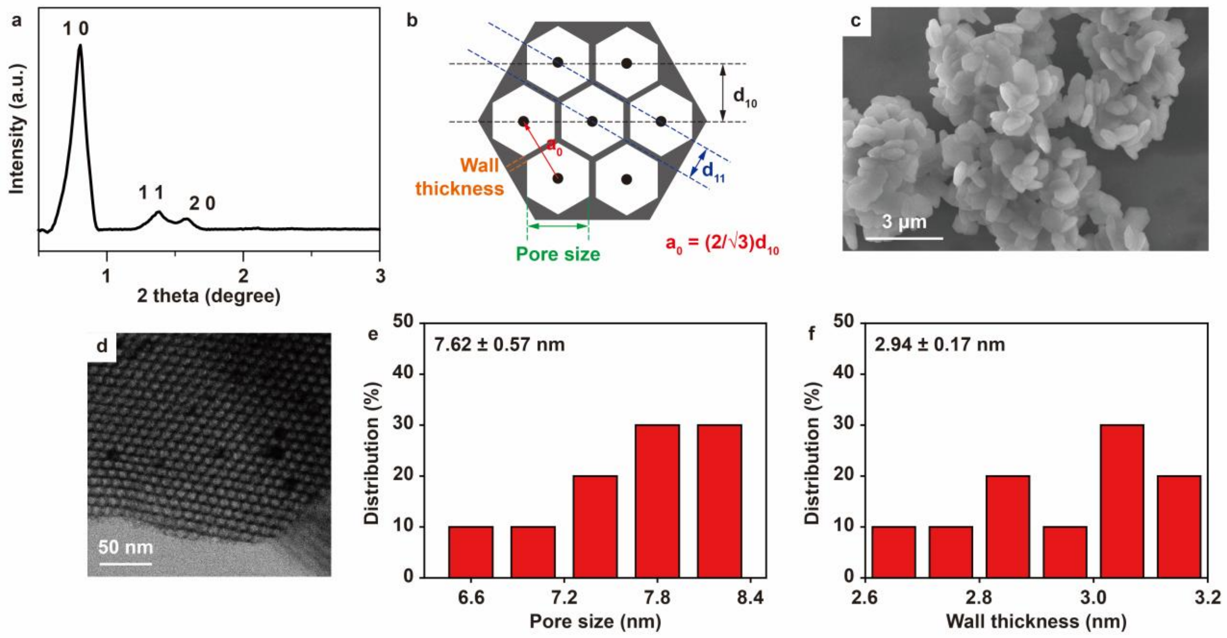

Figure S1. Characterizations of short-channel mesoporous silica SBA-15. (a) Small-angle XRD pattern, (b) schematic representation of the pore structure, (c) SEM image (d) HR-TEM image, (e) pore size distribution, and (f) wall thickness distribution. Notably, (e) and (f) are derived from the HR-TEM image in (d). Small-angle XRD patterns confirmed a 2D hexagonal MCM-41 type lattice structure, with observed 1 0, 1 1, and 2 0 reflection peaks. The interplanar spacing (d_{10}) was calculated as 10.80 nm, and the lattice parameter (a_0) was determined to be 12.40 nm ($a_0=\frac{2}{\sqrt{3}}d_{10}$), which can be used to estimate the wall thickness (Figure S1a and b). SEM analysis revealed a plate thickness of ~170 nm with uniform morphology and size distribution (Figure S1c). HR-TEM images along the pore axis of SBA-15 (Figure S1d) reveal that the bright areas correspond to the cylindrical pores, while the dark regions represent the silica walls. These results demonstrate that the cylindrical pores are parallel to the short side of the plates, exhibiting high pore regularity and a well-ordered hexagonal geometry. This observation is consistent with the XRD results, indicating that the pore size is approximately 7.62 nm (Figure S1e) and the wall thickness is around 2.94 nm (Figure S1f).

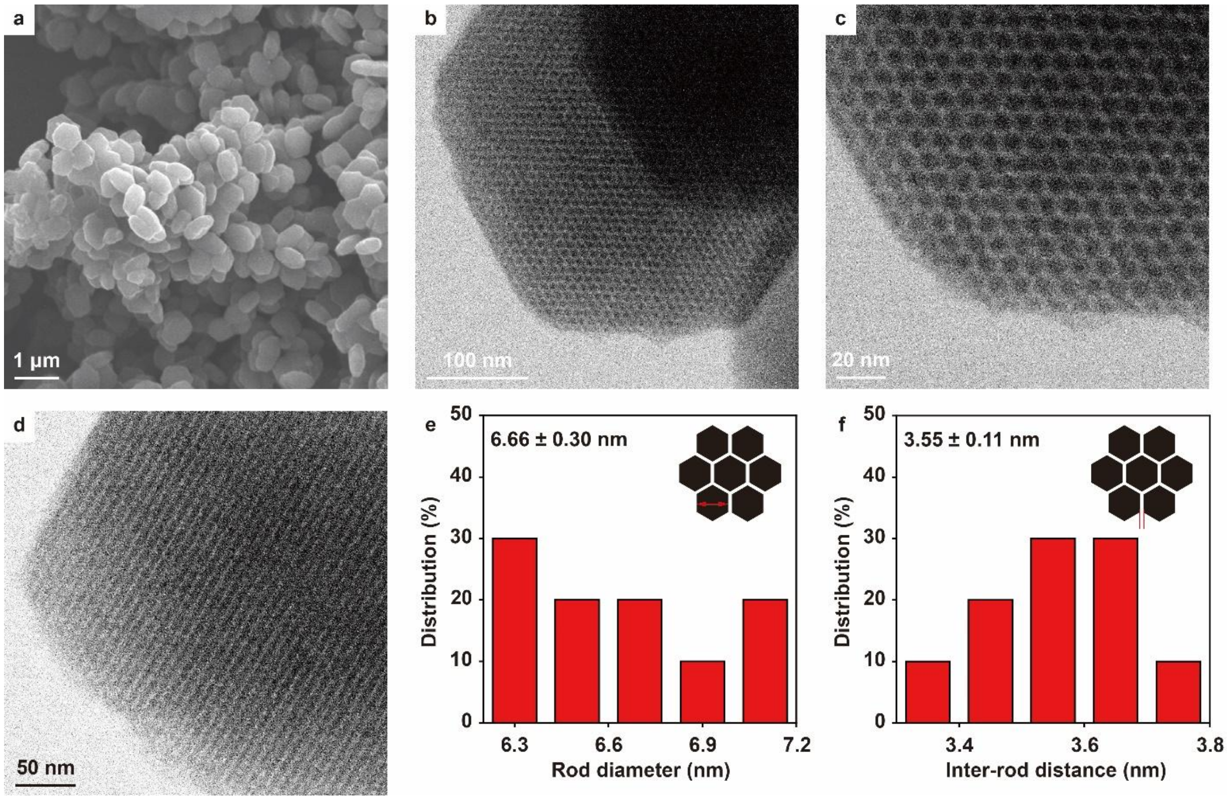

Figure S2. Structural characterization of N-doped CMK-3. (a) SEM image, (b-d) HR-TEM images of CMK-3 along (b, c) and perpendicular (d) to the hexagonal pore alignment direction, and (e) rod diameter and (f) inter-rod distance distributions derived from the TEM image in (c). The SEM images with lower magnification suggest that the CMK-3 plates possess high uniformity and structural integrity, corroborating the reproducibility of the hard-template synthesis method (Figure S2a). The HR-TEM images along (Figure S2b and c) and perpendicular (Figure S2d) to the direction of pore alignment confirm that the carbon rods (appearing as black regions) and the cylindrical pores (white spaces between the rods) are parallel to the short side of the hexagonal plates, in reverse arrangement compared to the SBA-15 template. The rod diameter was observed to be approximately 6.66 nm (Figure S2e), 12.60% smaller than the original SBA-15 pore size (7.62 nm), and the space between the carbon rods was determined to be 3.55 nm (Figure S2f).

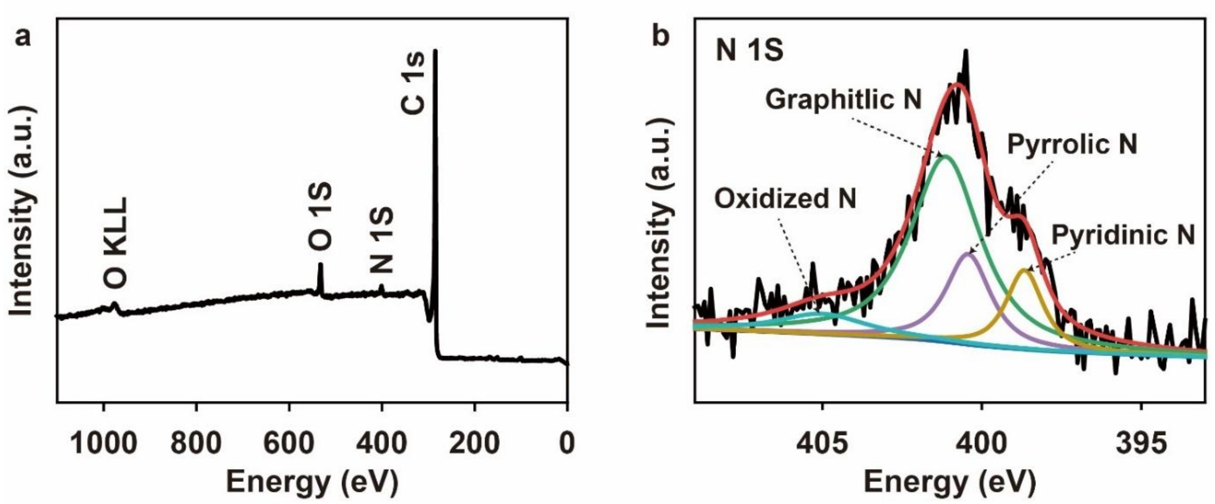

Figure S3. The XPS spectra of N-doped CMK-3. (a) Total survey spectrum and (b) N 1s spectrum. The N 1s group can be deconvoluted into four parts, suggesting that graphitic N constitutes the majority, followed by pyridinic N, pyrrolic N, and oxidized N (Table S4).

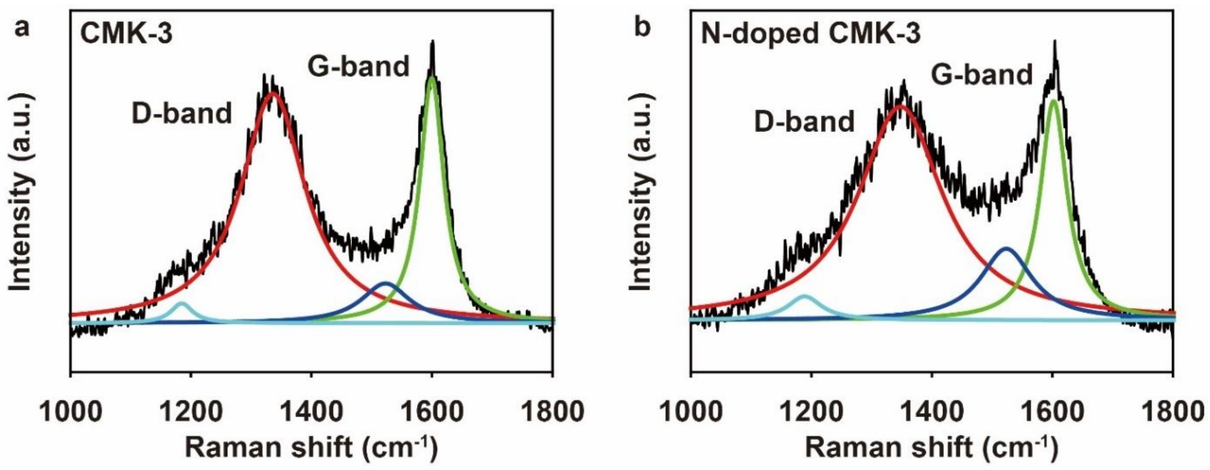

Figure S4. The Raman spectra of CMK-3 with and without nitrogen doping. (a) CMK-3. (b) N-doped CMK-3. We performed fitting with a 5-band model following well-established procedures commonly employed in the characterization of carbon materials. The curve fitting was carried out using OriginPro 2021 software, in accordance with the Raman spectra analysis protocol outlined in the literature.[1, 2] Specifically, the fitting process involved the following steps: (1) Selecting the entire relevant peaks along with a baseline region as the fitting window; (2) Fitting the baseline using a linear or polynomial function, depending on which yielded the most accurate results; (3) Decomposing the Raman bands in the first-order region of the spectrum using an appropriate fitting function (e.g., Lorwntzian) with the initial peak positions assigned as G = 1580 cm^{-1}, D_1 = 1350 cm^{-1}, D_2 = 1620 cm^{-1}, D_3 = 1500 cm^{-1}, D_4 = 1200 cm;[1, 3, 4] (4) Iteratively adjusting the fitting, including adding hidden peaks, removing redundant peaks, and optimizing peak parameters, until the residuals were minimized and the R^2 of the fit indicated no further improvement. During the fitting optimization process in our case, we observed the disappearance of the D_2 peak, which is typically seen as a shoulder on the G band and reflects disorder in the graphitic lattice (surface graphene layers, E_{2g}-symmetry). This suppression of the D_2 band may be attributed to the presence of dopants or adsorbates in the mesoporous carbon materials, which modify the electronic structure and reduce the Raman response, a phenomenon commonly reported in the literature.

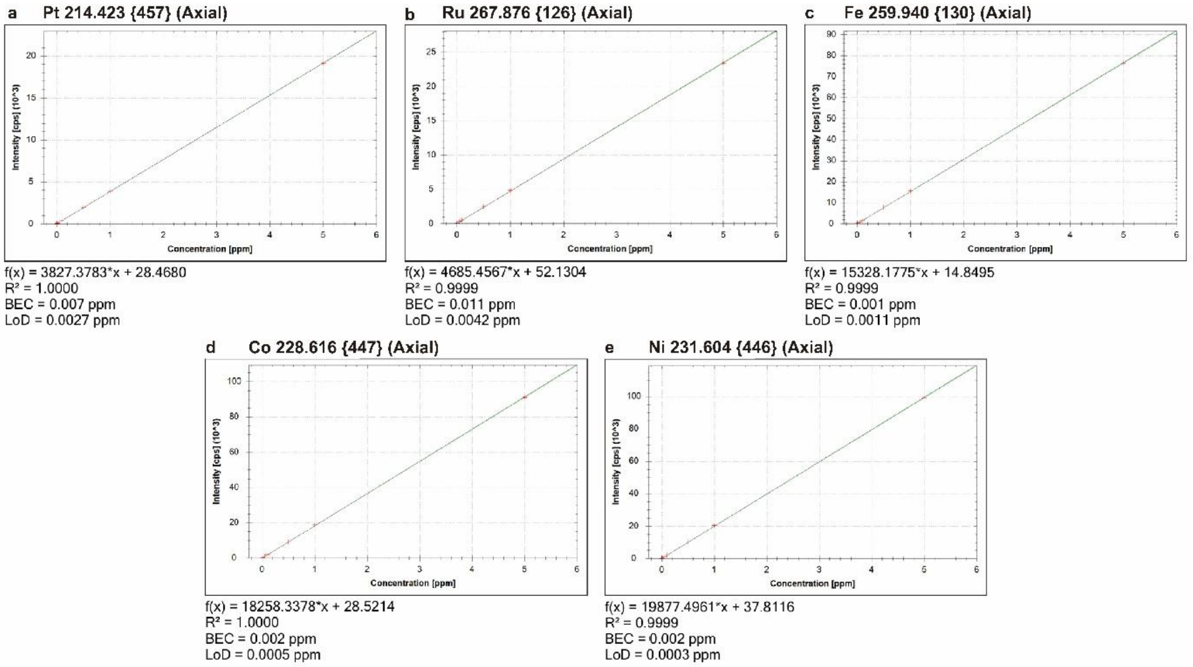

Figure S5. Calibration curves of (a) Pt, (b) Ru, (c) Fe, (d) Co, and (e) Ni obtained by ICP-OES. Standard solutions with concentrations of 0.05, 0.1, 0.5, 1, and 5 ppm were used to construct the calibration curves, yielding correlation coefficients (R^2) close to 1. To minimize spectral interference among the five elements, the analytical wavelengths were selected as follows: Pt (214.423 nm), Ru (267.876 nm), Fe (259.940 nm), Co (228.616 nm), and Ni (231.604 nm).

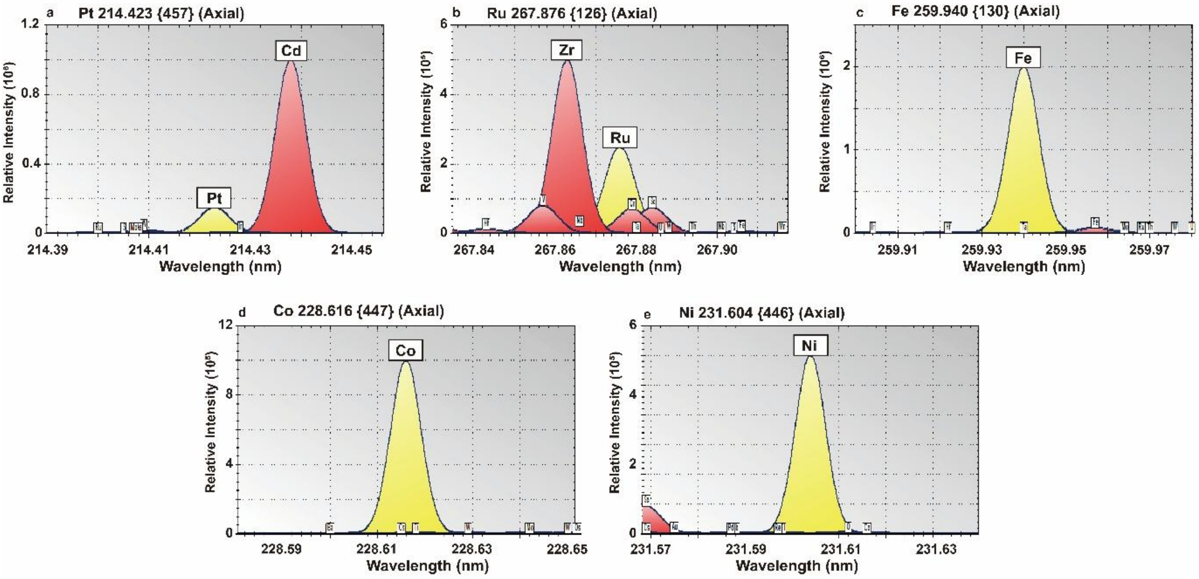

Figure S6. ICP-OES spectra of (a) Pt (214.423 nm, a range of 214.416 to 214.430), (b) Ru (267.876 nm, a range of 267.866 to 267.886), (c) Fe (259.940 nm, a range of 259.928 to 259.952), (d) Co (228.616 nm, a range of 228.604 to 228.628), and (e) Ni (231.604 nm, a range of 231.594 to 231.614). The yellow peaks indicate the characteristic emission lines of each element, which are well separated without any overlap, confirming that no spectral interference occurs among the five elements.

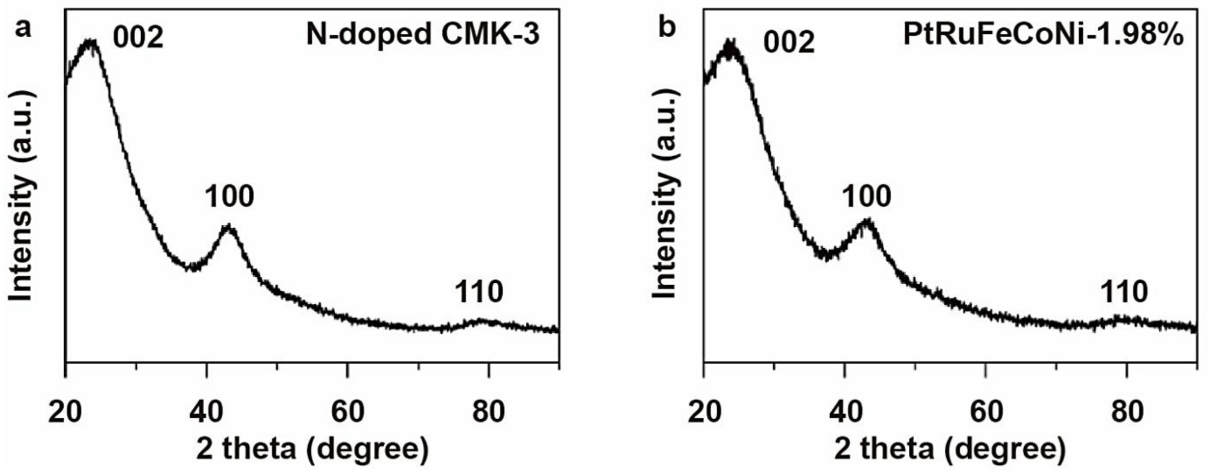

Figure S7. XRD patterns of (a) N-doped CMK-3 and (b) 1-nm PtRuFeCoNi HEA nanoparticles supported on N-doped CMK-3, collected using a Bruker D8A25 XRD diffractometer with a Cu target. The N-doped CMK-3 support itself exhibits broad maxima at $2\theta \approx 23°$, 43°, and 80°, which are characteristic of turbostratic or disordered graphitic carbon and are commonly associated with the 002, 100, and 110 reflections. Importantly, the PtRuFeCoNi/N-doped CMK-3 composite displays an essentially identical diffraction profile without additional peaks assignable to an FCC metallic phase, which is attributed to the limited long-range atomic ordering arising from the ultrasmall particle size of ~1 nm.

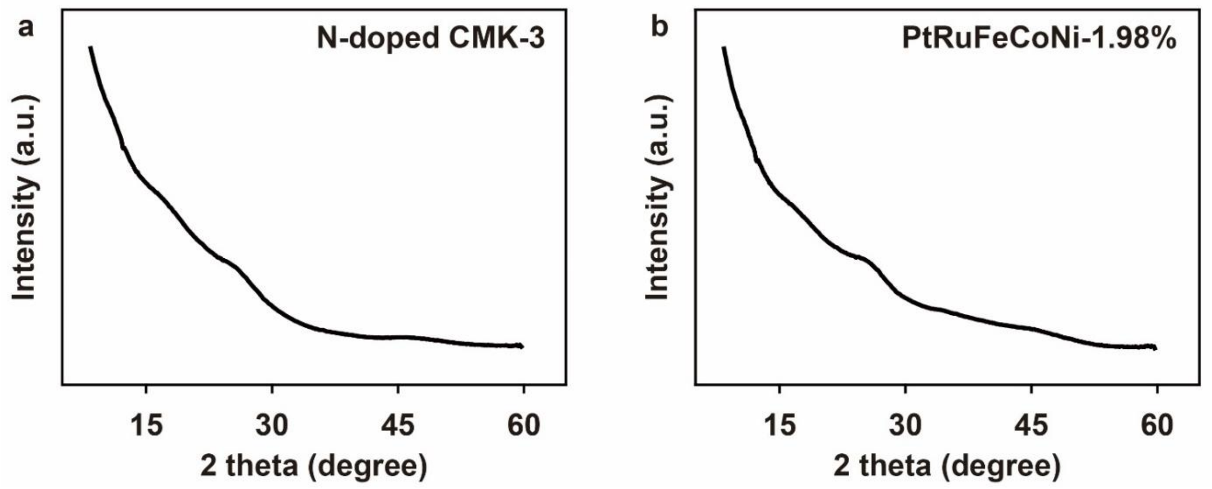

Figure S8. The high-resolution synchrotron powder X-ray diffraction (HRPXRD) patterns. (a) N-doped CMK-3 and (b) 1-nm PtRuFeCoNi HEA nanoparticles supported on N-doped CMK-3. The diffraction patterns do not exhibit noticeable differences between (a) and (b) due to the ultrasmall particle size of 1-nm PtRuFeCoNi nanoparticles.

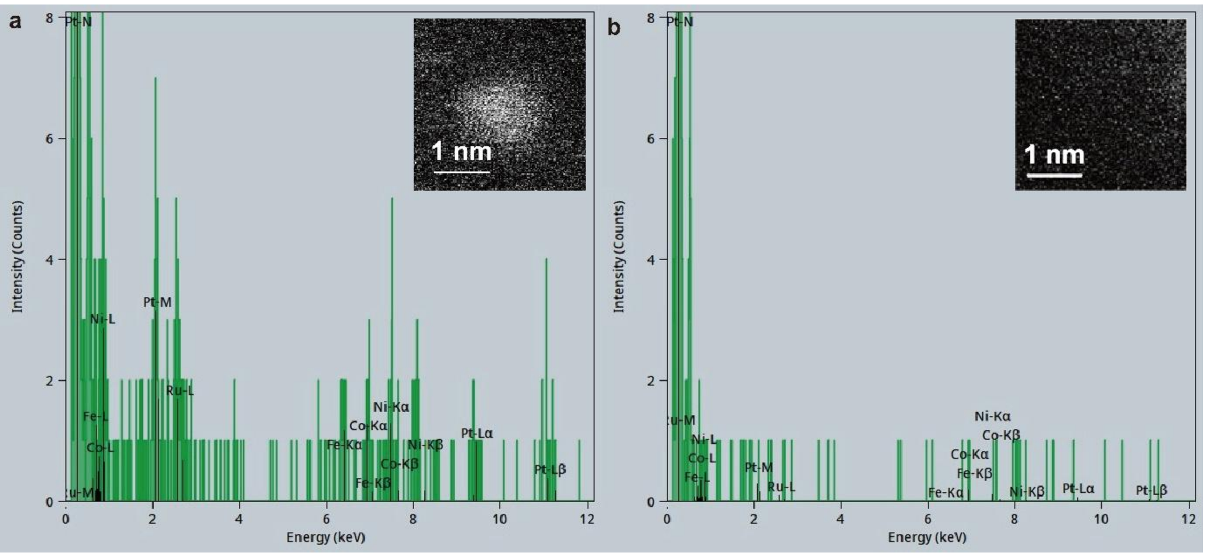

Figure S9. The EDS spectra of (a) individual 1-nm PtRuFeCoNi HEA nanoparticle and (b) N-doped CMK-3. Compared to the spectrum of N-doped CMK-3, the 1-nm HEA nanoparticles exhibit distinct signals for Pt, Ru, Fe, Co, and Ni elements. The characteristic lines and their nominal energies used for mapping were Pt-Lα (≈ 9.44 keV), Ru-Lα (≈ 2.56 keV), Fe-Kα (≈ 6.40 keV), Co-Kα (≈ 6.93 keV), and Ni-Kα (≈ 7.48 keV) (Figure S9a). In spectrum imaging, each elemental map was generated from the background corrected integrated counts within the predefined energy window around the selected line, rather than from a single channel peak height. This integrated intensity approach is more robust against counting noise and is the standard practice for EDS spectrum imaging. We explicitly evaluated possible interferences in two ways. First, we inspected the raw spectra extracted from the nanoparticle region and examined expanded spectral windows around each analytical line. Second, we selected analytical lines that minimize cross talk and avoid common overlaps from neighboring transitions. In particular, for the 3d metals, Fe-Kβ (≈ 7.06 keV) is adjacent to Co-Kα (≈ 6.93 keV), and Co-Kβ (≈ 7.65 keV) lies near Ni-Kα (≈ 7.48 keV). To avoid ambiguity, we primarily used the Kα lines of Fe, Co, and Ni because they are better separated within the 6-8 keV region. The spectral separation among Fe Kα (6.40 keV), Co Kα (6.93 keV), and Ni Kα (7.48 keV) enables reliable assignment under our acquisition conditions. We also collected spectra from regions of bare N-doped CMK-3 without nanoparticles (Figure S9b). The support only region exhibits only low energy signals associated with C, N, and background from the grid, whereas the nanoparticle containing region shows clear metal related features at the expected energies. This comparison confirms that the metal signals originate from the PtRuFeCoNi nanoparticles rather than the support. Because EDS intensities are strongly influenced by specimen thickness, absorption, detector geometry, and local mass thickness, we do not use EDS maps or EDS derived intensities for quantitative composition determination. Instead, the quantitative bulk elemental ratios reported in Figure 2l were determined by ICP-OES, while EDS mapping and line scan analyses (Figure 2m, 2p, and 2r) serve as complementary qualitative evidence for homogeneous elemental distribution.

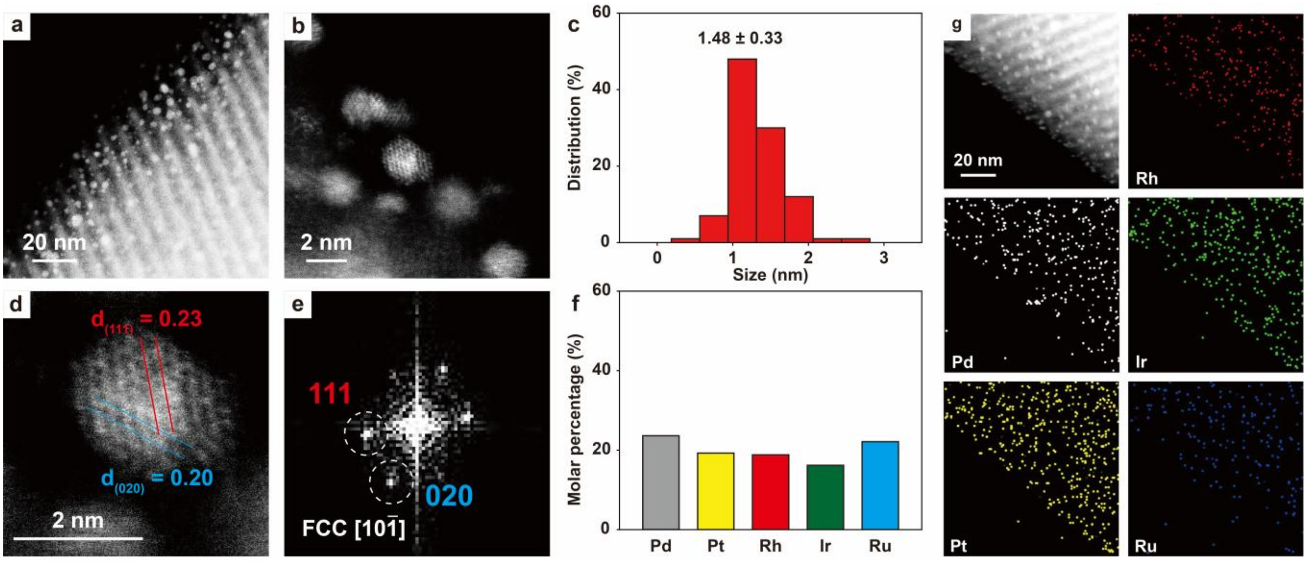

Figure S10. Structural and compositional characterizations of 1-nm PdPtRhIrRu HEA nanoparticles. (a, b) Low-magnification HAADF-STEM images showing uniform dispersion of nanoparticles. (c) Particle size distribution with an average diameter of 1.48 ± 0.33 nm. (d) Enlarged image of an individual nanoparticle and (e) the corresponding FFT pattern, where the lattice spacings of 0.23 and 0.20 nm can be indexed to the (111) and (200) planes, respectively, of the FCC structure. (f) ICP-OES analysis confirming a near-equiatomic elemental composition of Pd, Pt, Rh, Ir, and Ru. (g) EDS elemental mapping images of PdPtRhIrRu HEA nanoparticles. The HAADF-STEM image and the corresponding elemental maps of Pd, Pt, Rh, Ir, and Ru demonstrate uniform and overlapping spatial distributions of all five elements within N-doped CMK-3.

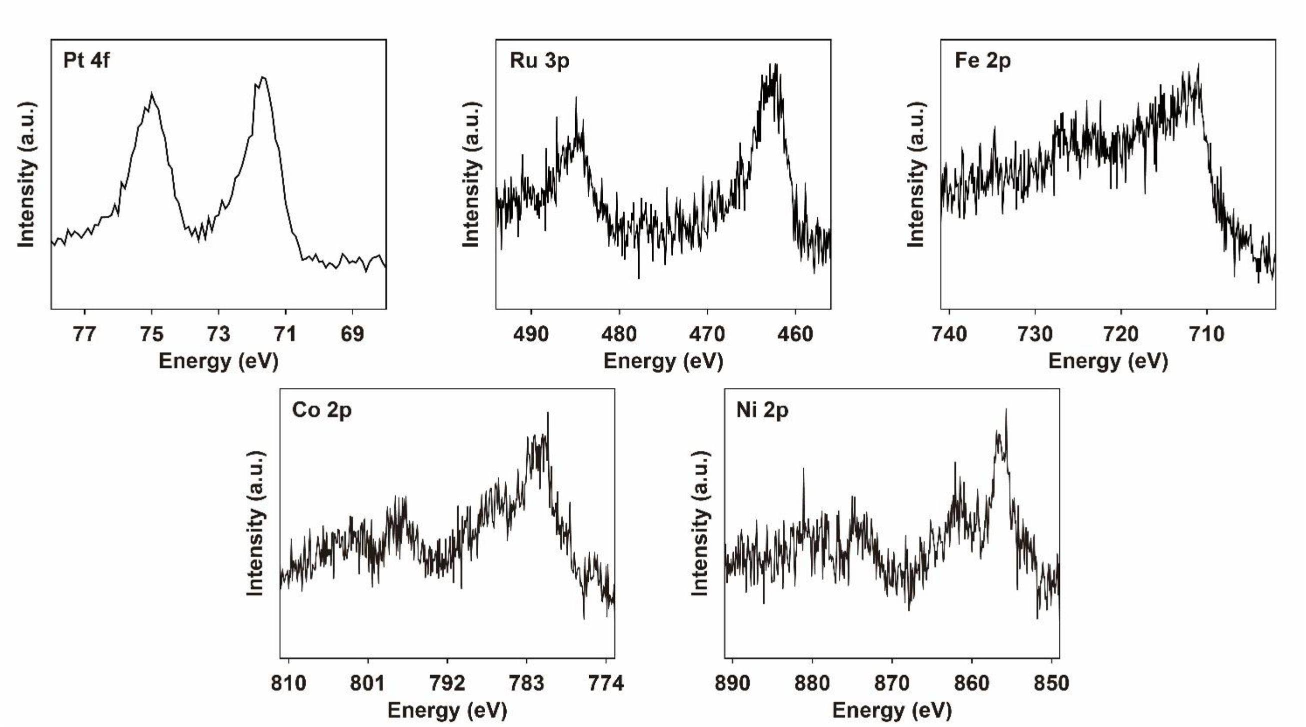

Figure S11. Raw, unsmoothed XPS spectra of PtRuFeCoNi HEA nanoparticles in the Pt 4f, Ru 3p, Fe 2p, Co 2p, and Ni 2p regions.

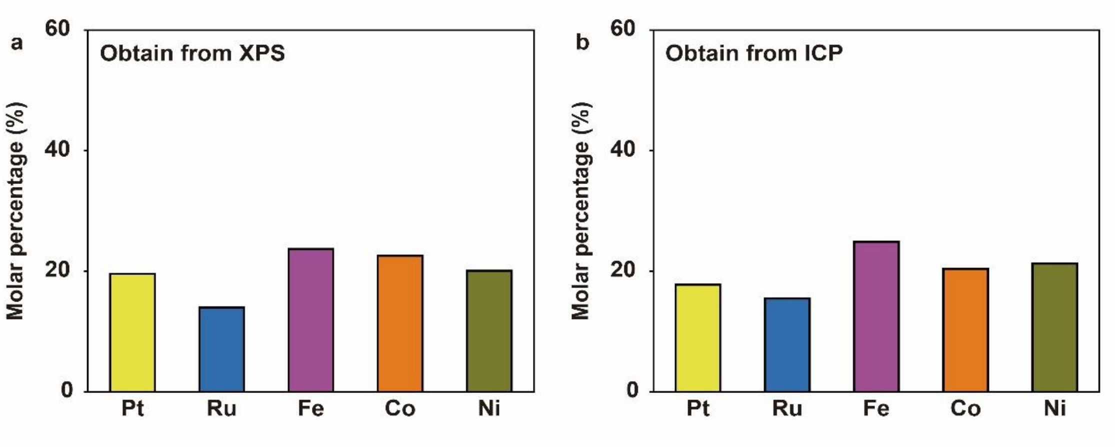

Figure S12. Molar composition of PtRuFeCoNi HEA nanoparticles determined by (a) XPS and (b) ICP-OES analyses. The atomic percentages of Pt/Ru/Fe/Co/Ni obtained from XPS are 19.6/14.0/23.7/22.6/20.1, respectively, while those measured by ICP-OES are 17.8/15.6/24.9/20.4/21.3. The overall compositions derived from the two techniques are in good agreement, with only minor deviations attributable to their different probing depths and measurement principles.

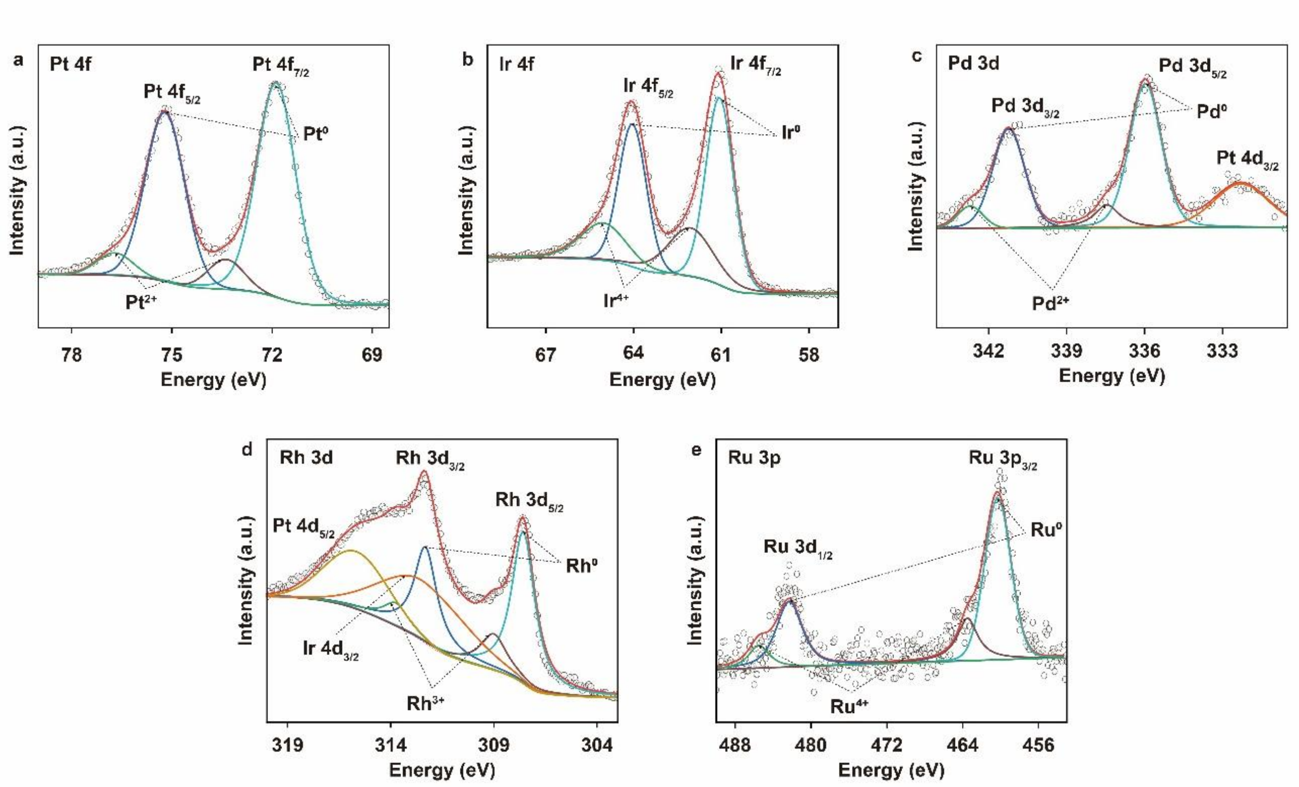

Figure S13. Surface chemical states of PdPtRhIrRu HEA nanoparticles characterized by XPS. High-resolution XPS spectra of (a) Pt 4f, (b) Ir 4f, (c) Pd 3d with overlapping Pt 4d, (d) Rh 3d with overlapping Pt 4d and Ir 4d, and (e) Ru 3p. All spectra are dominated by metallic components with only minor contributions from oxidized species, indicating a largely metallic surface for the PdPtRhIrRu HEA nanoparticles. Quantitative peak deconvolution reveals high metallic fractions of 91.21% (Pd), 89.32% (Pt), 78.08% (Rh), 67.11% (Ir), and 77.05% (Ru), confirming the formation of predominantly metallic bonding and demonstrating the strong oxidation resistance of this PGM-based HEA system. This result contrasts with the known susceptibility of 3d transition metals (e.g., Fe, Co, and Ni) to surface oxidation under electrochemical conditions, supporting the superior surface stability of the noble-metal HEA nanoparticles.

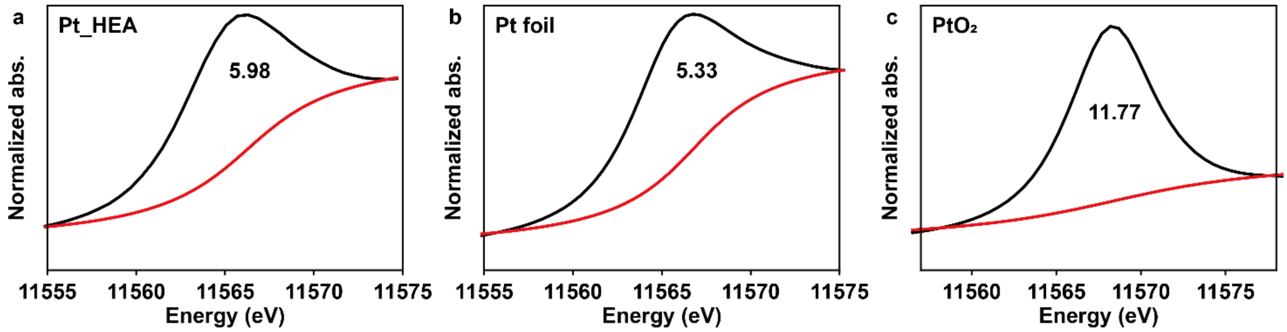

Figure S14. White-line peak areas of Pt L_3-edge for 1-nm PtRuFeCoNi HEA nanoparticles, metallic foils, and oxides. The peak area was extracted by subtracting the arctangent function background. The center of the arctangent background was set at the white-line peak position, using the "peak fitting" function in "Athena software".

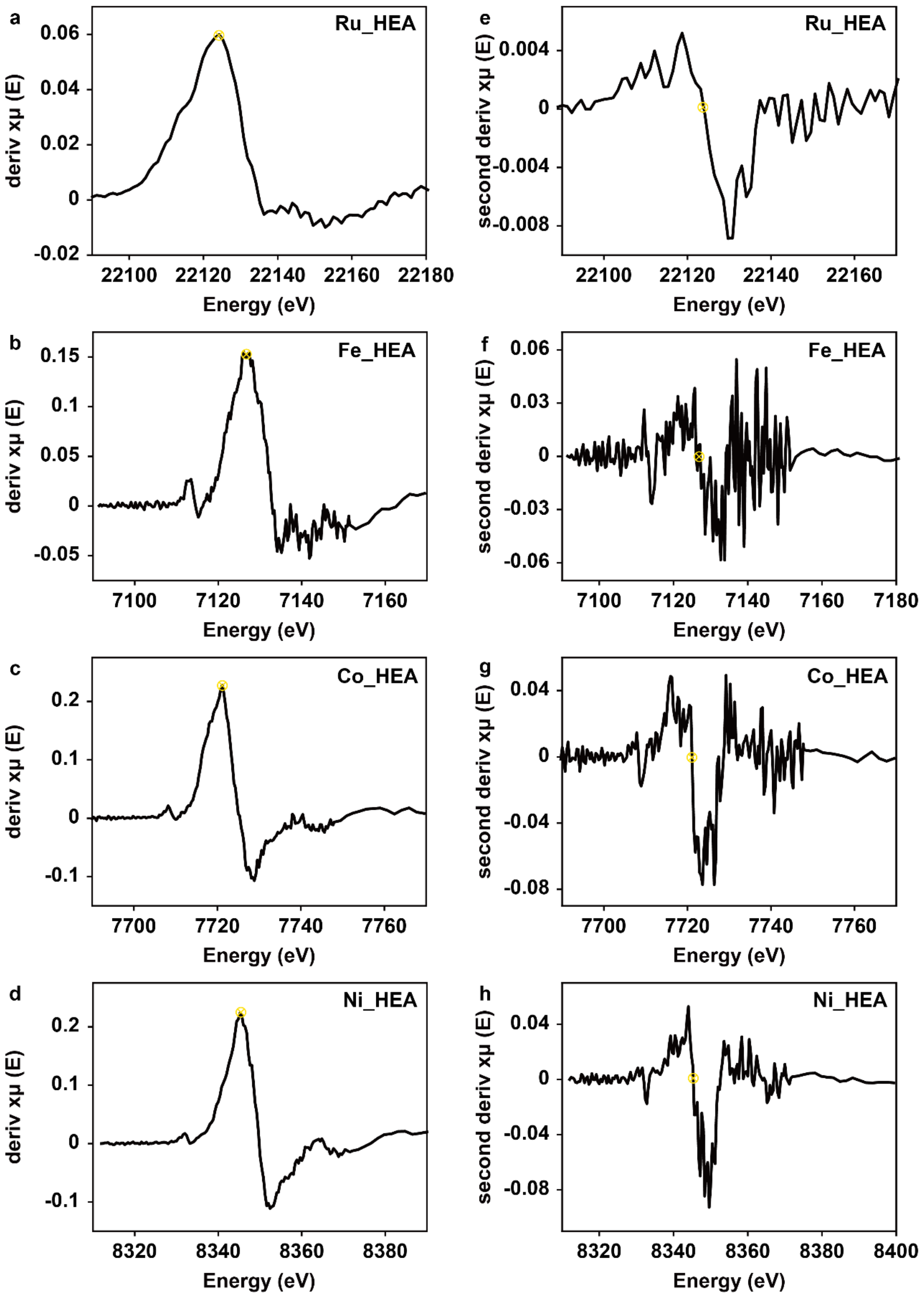

Figure S15. The Ru, Fe, Co, and Ni K-edge absorption positions determined by the inflection points of pre-edges for 1-nm PtRuFeCoNi HEA nanoparticles. (a, e) Ru K-edge, (b, f) Fe K-edge, (c, g) Co K-edge, and (d, h) Ni K-edge. The data were acquired by using "Athena software" and the energy of absorption edge was calibrated by the reference metallic foil corresponding to each element. The inflection points of pre-edges were identified where the second derivative equals to 0.

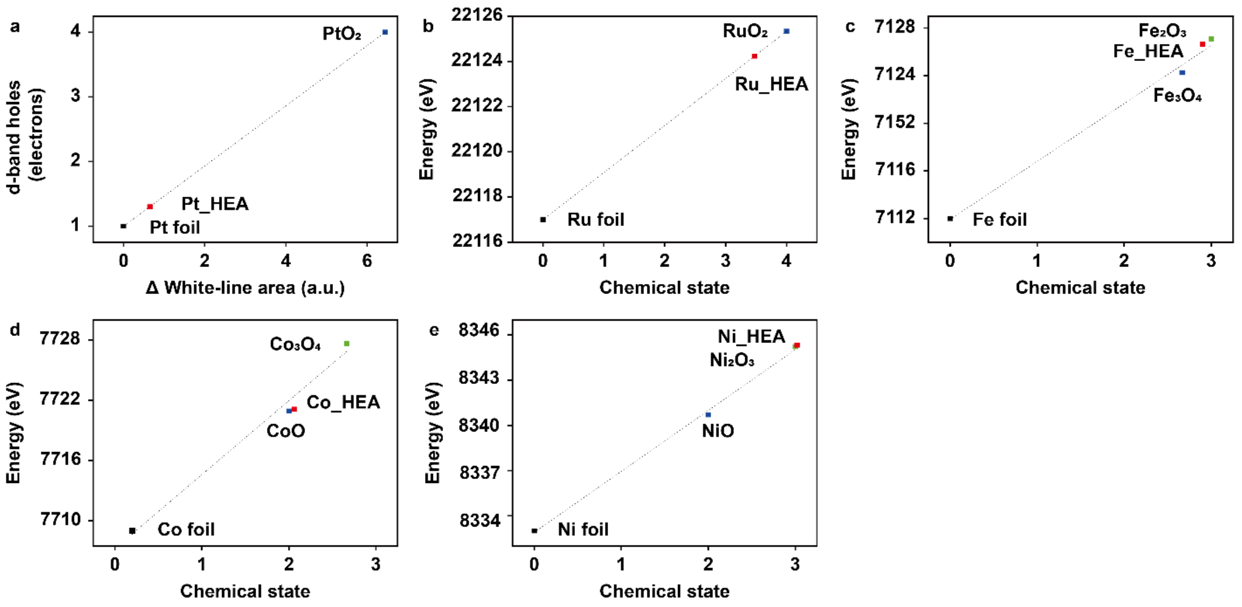

Figure S16. Determination of chemical states of Pt, Ru, Fe, Co, and Ni elements for the PtRuFeCoNi HEA nanoparticles through the XAS analysis. White-line peak area difference versus formal d-band hole count of (a) Pt L_3-edge. Absorption energy position versus chemical state of (b) Ru K-edge, (c) Fe K-edge, (d) Co K-edge, and (e) Ni K-edge.

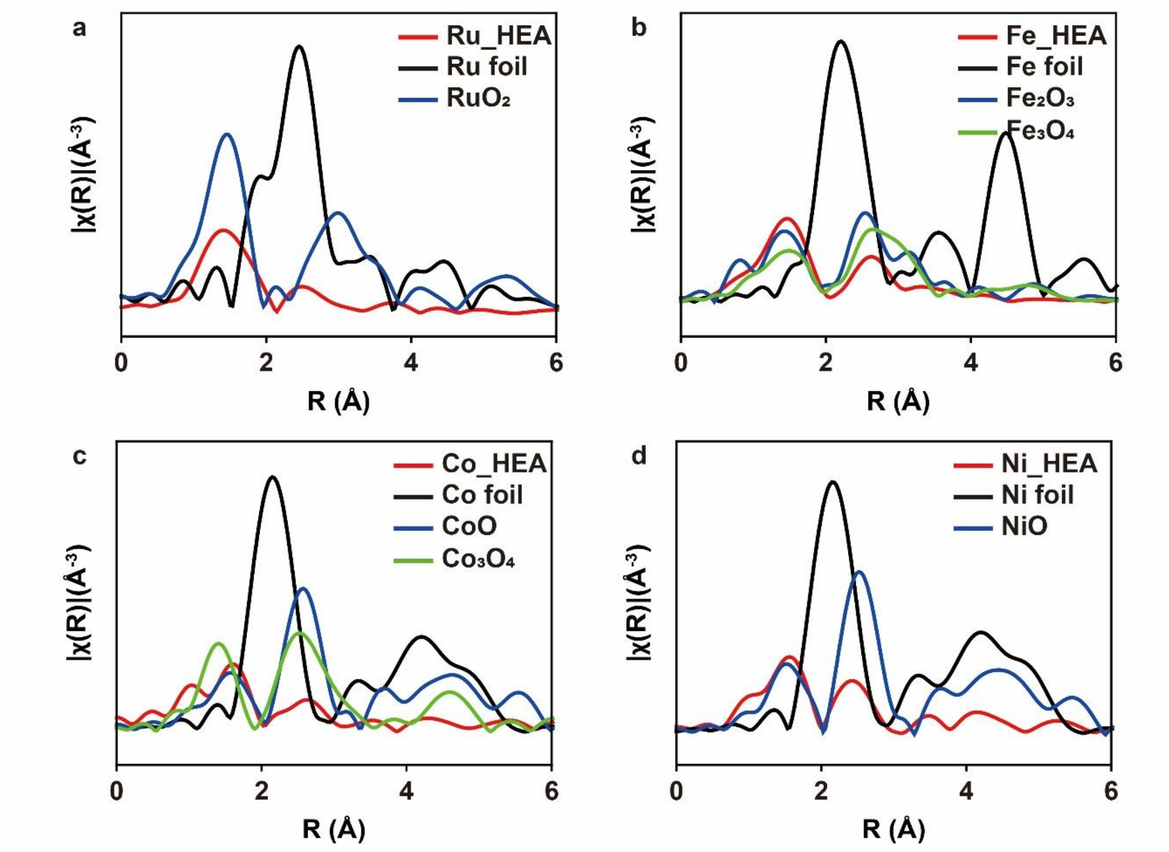

Figure S17. FT-EXAFS spectra of Ru, Fe, Co, and Ni the elements for 1-nm PtRuFeCoNi nanoparticles supported on N-doped CMK-3, along with their metallic foils and oxides references. (a) Ru K-edge, (b) Fe K-edge, (c) Co K-edge, and (d) Ni K-edge. All the FT-EXAFS spectra of PtRuFeCoNi nanoparticles display distinctive radial distances compared to the references, indicating a unique elemental distribution pattern.

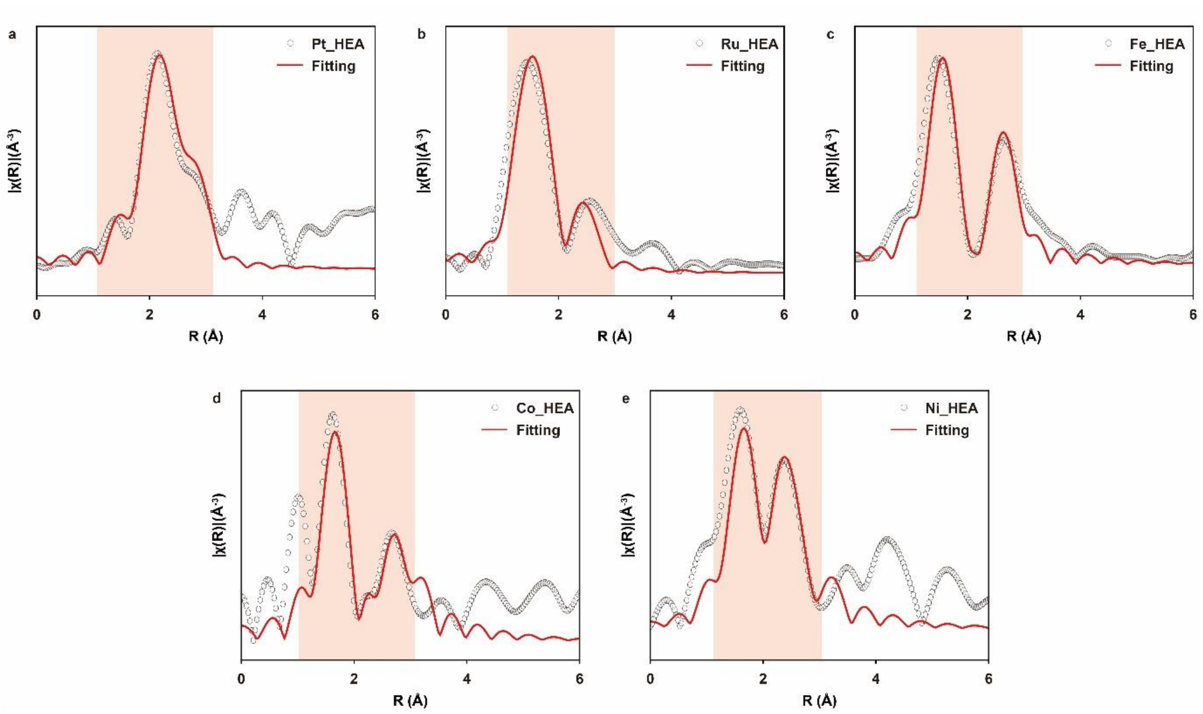

Figure S18. FT-EXAFS fitting analyses for the 1-nm PtRuFeCoNi HEA nanoparticles in R-space. (a) Pt_PtRuFeCoNi, (b) Ru_PtRuFeCoNi, (c) Fe_PtRuFeCoNi, (d) Co_PtRuFeCoNi, and (e) Ni_PtRuFeCoNi.

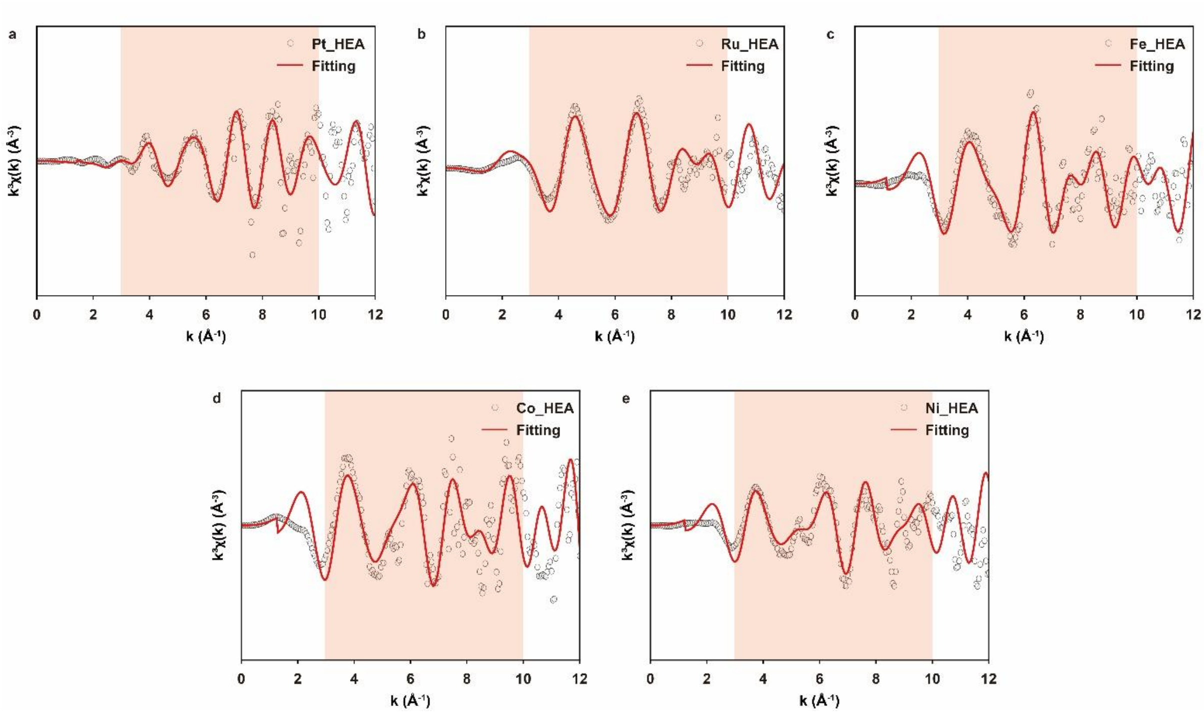

Figure S19. FT-EXAFS fitting analyses for the 1-nm PtRuFeCoNi HEA nanoparticles in k-space. (a) Pt_PtRuFeCoNi, (b) Ru_PtRuFeCoNi, (c) Fe_PtRuFeCoNi, (d) Co_PtRuFeCoNi, and (e) Ni_PtRuFeCoNi.

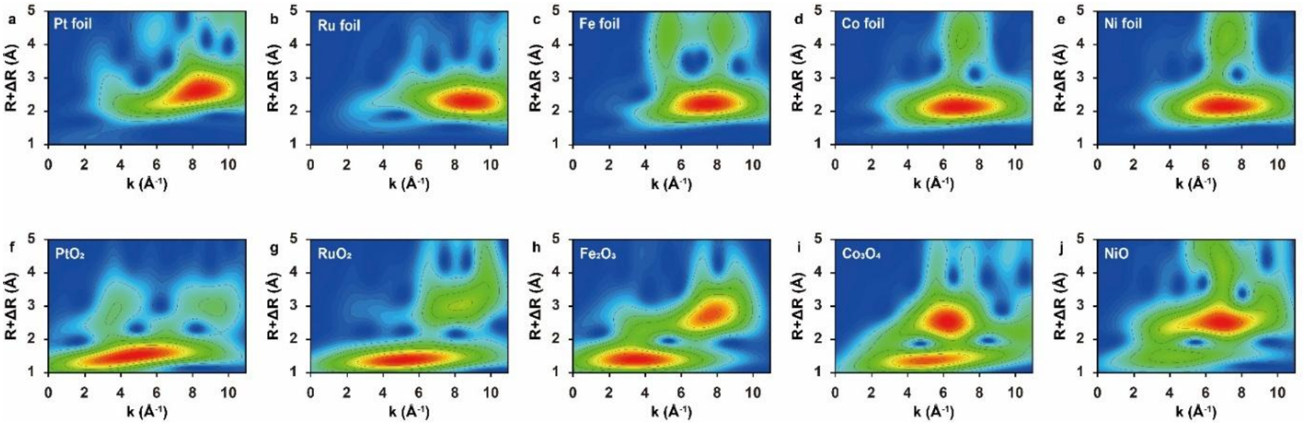

Figure S20. WT-EXAFS spectra of the metallic foils, along with their corresponding representative oxidation states. (a) Pt, (b) Ru, (c) Fe, (d) Co, (e) Ni, (f) PtO_2, (g) RuO_2, (h) Fe_2O_3, (i) Co_3O_4, and (j) NiO.

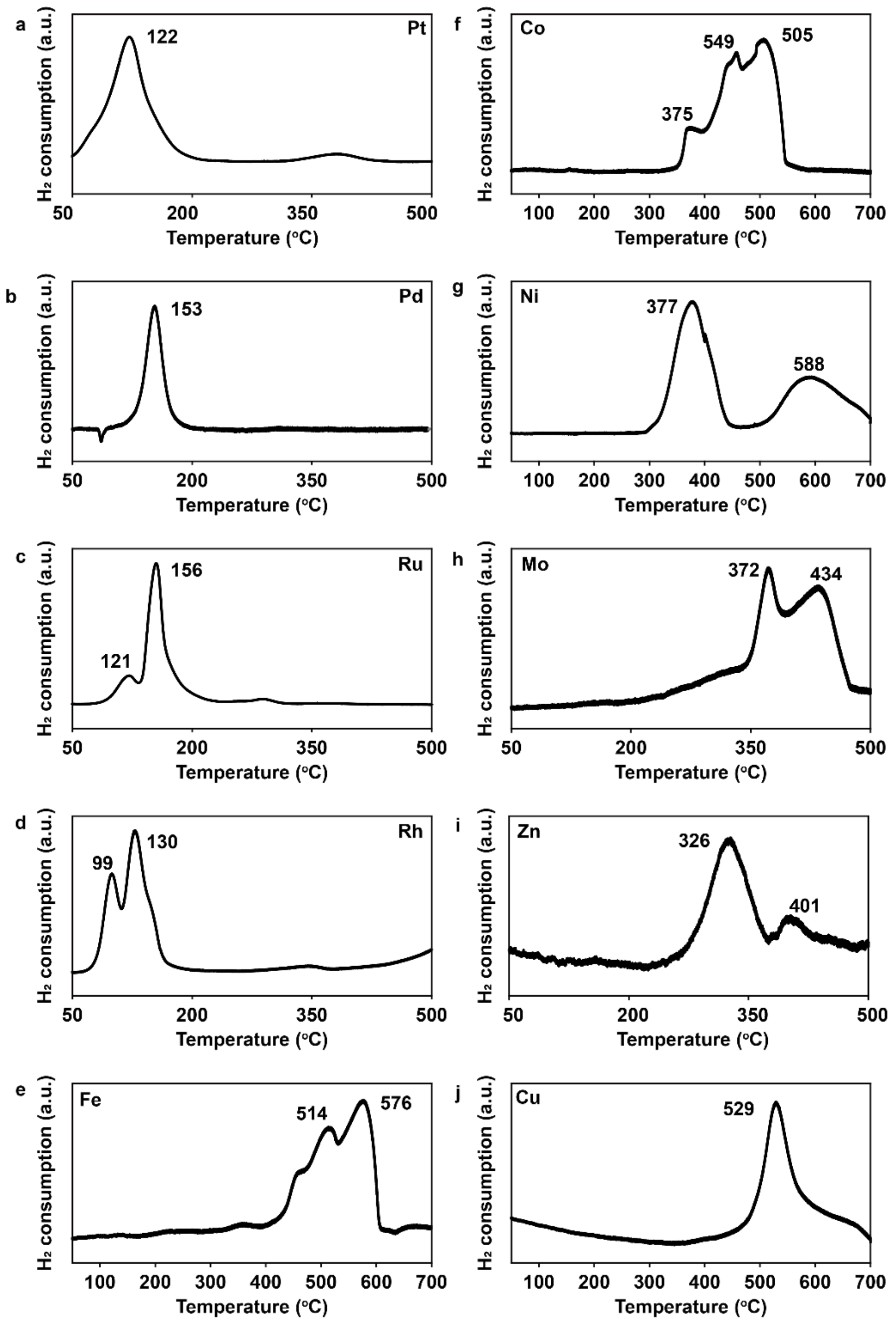

Figure S21. H_2-TPR analysis for mono-component samples. (a) Pt, (b) Pd, (c) Ru, (d) Rh, (e) Fe, (f) Co, (g) Ni, (h) Mo, (i) Zn, and (j) Cu after the impregnation into N-doped CMK-3. Due to their high reduction potentials (Table S3), Pt(IV), Pd(II), Ru(III), and Rh(III) metal precursors were primarily reduced at relatively low temperatures of around 122 °C, 153 °C, 156 °C, and 130 °C, respectively. In contrast, the reduction of Fe(III), Co(II), and Ni(II) ions, which have lower reduction potentials, began at the higher temperature of 300 °C and extended over a broad temperature range from 300-650 °C. For Mo(V), Zn(II), and Cu(II) precursors, the Mo(V) precursor exhibited two reduction peaks at 372 °C and 434 °C, the Zn(II) precursor reduced at approximately 326 °C, and the Cu(II) precursor reduced at a much higher temperature of around 529 °C.

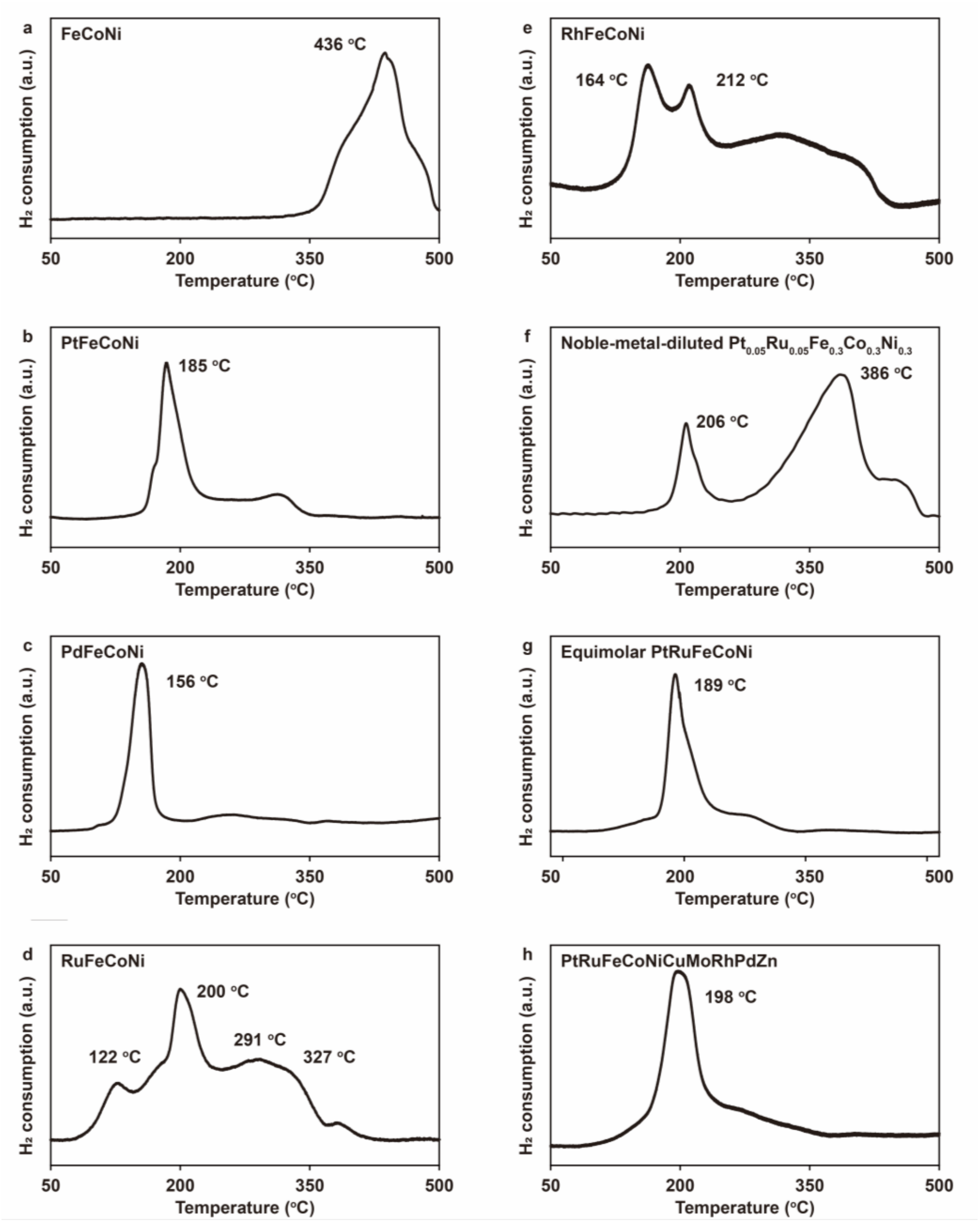

Figure S22. H_2-TPR analysis for different component samples. ternary (a) FeCoNi, quarternary (b) PtFeCoNi, (c) PdFeCoNi, (d) RuFeCoNi, and (e) RhFeCoNi, quinary (f) noble-metal-diluted $Pt_{0.05}Ru_{0.05}Fe_{0.3}Co_{0.3}Ni_{0.3}$, (g) equimolar PtRuFeCoNi, and denary (h) PtRuFeCoNiCuMoRhPdZn after the impregnation into N-doped CMK-3. The reduction of ternary-component precursors, consisting of mixed Fe(III), Co(II), and Ni(II) ions with relatively low reduction potentials (Table S3), commenced at elevated temperatures and reached a maximum at 436 ºC. The introduction of Pt(IV), Pd(II), Ru(III), or Rh(III) significantly lowered the reduction temperatures of the Fe(III), Co(II), and Ni(II) precursors compared to their single-metal counterparts or the original ternary-component mixtures. Notably, the noble-metal-diluted $Pt_{0.05}Ru_{0.05}Fe_{0.3}Co_{0.3}Ni_{0.3}$ sample exhibits a split reduction profile with higher-temperature peaks at 206 and 386 °C. In contrast, the quinary PtRuFeCoNi and denary PtRuFeCoNiCuMoRhPdZn systems show single main reduction peaks at 189 and 198 °C, respectively.

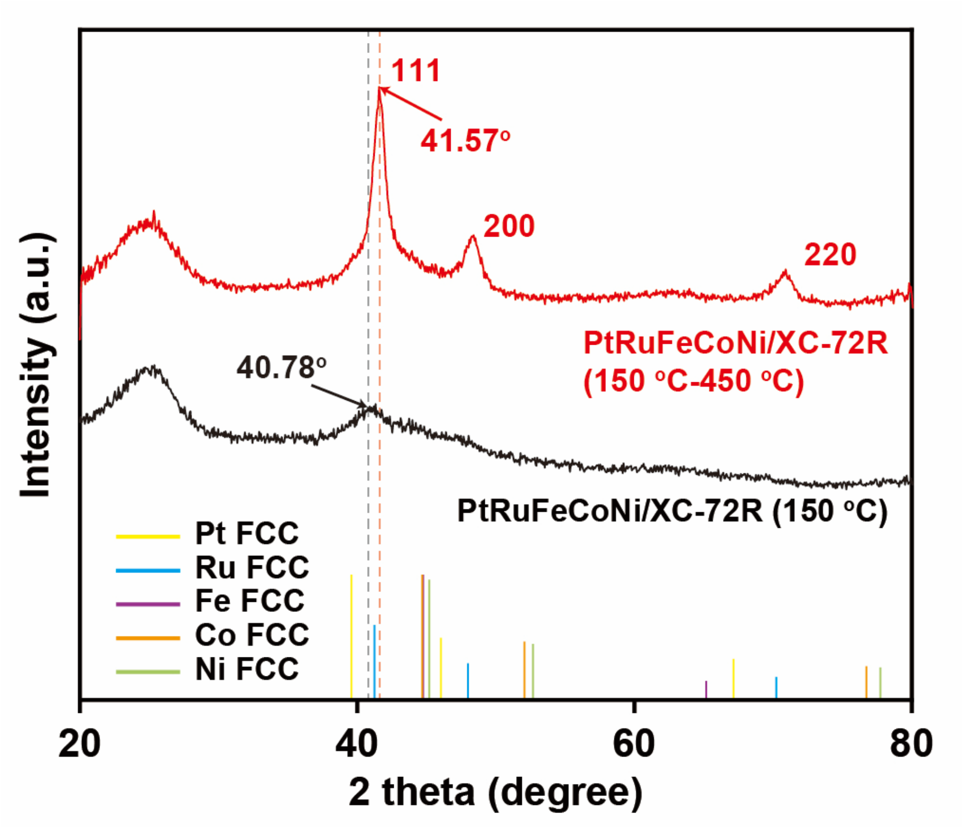

Figure S23. XRD patterns tracking the intermediate stages of the sequential nucleation and homogenization process for PtRuFeCoNi HEA nanoparticles supported on Vulcan XC-72R carbon. The black curve represents the intermediate state isolated after annealing at 150 °C for 30 min, showing a broad diffraction peak at $2\theta = 40.78°$, which can be assigned to the FCC(111) reflection. This is consistent with the preferential reduction and nucleation of a noble-metal-rich, likely Pt/Ru-rich, intermediate phase. The red curve represents the final state obtained after the full thermal treatment program ending at 450 °C, where the primary diffraction peak of FCC(111) becomes more intense and shifts to a higher angle of $2\theta = 41.57°$. This peak shift indicates lattice contraction, supporting the thermal interdiffusion and incorporation of smaller 3d transition metals, including Fe, Co, and Ni, into the host lattice. These results are consistent with the formation of a more homogenized FCC solid-solution phase.

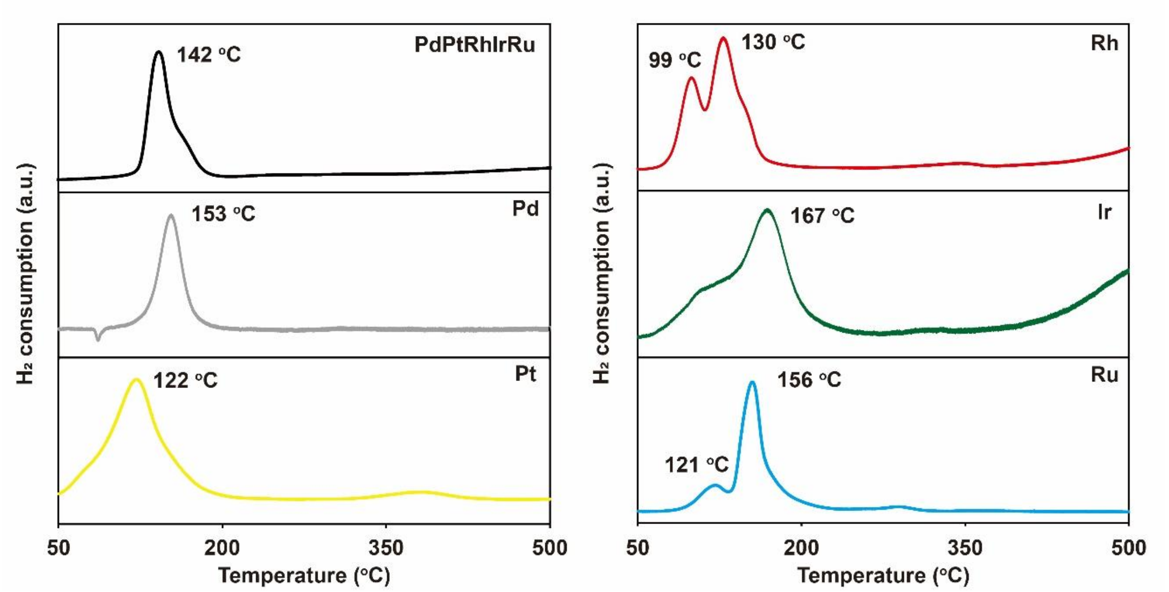

Figure S24. H_2-TPR profiles of PdPtRhIrRu HEA nanoparticles and the corresponding monometallic samples (Pd, Pt, Rh, Ir, and Ru). The reduction peak of Pd appears at 153 °C, while Pt shows a lower reduction temperature at 122 °C. Rh and Ru both exhibit two reduction features, located at 99 and 130 °C for Rh, and at 121 and 156 °C for Ru, respectively, indicating multiple reducible species or environments. Ir displays the highest reduction temperature among the five elements, with a peak at 167 °C. In contrast, the PdPtRhIrRu HEA nanoparticles show a single dominant reduction peak centered at 142 °C, which lies between the reduction temperatures of the constituent monometallic catalysts, suggesting modified reduction behavior arising from strong inter-element interactions in the HEA structure.

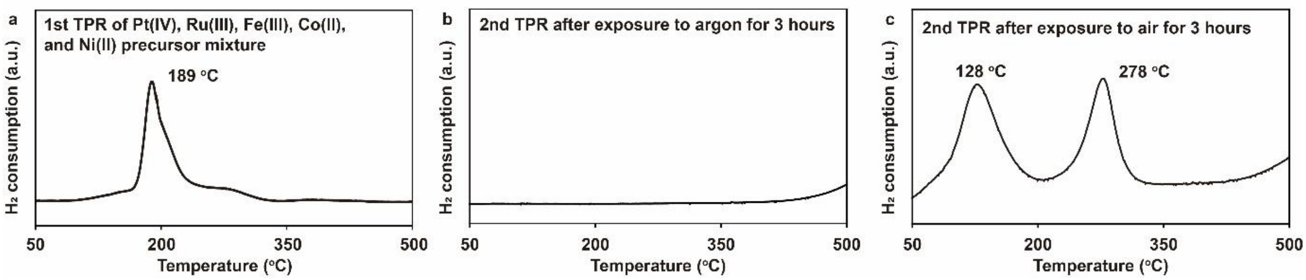

Figure S25. H_2-TPR experiments verifying the complete reduction of the metal precursors. (a) TPR profiles of the same precursors were obtained again after cooling to room temperature under Ar atmosphere without air exposure. (b) TPR profiles of the PtRuFeCoNi metal precursors were first recorded, then cooled to room temperature, stored in air for 3 hours, and subsequently re-tested by TPR. For the samples exposed to air, a distinct H_2 consumption peak appears at 128 °C, accompanied by another peak at 278 °C, indicating surface reoxidation. In contrast, the samples cooled under Ar and immediately subjected to a second TPR measurement exhibit no apparent reduction peaks across the entire temperature range. These results confirm that the oxidation observed in PtRuFeCoNi originates from the high oxophilicity of Fe, Co, and Ni upon air exposure, rather than from incomplete reduction during synthesis.

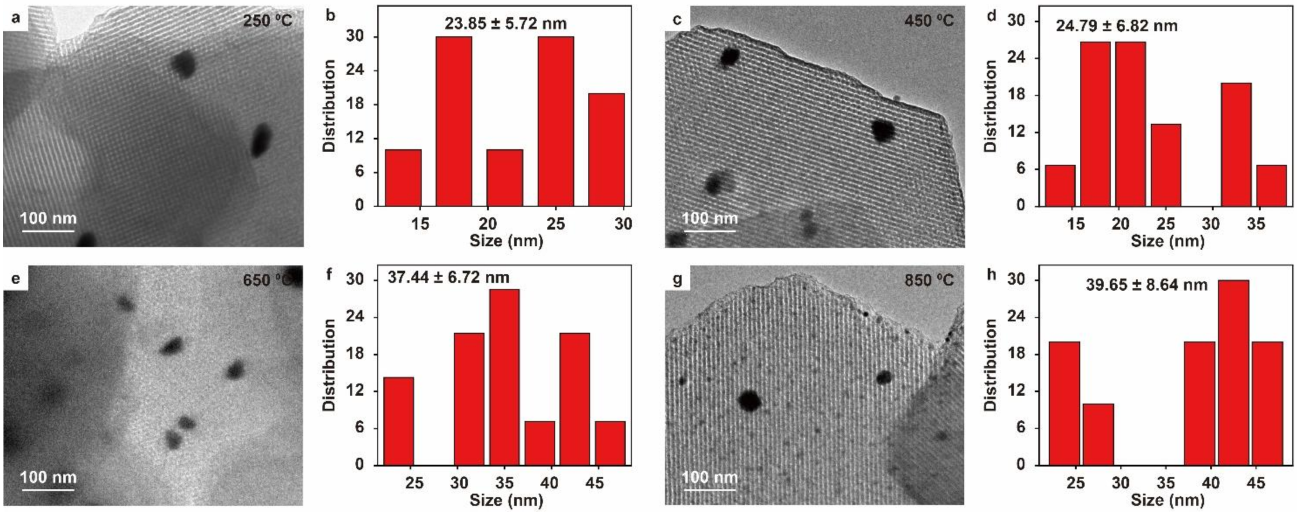

Figure S26. TEM images and size distributions for PtRuFeCoNi nanoparticles supported on N-doped CMK-3 synthesized in Ar flow at various reaction temperatures. (a, b) 250 ºC, (c, d) 450 ºC, (e, f) 650 ºC, and (g, h) 850 ºC.

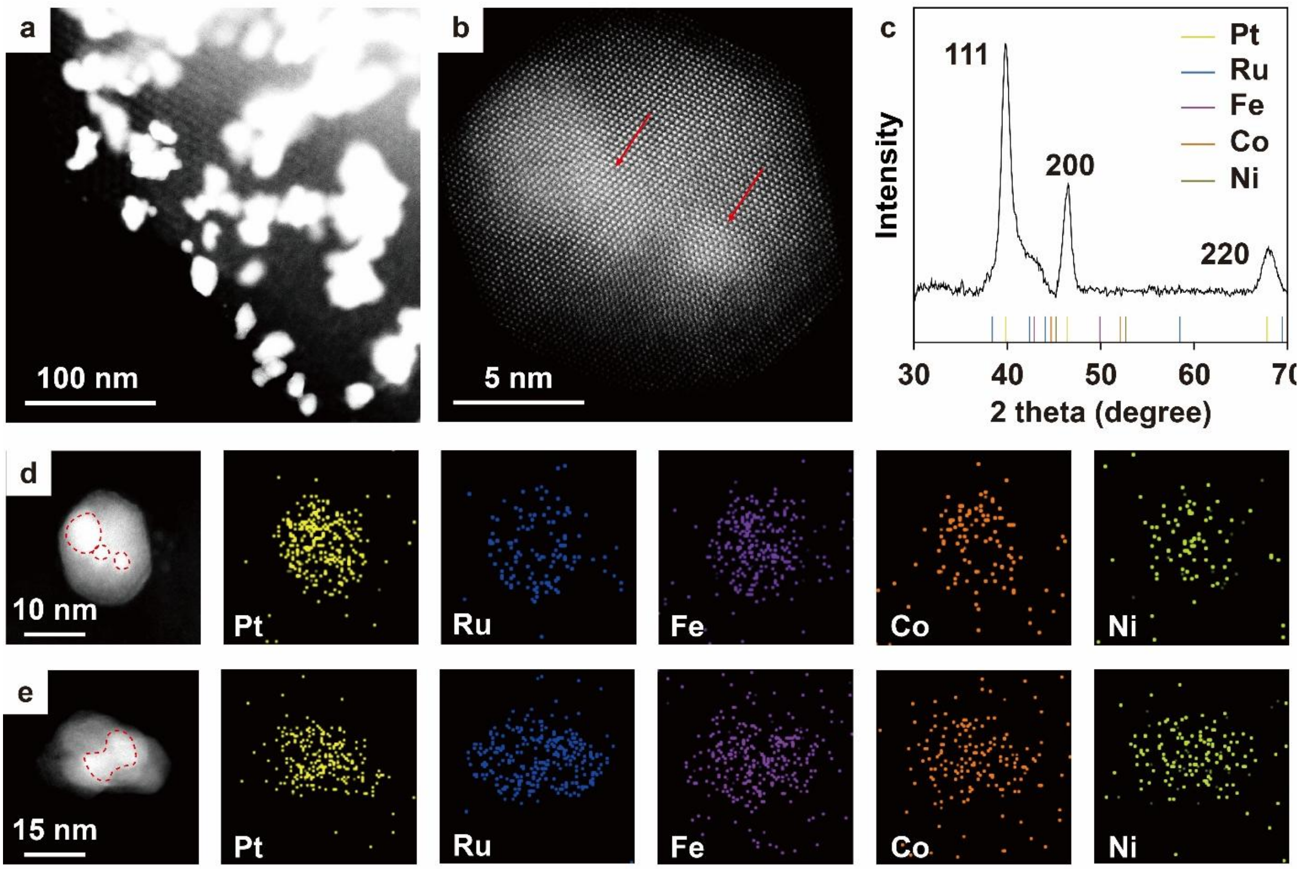

Figure S27. Material characterizations of PtRuFeCoNi nanoparticles with phase separation synthesized at Ar atmosphere. (a) Low and (b) high magnification HADDF-STEM images, (c) XRD analysis, and (d, e) EDS mappings of PtRuFeCoNi nanoparticles. The Pt-based phase results in the brightest region in the HADDF-STEM image due to the higher Z-contrast of Pt, exhibiting the phase separation during the pyrolysis process under the Ar atmosphere.

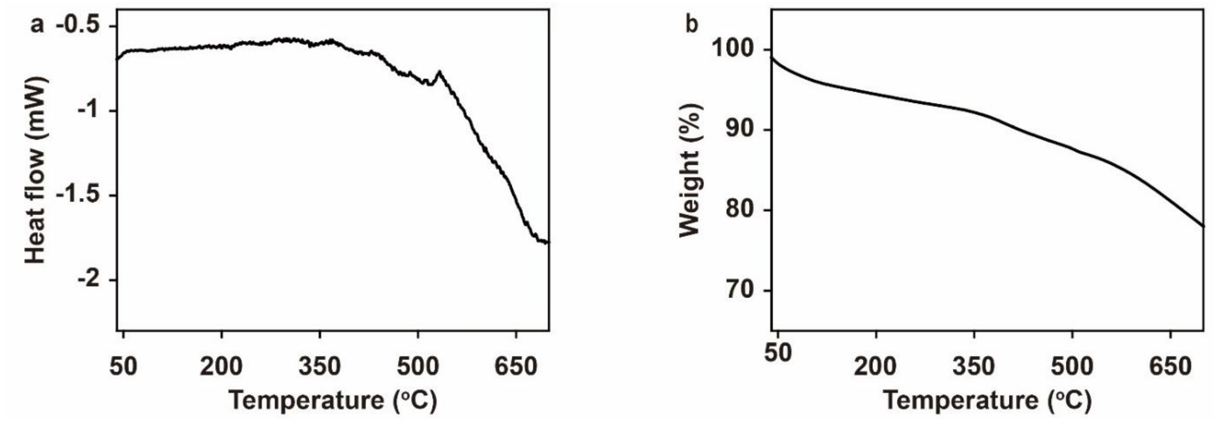

Figure S28. Thermal decomposition of PtRuFeCoNi nanoparticles supported on N-doped CMK-3 in an inert Ar atmosphere. (a) DSC and (b) TGA analysis.

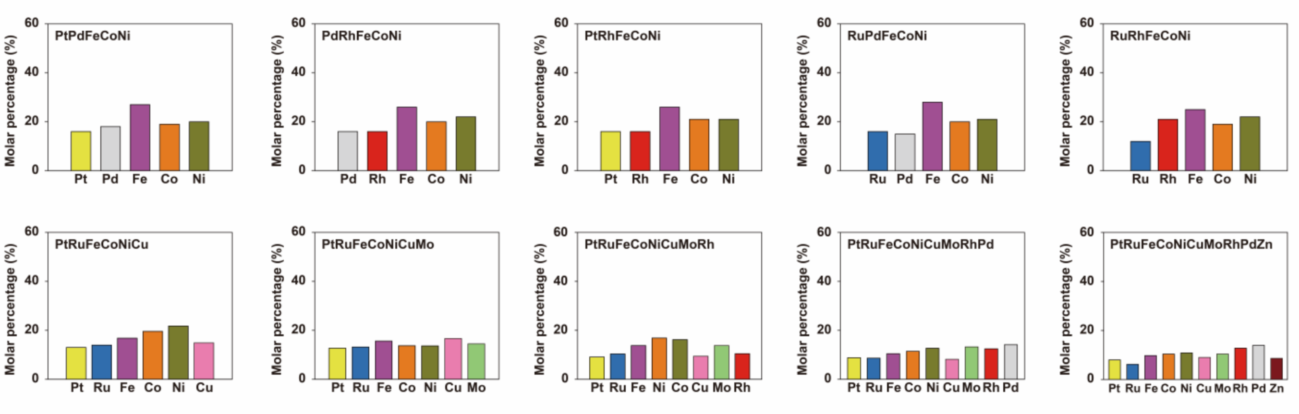

Figure S29. ICP-OES analysis of multicomponent HEA nanoparticles from quinary to denary systems.

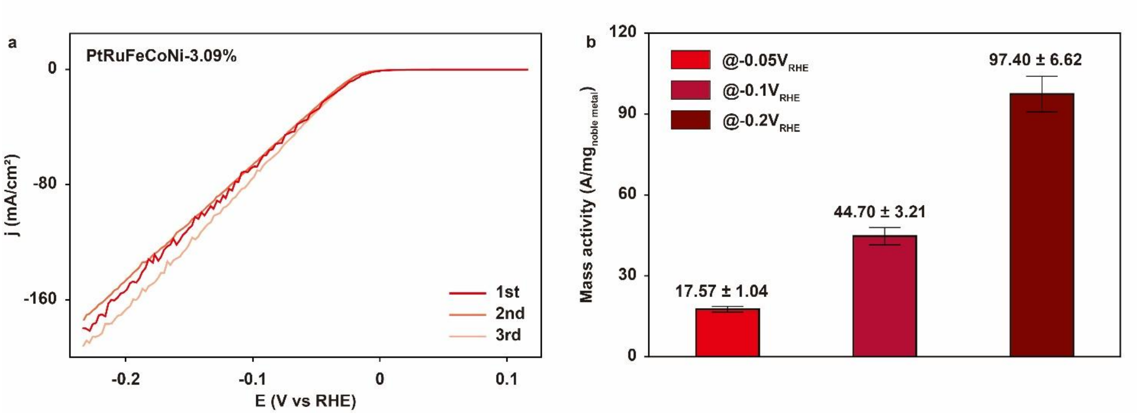

Figure S30. Reproducibility of HER activity measurements for PtRuFeCoNi-3.09% catalysts. Intrinsic HER activities of (a) PtRuFeCoNi-3.09%, which were measured three times to obtain error bars. (b) Corresponding mass activities of PtRuFeCoNi-3.09% with standard deviations shown as error bars. For the catalysts, three independent catalyst inks were separately prepared and ultrasonicated for 60 minutes in an ice bath to ensure homogeneous dispersion, resulting in reliable and representative error estimation.

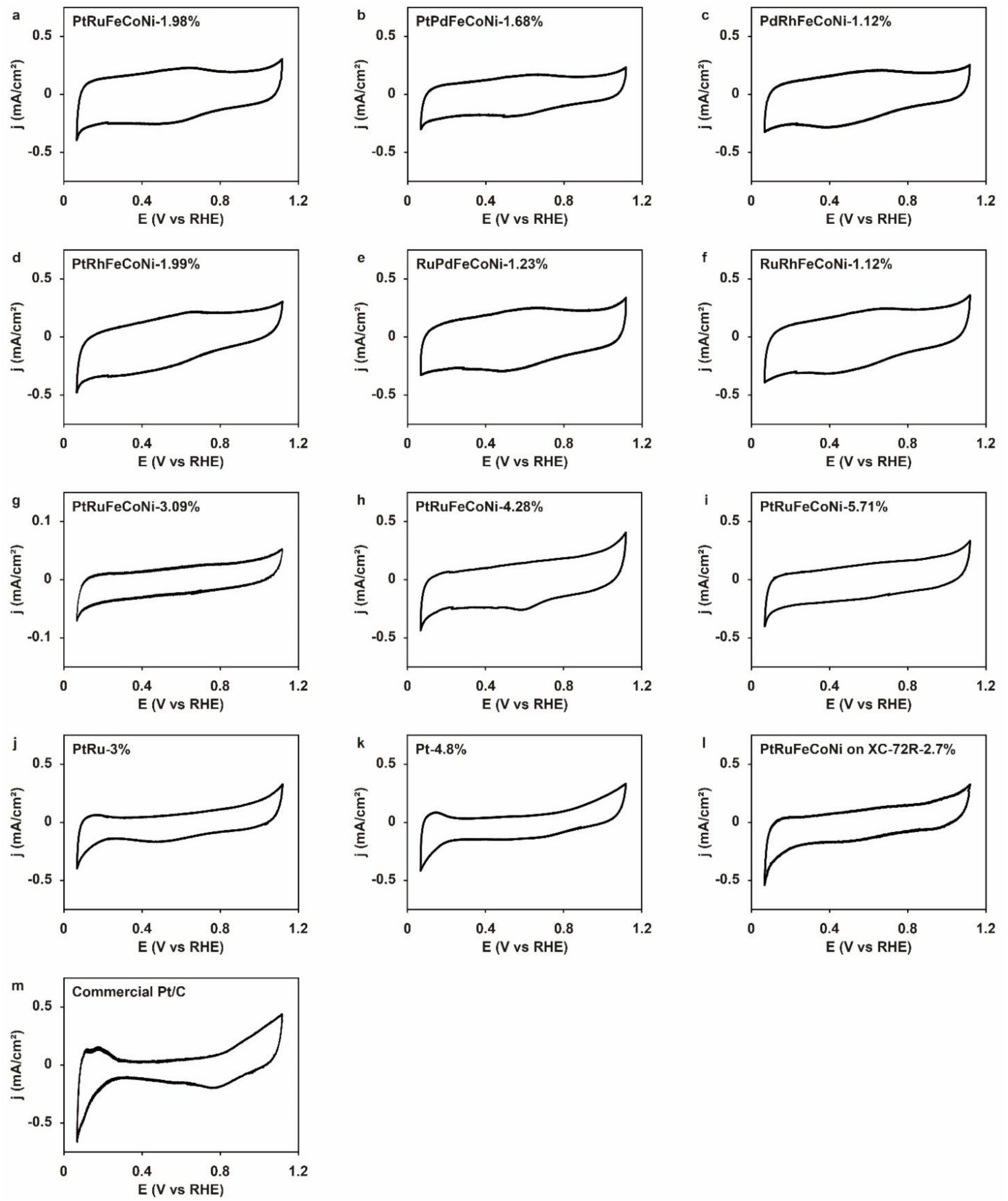

Figure S31. CV curves of the monometallic, bimetallic, trimetallic, and multimetallic catalysts supported N-doped CMK-3, along with the PtRuFeCoNi HEA nanoparticles supported on XC-72R and commercial Pt/C. The CV curves were measured at N_2-saturated 0.5 M H_2SO_4 with a scanning rate of 50 mV s^{-1}. Notably, owing to the very low loading of the HEA nanoparticles, reliable ECSA estimation from the hydrogen underpotential deposition (HUPD) region is inherently difficult.

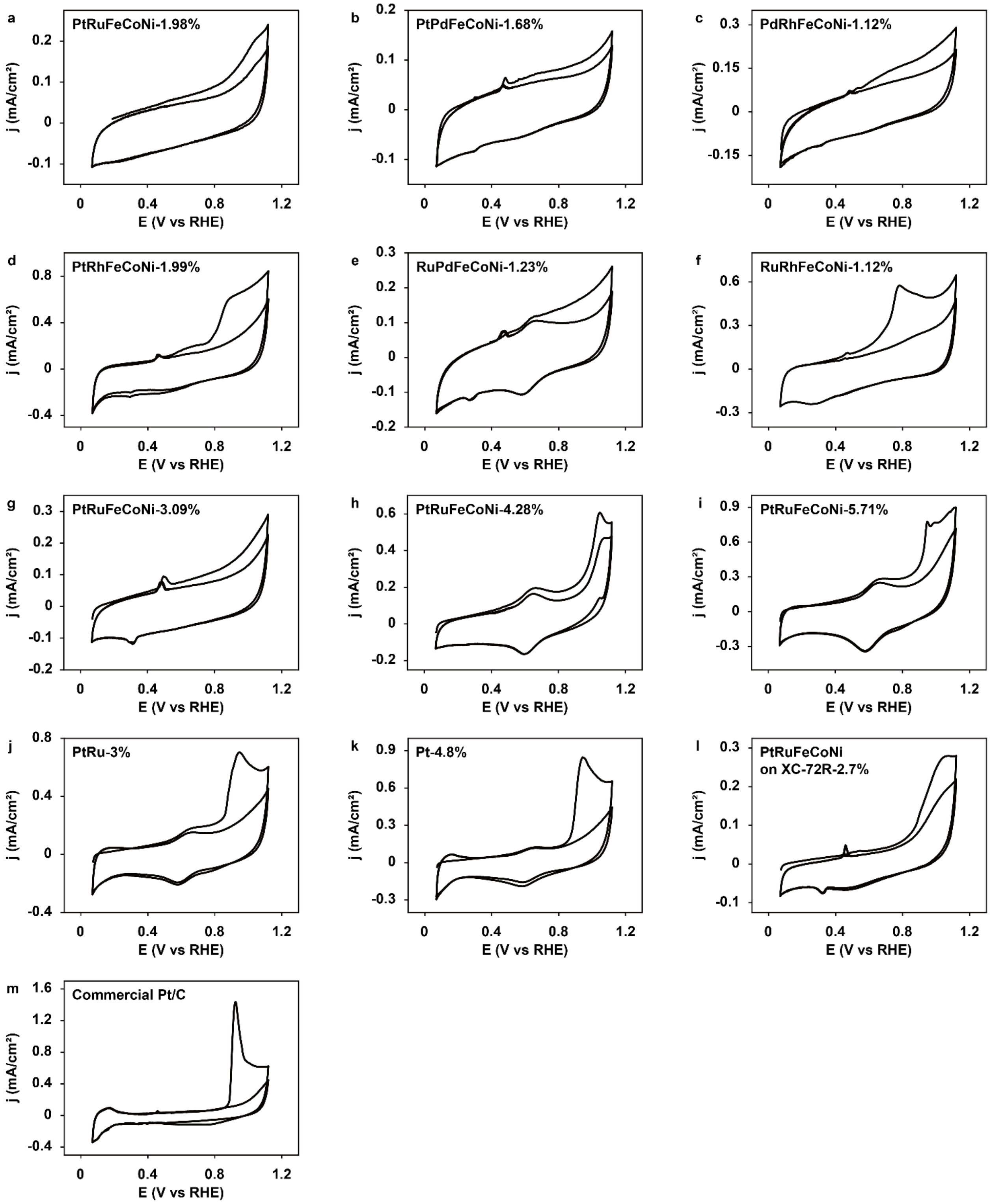

Figure S32. CO-stripping measurements of the monometallic, bimetallic, trimetallic, and multimetallic catalysts supported N-doped CMK-3, along with the PtRuFeCoNi HEA nanoparticles supported on XC-72R and commercial Pt/C. The CO-stripping experiment was performed in 0.5 M H_2SO_4. First, a high-purity CO gas is purged into the electrolyte for 30 minutes to form a CO-saturated solution. Then the electrode potential is held at 0.1 V_{RHE} for 10 minutes to allow CO to adsorb on the

catalyst surface. Subsequently, N_2 is purged for 20 min to remove residual CO from the solution. Finally, the CO-stripping curves between 0.05 and 1.05 V_{RHE} were obtained at a scan rate of 50 mV s^{-1} for two cycles. Although the CO-stripping areas of the catalytic materials show minimal differences, the low metal content used in our system provides a significant advantage, contributing to the exceptional catalytic performance of the materials (Figure S32). Due to the multicomponent nature of HEAs, determining an absolute ECSA is complex, as the theoretical charge required for monolayer adsorption varies across constituent elements (e.g., approximately 420 μC cm^{-2} for CO-stripping on polycrystalline Pt).[5-7] Furthermore, the intrinsic "cocktail effect" and local coordination environments in HEAs can induce non-linear adsorption behaviors, making it difficult to define a universal charge constant for such high-entropy surfaces.[8, 9] For consistency and comparative analysis, the ECSA values in this study were estimated by assuming a uniform charge density equivalent to that of polycrystalline Pt for CO oxidation (420 μC cm^{-2}), according to ECSA = Q_{CO}/mC, where Q_{CO} represents the integrated charge of the CO-stripping peak after background subtraction, m is the metal mass loading on the electrode, and C is the specific charge associated with a CO monolayer on Pt. On this basis, ECSA was used only as an approximate indicator of accessible active sites, whereas mass activity was considered the more appropriate primary metric for assessing the intrinsic performance of the HEA catalysts.

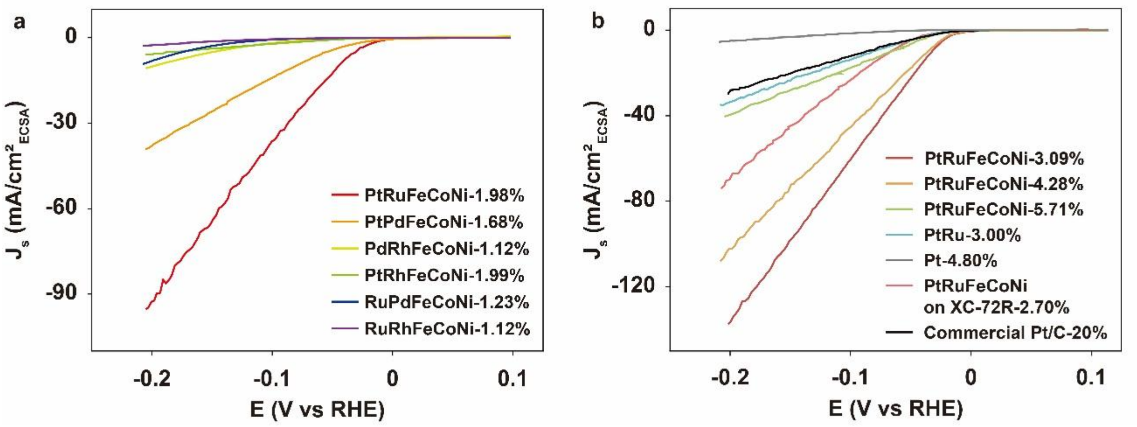

Figure S33. ECSA-normalized specific activities of 1-nm HEA nanoparticles for HER. Because the ultrasmall particle size of approximately 1 nm leads to a poorly defined HUPD region in the cyclic voltammograms, the ECSA of each HEA catalyst was determined by CO-stripping experiments (Figures S31 and S32).

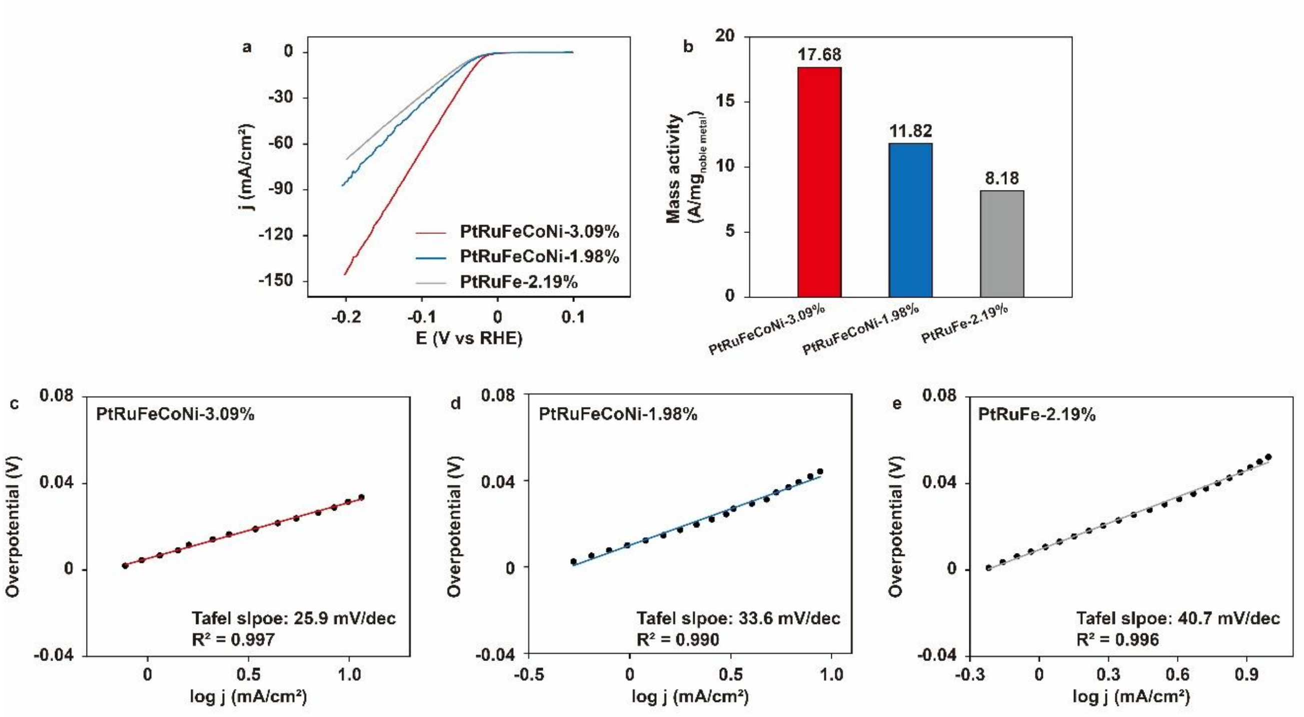

Figure S34. HER performance and Tafel slope of quinary PtRuFeCoNi-3.09%, -1.98%, and trimetallic PtRuFe-2.19% nanoparticles. The (a) LSV curves and (b) mass activity at -0.05V_{RHE} of PtRuFeCoNi-3.09%, -1.98%, and PtRuFe-2.19%. Tafel slopes of (c) PtRuFeCoNi-3.09%, (d) PtRuFeCoNi-1.98%, and (e) PtRuFe-2.19%. In comparison, both quinary HEA catalysts exhibit higher intrinsic HER current densities and mass activities than the trimetallic PtRuFe-2.19% counterpart. Moreover, the quinary compositions display smaller Tafel slopes, suggesting that the incorporation of Co and Ni not only increases the configurational entropy but also accelerates the reaction kinetics of the HER, thereby leading to enhanced catalytic performance in the high-entropy configuration.

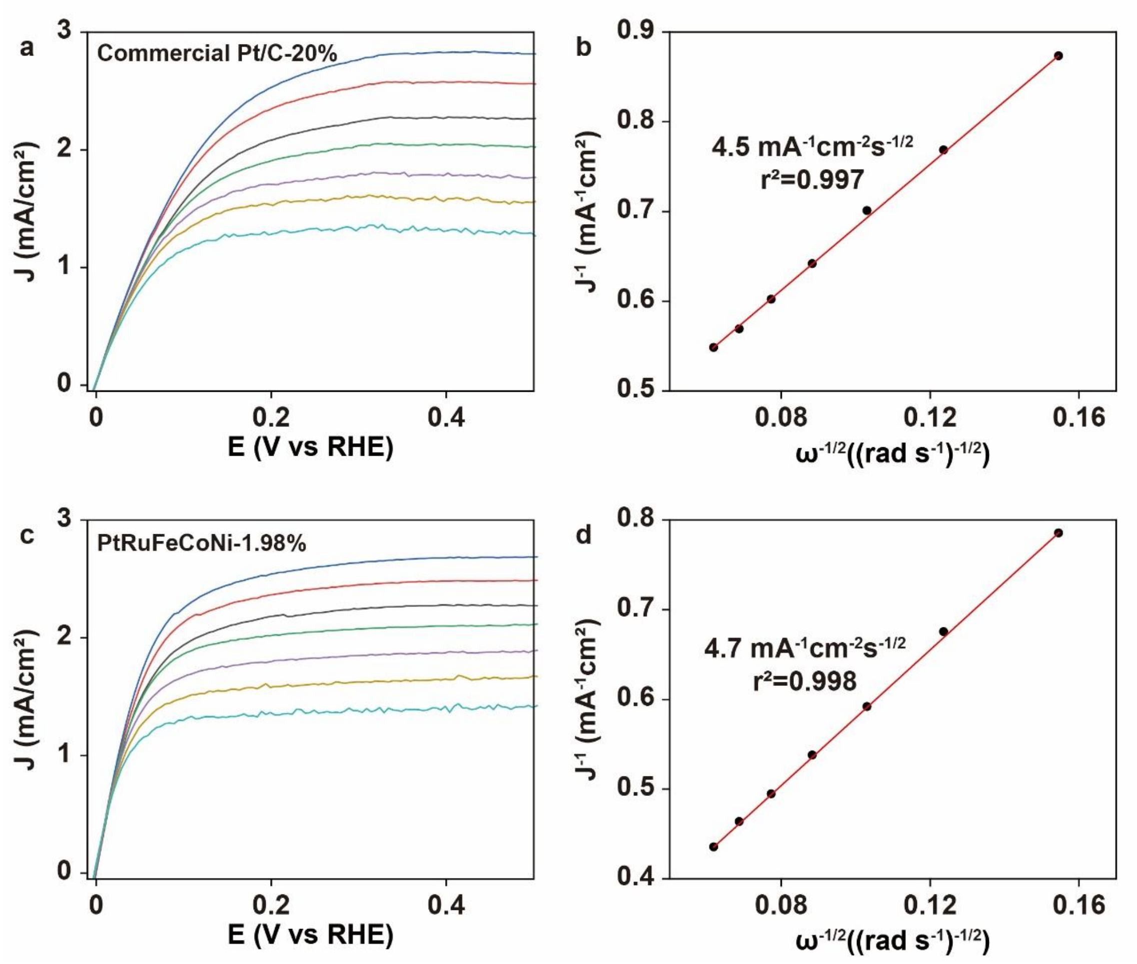

Figure S35. Polarization curves of (a) commercial Pt/C and (c) PtRuFeCoNi HEA nanoparticles recorded at different rotation speeds (ω) of 400, 625, 900, 1225, 1600, 2025, and 2500 rpm, and the corresponding Koutecky-Levich plots (b, d) at an overpotential of 100 mV. [10] In panels (a) and (c), the curves from bottom to top correspond to increasing rotation speeds from 400 to 2500 rpm.

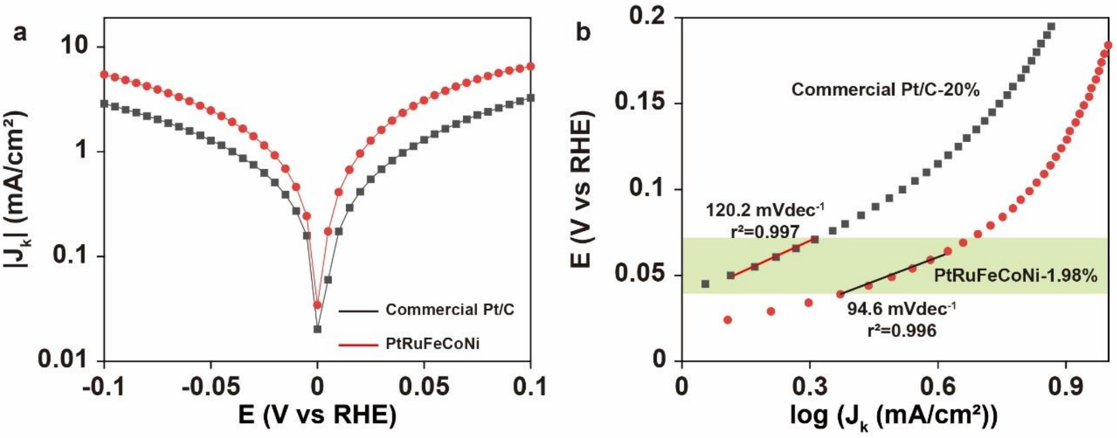

Figure S36. Tafel plot analysis of the kinetic current densities of commercial Pt/C and HEA nanoparticles normalized to the geometric disk area. (a) Tafel plot and (b) Tafel slope analysis. The Tafel slope of commercial Pt/C and HEA nanoparticles corresponds to 120.2 mV dec^{-1} and 64.6 mV dec^{-1}.[11, 12]

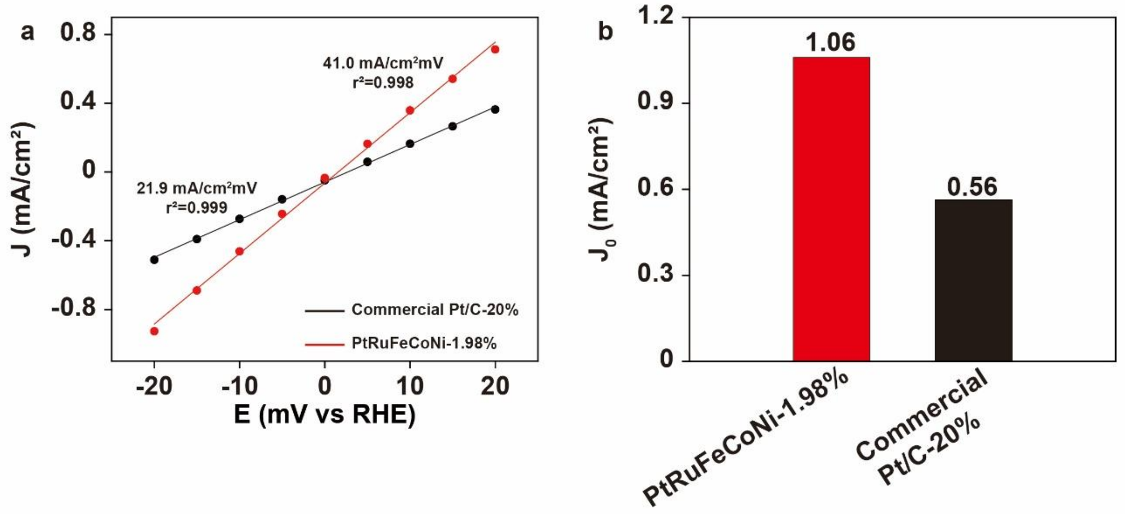

Figure S37. (a) Linear fitting curves in micro-polarization region (-20 to 20 mV_{RHE}) of PtRuFeCoNi HEA nanoparticles and commercial Pt/C. (b) Comparison of the exchange current density (J_0) normalized to ECSAs of PtRuFeCoNi HEA nanoparticles and commercial Pt/C.

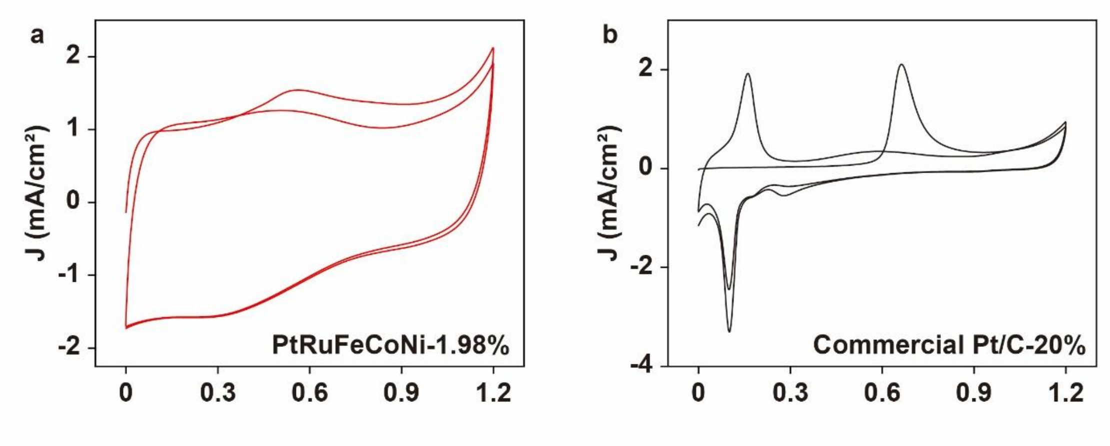

Figure S38. Comparative electrochemical CO-stripping voltammetry of the catalysts measured in N_2-saturated 0.1 M KOH at a scan rate of 50 mV/s. CO-stripping profiles of (a) 1-nm PtRuFeCoNi-1.98% HEA nanoparticles and (b) commercial Pt/C-20%. Commercial Pt/C exhibits a sharp anodic CO oxidation peak centered at approximately 0.66 V_{RHE}, consistent with CO oxidation on a relatively uniform Pt-dominated surface. In contrast, PtRuFeCoNi-1.98% shows a broader and more continuous oxidation profile with a lower onset potential, suggesting a wider distribution of OH-associated adsorption environments across the multielement HEA surface.

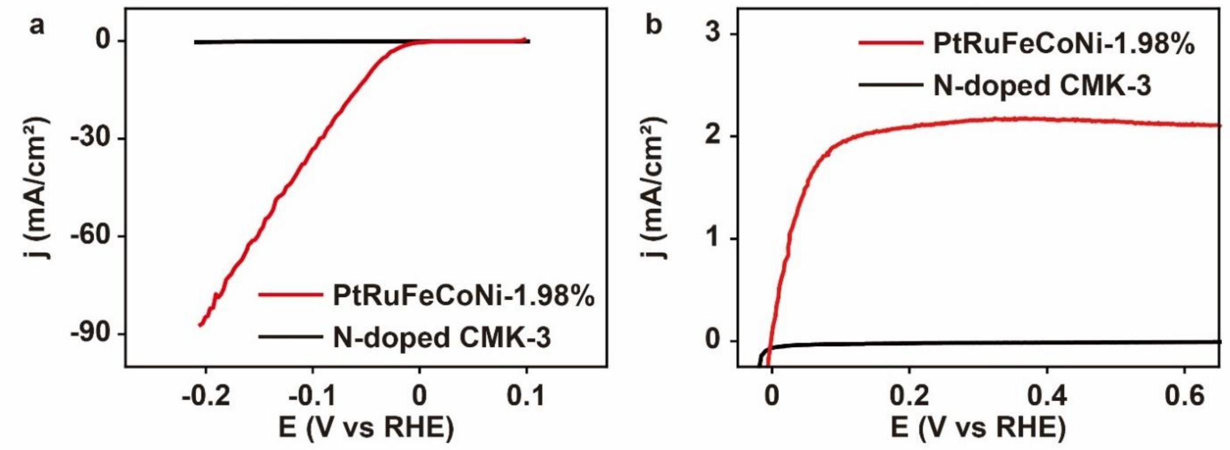

Figure S39. The electrochemical test of N-doped CMK-3. (a) HER in 0.5 M H_2SO_4 and (b) HOR in 0.1 M KOH. The pure N-doped CMK-3 exhibited almost no catalytic activity in either the HER or HOR measurements. The poor activity of pure N-doped CMK-3 indicates that the observed catalytic activities in the electrocatalytic measurements were mainly attributed to the 1-nm HEA nanoparticles (Figure 7) embedded uniformly in N-doped CMK-3.

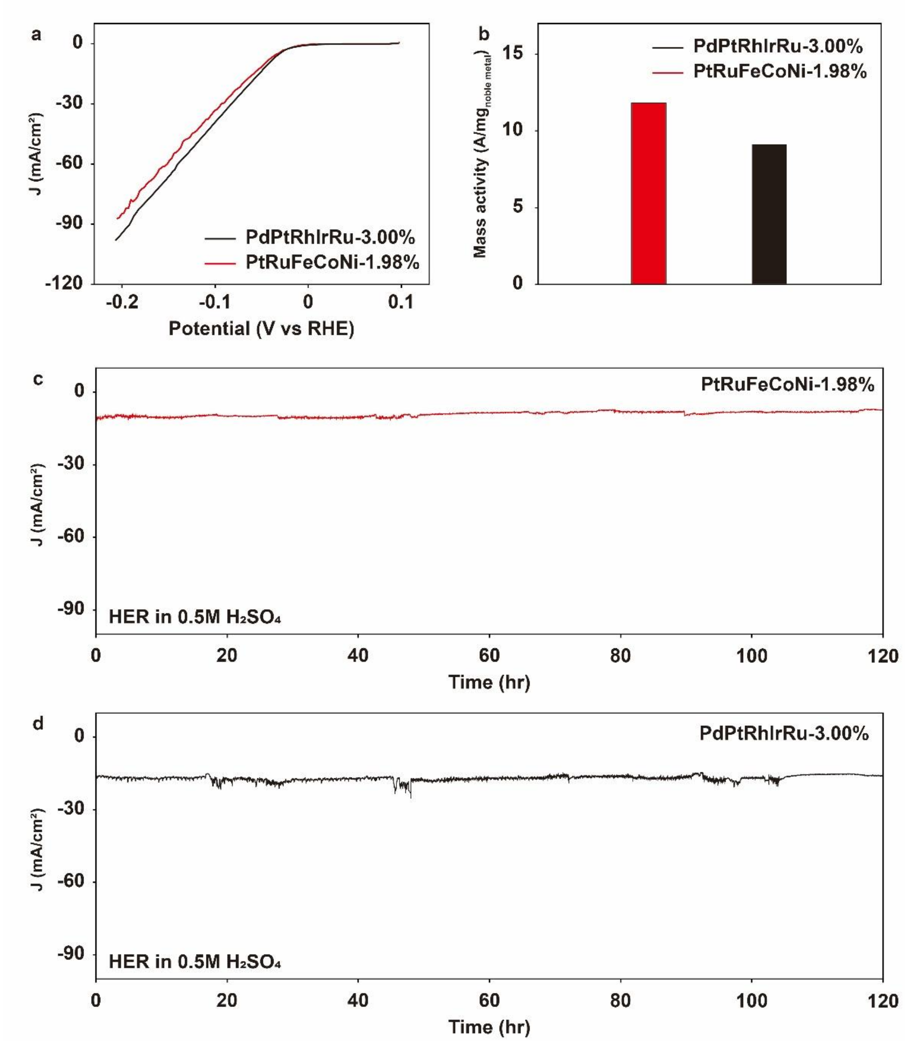

Figure S40. Comparison of acidic HER performance of PtRuFeCoNi-1.98% and PdPtRhIrRu-3.00% catalysts in 0.5 M H_2SO_4. (a) LSV curves. (b) Mass activity. Long-term chronoamperometric stability at -0.07 V_{RHE} for 120 h in 0.5 M H_2SO_4 for (c) PtRuFeCoNi-1.98% and (d) PdPtRhIrRu-3.00%.

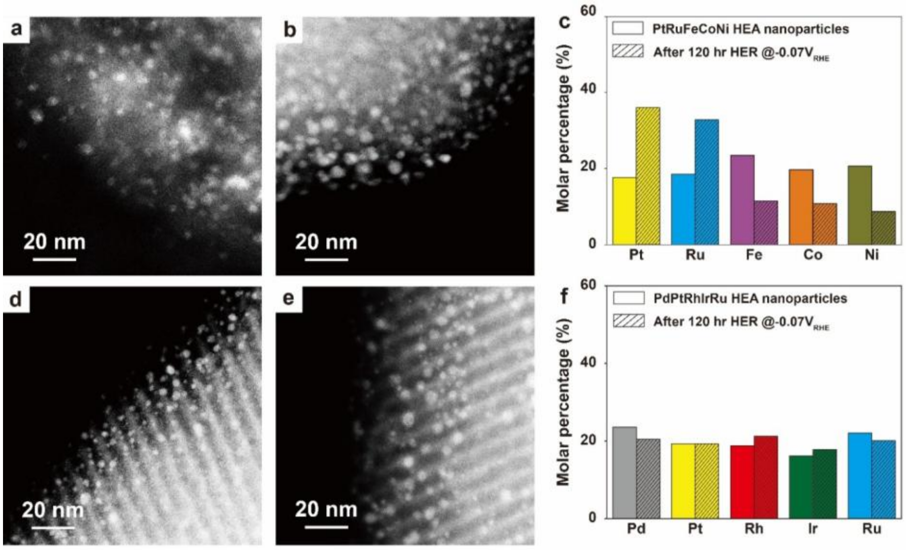

Figure S41. Post-reaction characterizations of PtRuFeCoNi and PdPtRhIrRu HEA nanoparticles after a 120 h HER durability test in acidic electrolyte. (a) HAADF-STEM image of PtRuFeCoNi HEA nanoparticles before stability testing. (b) HAADF-STEM image after 120 h HER operation, showing noticeable particle coarsening compared with the initial average size of 3.00 nm (original of 1.12 nm), indicative of sintering during electrochemical process. (c) ICP-OES analysis of PtRuFeCoNi HEA nanoparticles before and after durability testing, revealing decreased atomic fractions of Fe, Co, and Ni after HER, consistent with preferential dissolution of these 3d transition metals in acidic media. (d) HAADF-STEM image of PdPtRhIrRu HEA nanoparticles before stability testing. (e) HAADF-STEM image after 120 h HER operation, showing no obvious change in particle size (from 1.48 nm to 1.92 nm). (f) ICP-OES analysis of PdPtRhIrRu HEA nanoparticles before and after durability testing, confirming near-equiatomic elemental compositions and negligible metal loss, indicative of excellent structural and compositional stability of the noble-metal HEA catalyst.

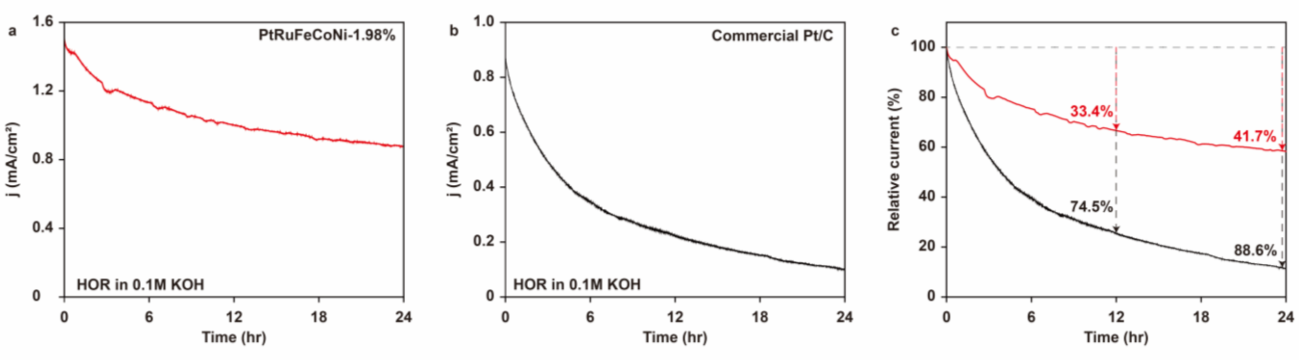

Figure S42. HOR stability comparison between PtRuFeCoNi-1.98% HEA and commercial Pt/C catalysts. Chronoamperometric (i-t) curves of (a) PtRuFeCoNi-1.98% and (b) commercial Pt/C recorded at 0.05 V_{RHE} in 0.1 M KOH for 24 hours. (c) Relative current retention as a function of time for both catalysts. The PtRuFeCoNi HEA catalyst exhibits only 33.4% and 41.7% current decay after 12 and 24 h, respectively, whereas commercial Pt/C suffers from much more severe degradation, with current losses of 74.5% and 88.6% over the same durations. These results clearly demonstrate the superior operational durability of the HEA catalyst for the HOR under alkaline conditions.

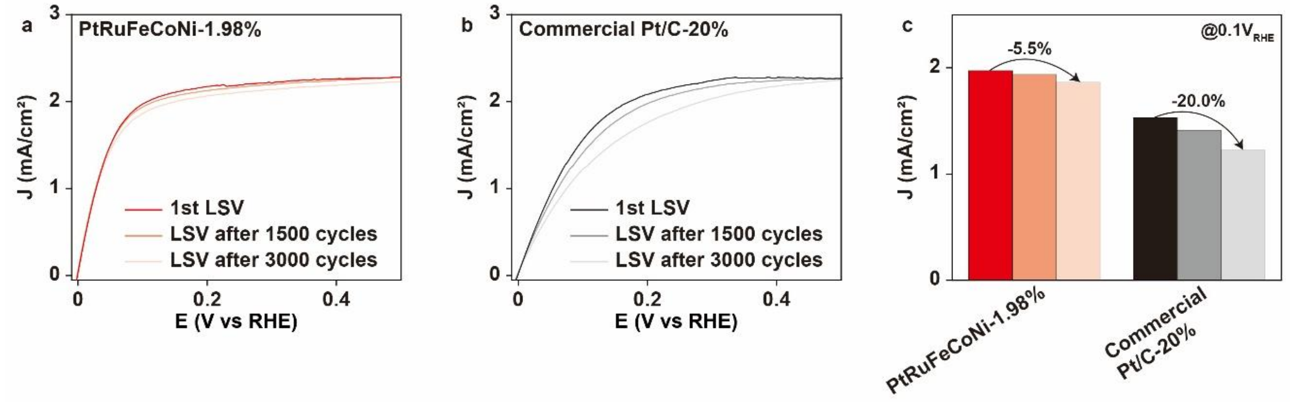

Figure S43. Electrochemical accelerated durability test (ADT) evaluating the alkaline HOR stability of the catalysts during continuous potential cycling. HOR polarization curves of (a) PtRuFeCoNi HEA nanoparticles and (b) commercial Pt/C recorded at the 1st, 1500th, and 3000th cycles. (c) Comparison of the geometric current density retained at 0.1 V_{RHE} during the 3000-cycle durability test. After 3000 potential cycles, PtRuFeCoNi-1.98% shows a current-density decay of 5.5%, whereas commercial Pt/C shows a larger decay of 20.0% under the same testing conditions.

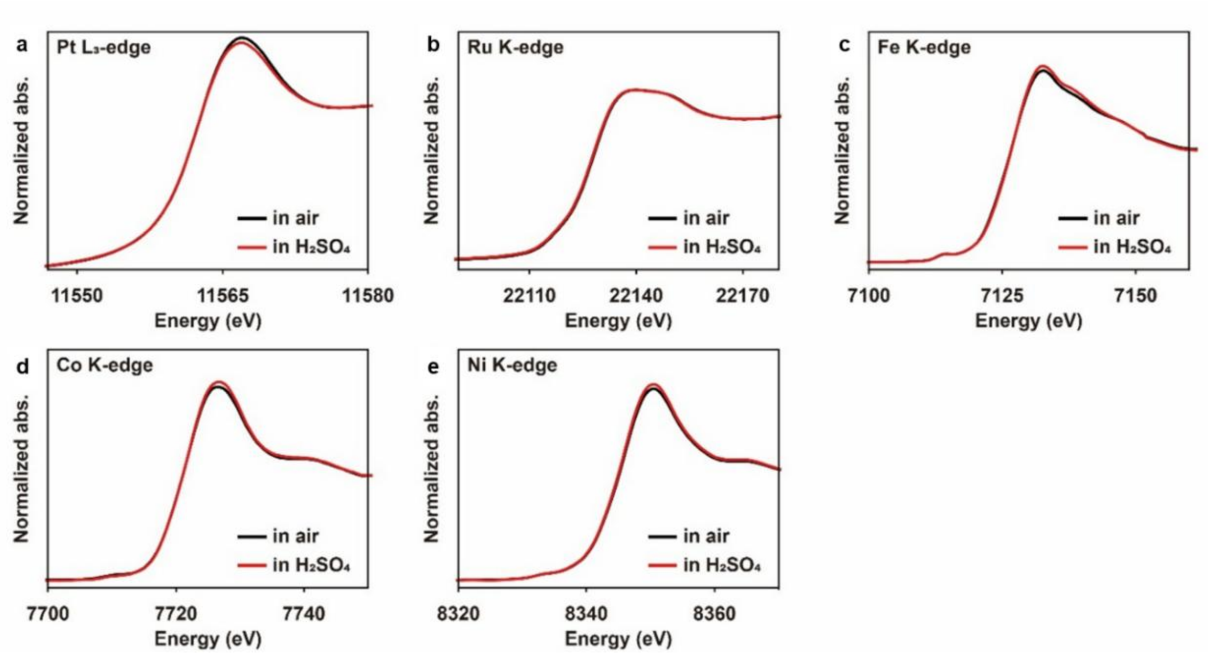

Figure S44. Normalized XANES spectra of PtRuFeCoNi HEA nanoparticles collected in air (black) and in 0.5 M H_2SO_4 electrolyte (red) at the (a) Pt L_3-edge, (b) Ru K-edge, (c) Fe K-edge, (d) Co K-edge, and (e) Ni K-edge. Overall, the edge positions and white-line intensities remain largely comparable under ex situ and in situ conditions, indicating that the average oxidation states of all constituent elements do not undergo significant changes. Minor spectral variations observed in the electrolyte are attributed to surface adsorption/desorption processes and slight surface reconstruction under electrochemical environments, which are typical for ultrasmall multimetallic nanoparticles.

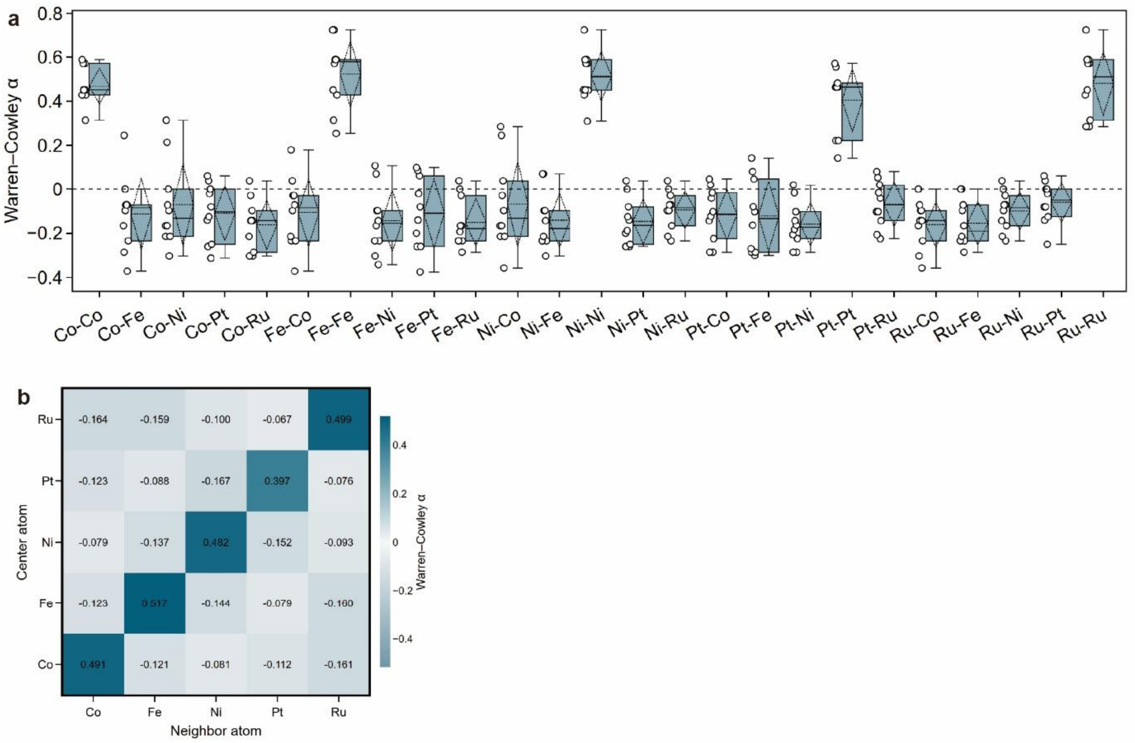

Figure S45. Warren-Cowley short-range order (SRO) parameters derived from ten DFT-generated structural models of PtRuFeCoNi HEA nanoparticles. (a) Averaged Warren–Cowley parameters over all models. (b) Element-pair–resolved Warren–Cowley parameters (25 pairs) from one representative PtRuFeCoNi model. To quantitatively characterize the degree of random mixing, we evaluated SRO using the multicomponent formalism of de Fontaine, which generalizes the Warren-Cowley parameter. For each center element i and neighbor element $j$within the first coordination shell, we computed the pairwise Warren-Cowley SRO parameter as $\alpha_{ij} = 1 - \frac{P_{ij}}{c_j}$, where c_j is the overall atomic fraction of element j, and P_{ij} is the conditional probability of finding j among the nearest neighbors of a center atom i. Under a perfectly random solid solution, $P_{ij} = c_j$ and thus $\alpha_{ij} \approx 0$. In this definition, $\alpha_{ij} < 0$ indicates a preference for unlike-atom coordination (enhanced mixing), whereas $\alpha_{ij} > 0$ indicates avoidance of j around i relative to random. As shown in Figure S39a, the averaged SRO values over all ten models are close to zero and predominantly negative for heteroatomic pairs, indicating a near-random local environment with a mild preference for unlike-atom coordination. Taking Pt as a representative example, Figure S39b shows α_{PtPt} = 0.397, while α_{PtRu} = -0.067, α_{PtFe} = -0.079, α_{PtCo} = -0.112, α_{PtNi} = -0.152. These values indicate that Pt atoms are statistically less likely to have Pt nearest neighbors than expected for a random alloy, and instead preferentially coordinate with the other constituent elements, which is inconsistent with Pt-rich clustering and supports a well-mixed local chemical environment. Importantly, the same trends are reproduced across the full ensemble of ten independently generated and relaxed models, indicating that no specific elemental pairing, ordering motif, or phase separation is artificially introduced by the model construction.

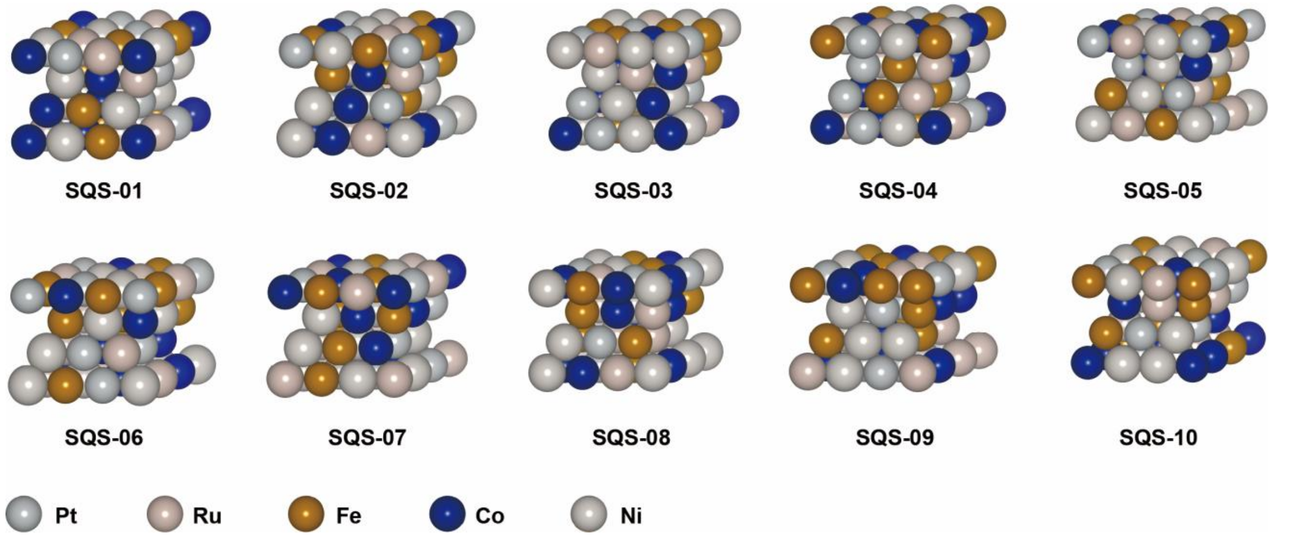

Figure S46. Structural configurations of the PtRuFeCoNi HEA (111) slab models. Ten representative special quasi-random structure (SQS) models (denoted as SQS-01 to SQS-10) were constructed to represent the random atomic arrangement of the quinary HEA system. Each slab consists of multiple atomic layers where the five constituent elements (Pt, Ru, Fe, Co, and Ni, represented by spheres of different colors) are distributed to effectively capture the chemical complexity and lattice distortion of the high-entropy state. These models serve as the basis for the high-throughput sampling of atomic hydrogen adsorption configurations on the (111) surface facets.

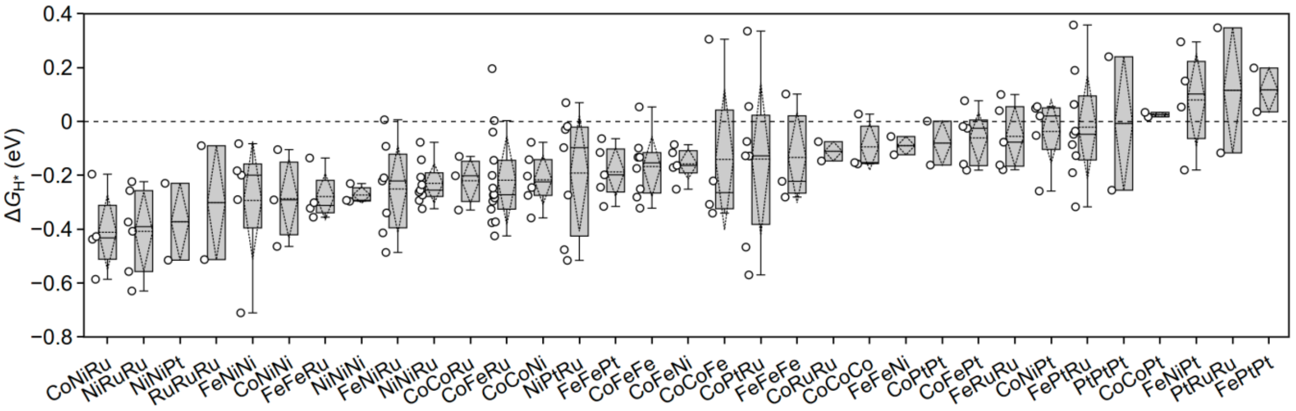

Figure S47. Statistical distribution of hydrogen adsorption free energies (ΔG_{H*}) for various trimeric hollow site configurations on the PtRuFeCoNi HEA surface. The individual data points (circles) represent different hydrogen adsorption configurations sampled across the high-entropy surface, while the box plots illustrate the distribution of ΔG_{H*} values for each specific trimeric ensemble ($M_1M_2M_3$). The horizontal dashed line at 0 eV signifies the ideal thermoneutral adsorption strength according to the Sabatier principle. The wide range of ΔG_{H*} values across distinct hollow site compositions highlights the configurational diversity inherent to the quinary HEA system. Ru-rich containing ensembles such as CoNiRu, NiRuRu, and RuRuRu exhibit more negative ΔG_{H*} values, indicating stronger binding. In contrast, several Pt-containing mixed ensembles, including PtPtRu, CoPtPt, CoNiPt, CoFePt, and FePtRu, cluster closer to $\Delta G_{H*} \approx 0$ eV, suggesting a more balanced adsorption and desorption behavior. Importantly, the feature around +0.4 eV (Figure 9a) arises from weak binding ensembles with significantly positiveΔG_{H*}, most notably FePtPt, and also PtRuRu and FeNiPt, which imply thermodynamically less favorable H-adsorption and thus reduced surface H coverage under reaction conditions.

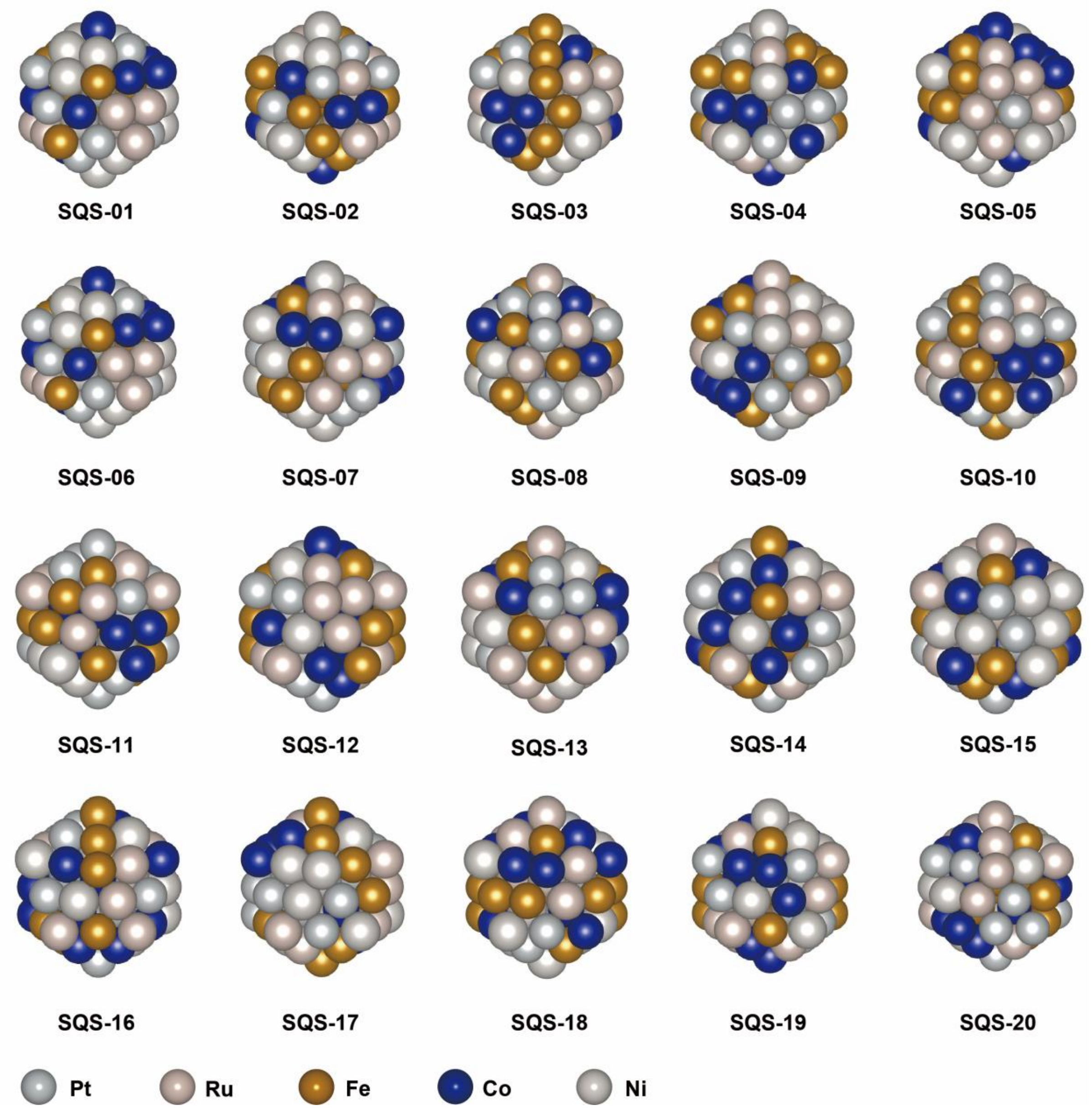

Figure S48. Structural configurations of SQS-01 to SQS-20 55-atom PtRuFeCoNi HEA nanoparticle cluster models used for machine-learning-assisted to evaluateΔG_{H*} at low-coordination sites (vertex and edge).

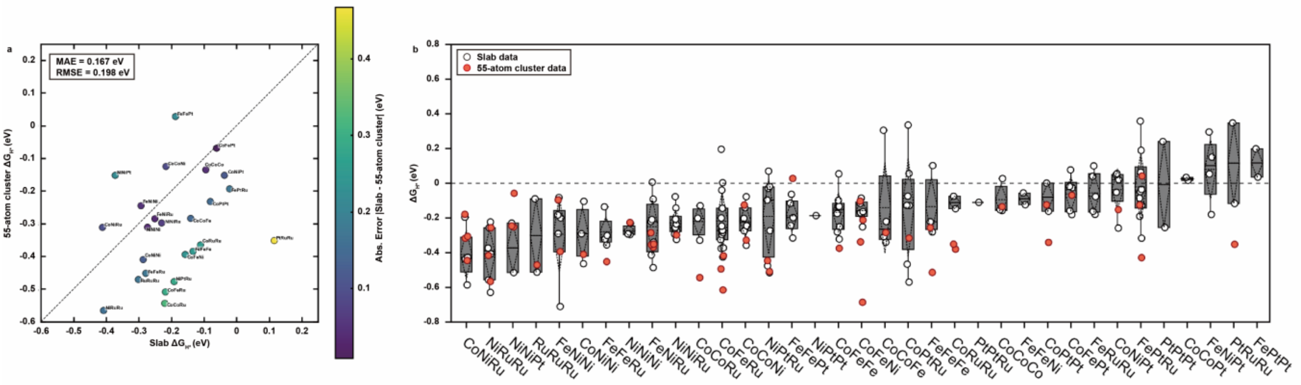

Figure S49. Comparison of Hydrogen Adsorption Energies (ΔG_{H^*}) between Slab and 55-atom Cluster Models. (a) Parity plot of ΔG_{H^*} values comparing the periodic (111) slab model and the 55-atom PtRuFeCoNi HEA nanoparticle cluster. The Mean Absolute Error (MAE) of 0.167 eV and Root Mean Square Error (RMSE) of 0.198 eV indicate a systematic deviation where the cluster model generally exhibits more negative ΔG_{H^*} values (stronger adsorption). This shift is attributed to the presence of under-coordinated surface sites and the strain effects inherent in sub-nanometer clusters. (b) Statistical distribution of ΔG_{H^*} for various trimeric hollow site configurations. The box plots and open white circles represent the ensemble of adsorption energies calculated from the (111) slab model for each specific trimer composition. The red solid circles denote the corresponding values obtained from the 55-atom cluster model. For most configurations, the cluster data points are located near the lower bound or even below the slab's distribution, highlighting that even for identical local trimer compositions, the reduced coordination and electronic environment of the nanoparticle significantly enhance hydrogen adsorption strength.

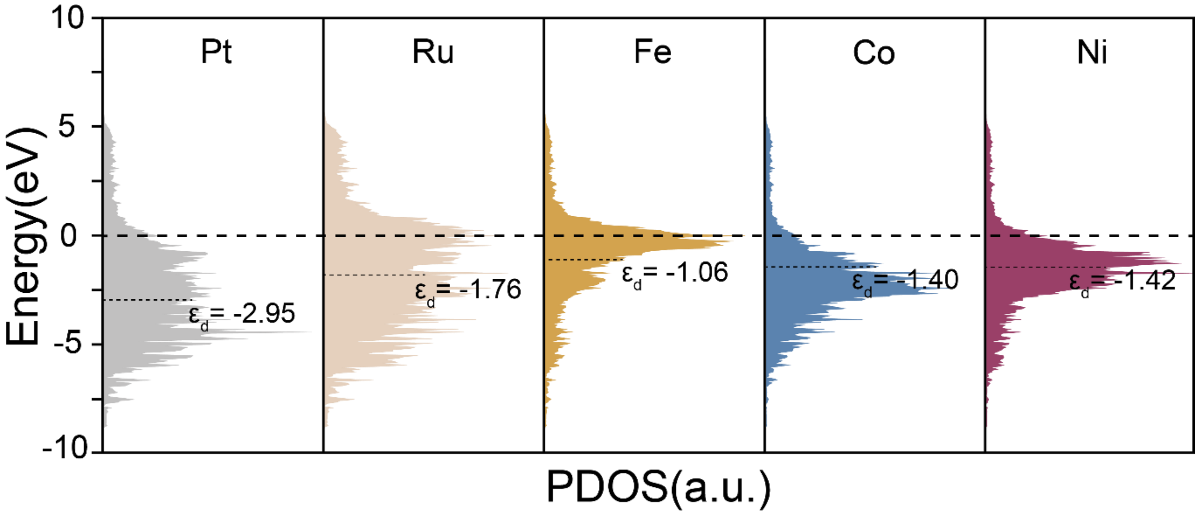

Figure S50. Projected density of states (PDOS) of Pt, Ru, Fe, Co, and Ni in the PtRuFeCoNi HEA nanoparticles. The d-band centers of Pt and Ru are located at lower energies relative to the Fermi level, suggesting moderate hydrogen adsorption strength favorable for the Volmer-Heyrovsky mechanism. In contrast, Fe, Co, and Ni exhibit higher d-band centers, indicating their role in electron donation and local electronic modulation.

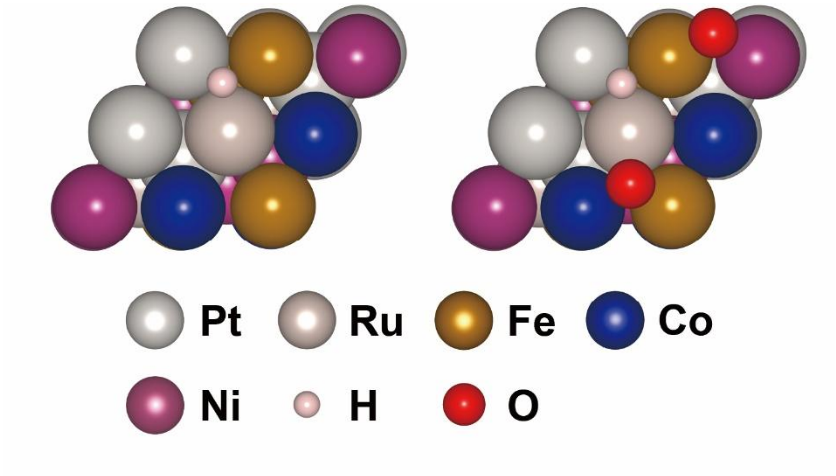

Figure S51. DFT models of PtRuFeCoNi HEA nanoparticles with pristine Fe sites and with locally oxidized Fe-O coordination used for ΔG_{H^*} calculations.

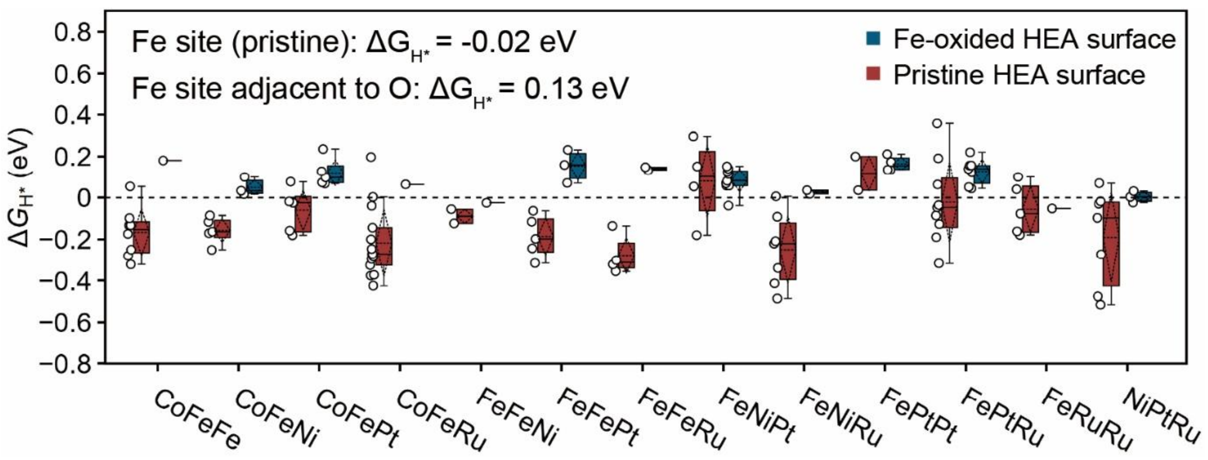

Figure S52. Comparison of hydrogen adsorption free energy (ΔG_{H*}) on PtRuFeCoNi HEA nanoparticles with and without oxygen coordination at Fe sites. For the pristine surface, the calculated ΔG_{H*} on the Fe top site is -0.02 eV. To simulate partial surface oxidation, an O atom was initially placed on the Fe top site; after structural optimization, the O atom migrates to a neighboring hollow site. Hydrogen adsorption was then evaluated on the remaining adjacent Fe-related site, giving a ΔG_{H*} of 0.13 eV.

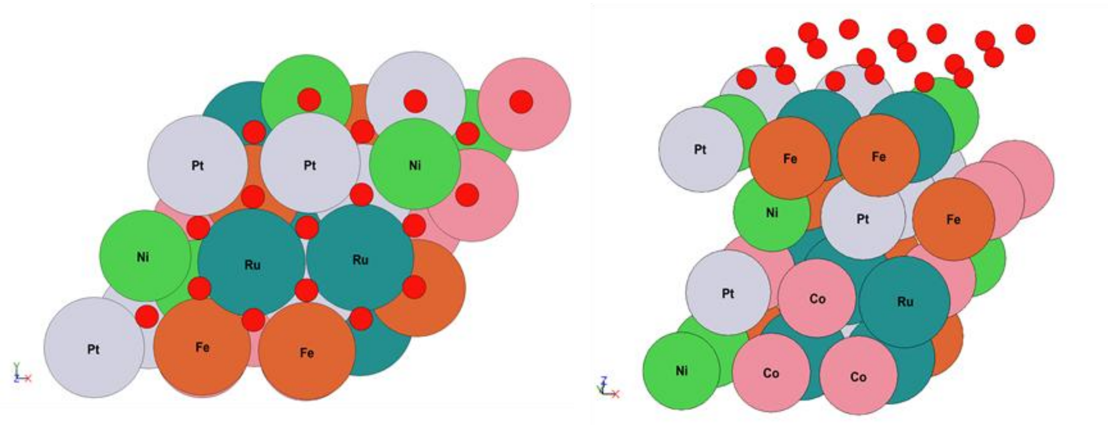

Figure S53. The 18 hollow H-adsorption sites examined on each PtRuFeCoNi (111) slab in this work, with red spheres representing H atoms.

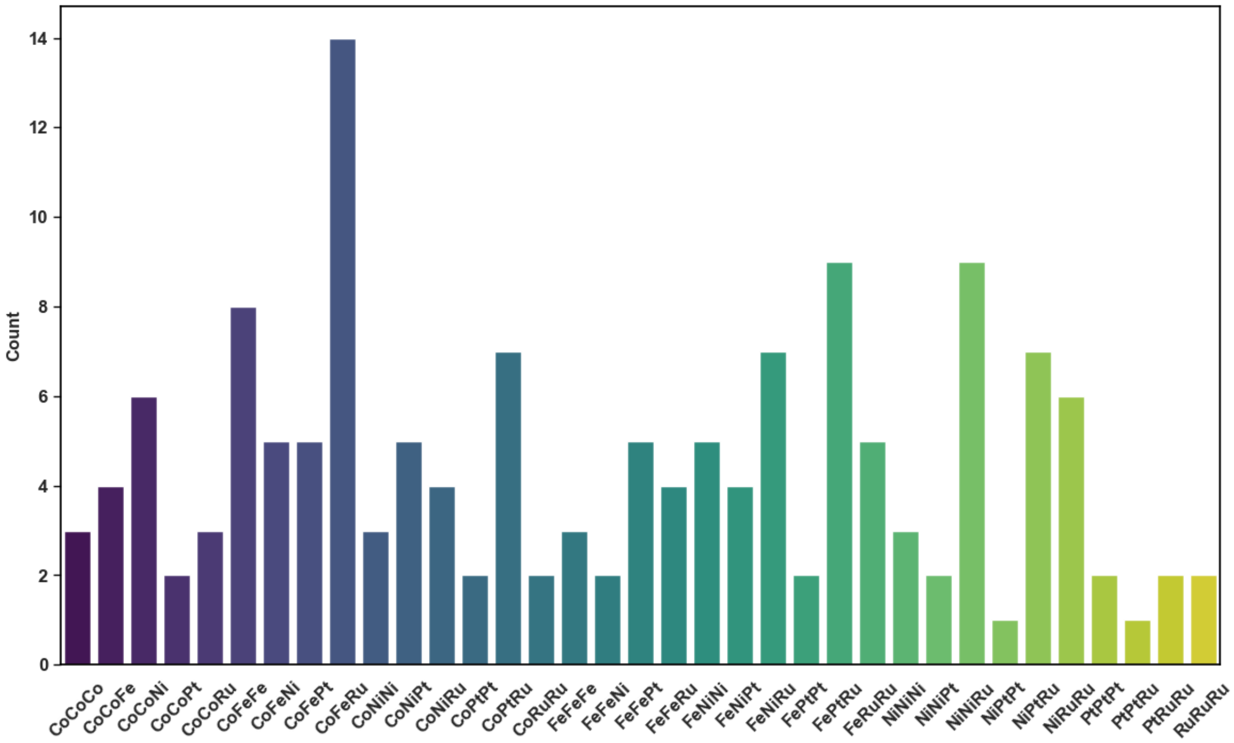

Figure S54. The occurrence frequency of each M1M2M3 hollow site observed in our H-adsorption configuration sampling.

Table S1. Comparison of wet impregnation and pore-volume-matched dry impregnation for synthesizing mesopore-confined HEA nanoparticles. Dry impregnation promotes more effective precursor confinement in CMK-3 mesopores and favors the formation of smaller, better-dispersed nanoparticles.

Comparsion	Wet impregnation	Dry impregnation
Solvent volume	Large	Minimal (pore-volume metched)
Precursor distribution	More likely on external surface and near pore entrances	More effectively confined within mesopores
Particle size	Typically larger particles	Ultrasmall particles
Aggregation risk	Higher	Lower due to spatial confinement

Table S2. Preparation methods, size distributions, and catalytic applications of HEA nanoparticles in recent reports.

**Materials**	**Methods**	**Particle size**	**Geometric shapes**	**Reactions**	**Ref.**
PtFeCoNiCu	Co-reduction and annealing	*6.13 ± 2.69*	Spherical particles	Hydrogen evolution reaction (HER)	*Nat. Commun.* **15**, 359 (2024).
PtCuNiCoMn	Solvothermal synthesis	*6.96 ± 0.22*	Spherical particles	Methanol oxidation reaction (MOR)	*Langmuir* **40**, 2343-2351 (2024).
PtCoFeNiCu	Co-reduction and annealing	*4.1 ± 0.8*	Spherical particles	Oxygen oxidation reaction (ORR)	*J. Am. Chem. Soc.* **146**, 3010-3022 (2024).
PtIrCuNiCr, PtAuPdFeNi	Laser scanning ablation	*11 ± 4.6*	Spherical particles	HER, oxygen evolution reaction (OER)	*Nat. Synth.* **1**, 138-146 (2023).
PtFeCoNiCu	Wet-chemical synthesis	*1.58 ± 0.4*	Spherical particles	Aqueous-phase hydrogenation of maleic anhydride	*ACS Appl. Mater. Interfaces* **15**, 23276-23285 (2023).
RhRuPtPdIr	Sequential atomic layer deposition & heat treatment	*45 ± 10*	Film	HER	*Langmuir* **39**, 3142-3150 (2023).
NiPdPtRhIr	Wet-chemical synthesis	*6.55 ± 1.64*	Spherical particles	None	*ACS Nano* **17**, 5943-5955 (2023).
PtZrNbFeCuTaMoHfBiWZnSnPdNi	Step alloying	*5.03 ± 0.95*	Spherical particles	HER, ORE, MOR	*Adv. Mater.* **35**, 2302499 (2023).
PdNiRuIrRh	Wet-chemical synthesis	*4.72 ± 0.95*	Spherical particles	Hydrogen oxidation reaction (HOR)	*Angew. Chem.* **135**, e202217976 (2023).
NiFeCoCuSnMo	Sparking synthesis	*5*	Spherical particles	Lithium-sulfur redox reaction	*Nat. Nanotechnol.* **18**, 153-159 (2023).
NiPdPtRhIr	Wet-chemical synthesis	*5.61 ± 1.14*	Spherical particles	None	*ACS Nano*, **17**, 5943-5955 (2023).
IrPdPtRhRu	Wet-chemical synthesis	*4.1 ± 1.2*	Spherical particles	HER	*J. Am. Chem. Soc.* **144**, 3365-3369 (2022).
FeCoNiCuPdIrPtAu	Fast-moving bed pyrolysis	*80-300*	Spherical particles	None	*Nat. Commun.* **13**, 2789 (2022).
FeCoNiSnPd	High-temperature shock	*24.81 ± 3.56*	Spherical particles	Ethanol oxidation reaction (EOR)	*Adv. Funct. Mater.* **32**, 2204643 (2022)
PtCoCuRuNiFe	Thermal decomposition reduction	*2-10*	Spherical particles	MOR	*ACS Nano* **16,** 14017-14028 (2022).
PdCuPtNiCo, PdCuPtNiFe	Wet-chemical synthesis and annealing	*11.3 ± 0.7*	Spherical particles	Oxygen reduction reaction (ORR)	*ACS Nano* **16**, 18873-18885 (2022).

IrPdPtRhRu	Continuous-flow reactor synthesis	*1.32 ± 0.41*	Spherical particles	HER	*J. Am. Chem. Soc.* **144**, 11525-11529 (2022).
CoNiCuRuPd	Hydrogen spillover-driven synthesis	*1.9*	Spherical particles	Carbon dioxide reduction reaction (CO_2RR)	*Nat. Commun.***12**, 3884 (2021).
PtRuNiCoFeMo	Wet-chemical synthesis	*1.8 ± 0.3*	Nanowires	HOR	*Nat. Commun.* **12**, 6261 (2021).
RuFeCoNiCu	Wet-chemical synthesis	*15.07 ± 0.89*	Spherical particles	Nitrogen reduction reaction (N_2RR)	*Adv. Funct. Mater.* **31**, 2006939 (2021).
PtPdFeCoNi	Microwave heating	*12.8 ± 5.1*	Spherical particles	None	*ACS Nano* **15**, 14928-14937 (2021).
NiCoFePtRh	Co-reduction and annealing	*1.68*	Spherical particles	HER	*J. Am. Chem. Soc.* **143**, 17117-17127 (2021).
PtNiFeCoCu	Wet-chemical synthesis	*4.1 ± 1.2*	Spherical particles	HER, MOR	*Nat. Commun.* **11**, 5437 (2020).
RuIrFeCoNi	Droplet-to-particle	*218 ± 2*	Hollow spherical particles	ORR, OER	*Adv. Mater.* **32**, 2002853 (2020).
FeCoNiCuPt, FeCoNiPdPt, FeCoNiCuPd	Aerosol synthesis	*92 ± 1.77*	Spherical particles	None	*Langmuir* **36**, 1985-1992 (2020).
AuAgPtPdCu	High-temperature synthesis	*16 ± 10*	Spherical particles	CO_2RR	*ACS Catal.* **10**, 3658-3663 (2020).
RuRhPdOsIrPt	Wet-chemical synthesis	*3.1 ± 0.6*	Spherical particles	EOR	*J. Am. Chem. Soc.* **142**, 13833−13838 (2020).
FeCoPdIrPt	Fast moving bed pyrolysis	*1.5-2.5*	Spherical particles	HER	*Nat. Commun.* **11**, 2016 (2020).
PdIrPtRhRu	Solvothermal synthesis	*3–15*	Spherical particles	None	*Angew. Chem. Int. Ed.* **59**, 21920-21924 (2020).
CoMoFeNiCu	Carbothermal shock	*22*	Spherical particles	NH_3 decompositi on	*Nat. Commun.* **10**, 4011 (2019).
CoCrFeMnNi	ultrashort-pulsed laser ablation	*2.8 ± 0.6*			*RSC Adv.* **9**,18547-18558 (2019)
PtPdCoNiFeAuCuS n	Carbothermal shock	*3.28 ± 0.81*	Spherical particles	NH_3 oxidation	*Science* **359**, 1489-1494 (2018).

Table S3. The standard reduction potentials for different metal precursors.[14]

**Reaction**	**Reduction Potential E (V_{SHE})**
$Fe^{3+} + 3e^{-} \rightarrow Fe$	-0.037
$Co^{2+} + 2e^{-} \rightarrow Co$	-0.280
$Ni^{2+} + 2e^{-} \rightarrow Ni$	-0.257
$Mo^{3+} + 3e^{-} \rightarrow Mo$	-0.200
$Zn^{2+} + 2e^{-} \rightarrow Zn$	-0.762
$Cu^{2+} + 2e^{-} \rightarrow Cu$	0.342
$Rh^{3+} + 3e^{-} \rightarrow Rh$	0.758
$Ru^{3+} + e^{-} \rightarrow Ru^{2+}$	0.249
$Ru^{2+} + 2e^{-} \rightarrow Ru$	0.455
$PdCl_4^{2+} + 2e^{-} \rightarrow Pd + 4Cl^{-}$	0.591
$PtCl_6^{2+} + 2e^{-} \rightarrow PtCl^{2-} + 2Cl^{-}$	0.680
$PtCl^{2-} + 2e^{-} \rightarrow Pt + 4Cl^{-}$	0.755

Table S4. Atomic ratio of N 1s from N-doped CMK-3 obtained by XPS analysis (Figure S3).

**N-doped CMK-3**	**C**	**O**	**N**
Atomic ratio (at.%)	94.2	3.7	2.1

**N 1s**	**Graphitic N**	**Pyrrolic N**	**Pyridinic N**	**Oxidized N**
Atomic ratio (at.%)	59.8	28.5	13.4	8.3

Table S5. Lattice constants of FCC-phase Pt, Ru, Fe, Co, and Ni. The theoretical lattice constant of PtRuFeCoNi was calculated from ICP-OES result (Pt/Ru/Fe/Co/Ni = 17.8/15.6/24.9/20.4/21.3 at.%) according to Vegard's law. The experimentally derived value (4.0 Å) is larger than the theoretical prediction, which could arise from the severe atomic size mismatch inherent to HEAs, the lattice expansion can also be attributed to the small particle size, or partial surface oxidation of easily oxidized elements.

Element	Lattice constant (Å)
Pt	3.92
Ru	3.82
Fe	3.66
Co	3.51
Ni	3.52
PtRuFeCoNi	3.67

Table S6. XPS sensitivity factor (SF) for the elements in the PtRuFeCoNi HEA nanoparticles.

Chemical state	Sensitivity factor (SF)
Pt 4f	6.080
Ru 3d	4.529
Ru 3p	2.236
Fe 2p	2.946
Co 2p	3.529
Ni 2p	3.702

Table S7. The binding energies of the elements for the PtRuFeCoNi nanoparticles using the XPS analysis.

**Chemical state**	**Pt $4f_{7/2}$**	**Pt^{2+} $4f_{7/2}$**
Table lookup value (eV)	71.0	72.4
PtRuFeCoNi	71.7	73.0

**Chemical state**	**Ru $3p_{3/2}$**	**Ru^{4+} $3p_{3/2}$**
Table lookup value (eV)	461.2	465.4
PtRuFeCoNi	462.8	467.7

**Chemical state**	**Fe $2p_{3/2}$**	**Fe^{2+} $2p_{3/2}$**	**Fe^{3+} $2p_{3/2}$**
Table lookup value (eV)	706.7	709.6	710.8
PtRuFeCoNi	706.8	710.7	712.7

**Chemical state**	**Co $2p_{3/2}$**	**Co^{2+} $2p_{3/2}$**
Table lookup value (eV)	778.2	779.7
PtRuFeCoNi	778.6	781.9

**Chemical state**	**Ni $2p_{3/2}$**	**Ni^{2+} $2p_{3/2}$**
Table lookup value (eV)	852.6	853.7
PtRuFeCoNi	854.0	856.4

Table S8. Structural parameters obtained from EXAFS fitting analyses for the PtRuFeCoNi HEA nanoparticles, as well as their corresponding metallic foils. In the typical procedure, the Artemis software is employed for fitting EXAFS, with a focus on k^2-weighted experimental signals. The fitting parameters encompass k-ranges of 3 to 10 Å^{-1} for five elements within PtRuFeCoNi HEA nanoparticles and the foil references, respectively. Additionally, R-windows range from 1.1 to 3 Å for PtRuFeCoNi_Pt, 1 to 3.3 Å for Pt foil, 1 to 3 Å for PtRuFeCoNi_Ru, 1.1 to 3 Å for Ru foil, 1 to 3 Å for PtRuFeCoNi_Fe, 1 to 3 Å for Fe foil, 1 to 3 Å for PtRuFeCoNi_Co, 1 to 3 Å for Co foil, 1.2 to 3 Å for PtRuFeCoNi_Ni, and 1 to 3 Å for Ni foil, respectively.

**Sample**	**Bond**	**[a]CN**	**[b]R (Å)**	**[c]σ^2**	**[d]S_0^2**	**[e]ΔE_0 (eV)**	**[f]R factor**
Pt foil	Pt-Pt	12	2.76 (fcc)	0.003	0.752	7.469	0.0117
PtRuFeCoNi_Pt	Pt-Pt	2.5 ± 1.9	2.78 ± 0.02	0.003			
	Pt-Ru	1.6 ± 0.9	2.75 ± 0.04	0.003			
	Pt-3d	1.7 ± 1.0	2.62 ± 0.06	0.009			
	Pt-O	0.7 ± 0.3	2.03 ± 0.13	0.004			
Ru foil	Ru-Ru	12	2.67	0.003	0.708	0.246	0.0345
PtRuFeCoNi_Ru	Ru-Pt	1.4 ± 0.8	2.88 ± 0.08	0.008			
	Ru-Ru	2.8 ± 0.8	2.73 ± 0.02	0.010			
	Ru-3d	1.5 ± 0.8	2.58 ± 0.04	0.014			
	Ru-O	3.8 ± 0.3	1.99 ± 0.01	0.003			
Fe foil	Fe-Fe	8	2.45 (bcc)	0.004	0.696	4.669	0.0237
		6	2.84 (bcc)	0.004			
PtRuFeCoNi_Fe	Fe-Pt	1.7 ± 1.2	2.46 ± 0.07	0.004			
	Fe-Ru	1.3 ± 1.0	2.38 ± 0.06	0.005			
	Fe-O-3d	2.5 ± 2.0	3.04 ± 0.07	0.007			
	Fe-O	4.5 ± 1.2	2.01 ± 0.02	0.003			
Co foil	Co-Co	12	2.48 (fcc)	0.006	0.725	6.258	0.0175
PtRuFeCoNi_Co	Co-Pt	2.7 ± 1.5	2.57 ± 0.04	0.003			
	Co-Ru	0.9 ± 0.4	2.51 ± 0.06	0.003			
	Co-O-3d	2.2 ± 1.4	3.08 ± 0.04	0.003			
	Co-O	4.2 ± 1.0	2.14 ± 0.02	0.003			
Ni foil	Ni-Ni	12	2.47 (fcc)	0.007	0.902	5.503	0.0329
PtRuFeCoNi_Ni	Ni-Pt	2.0 ± 1.4	2.56 ± 0.07	0.004			
	Ni-Ru	2.0 ± 1.1	2.43 ± 0.04	0.008			
	Ni-O-3d	2.1 ± 1.5	3.04 ± 0.09	0.004			
	Ni-O	3.7 ± 1.1	2.11 ± 0.03	0.003			

[a]CN = coordination number.

[b]R = radial distance.

[c]σ^2 = mean square relative displacements

[d]$S0^2$: fixed amplitude reduction factor based on first-shell fitting of metallic foils.

[e] ΔE_0 : fitted energy shift parameter.

[f] *R factor* = $\sum(\text{data-fit})^2/\sum \text{data}^2$)

Table S9. EXAFS fitting parameters at the Pt L_3-edge obtained using a simplified single-path Pt-Pt model for Pt foil and PtRuFeCoNi HEA nanoparticles. This control fitting shows that a Pt-Pt-only model provides an inadequate description of the HEA sample compared with the multipath model. Specifically, the multipath fit gives a low R-factor of 0.0117 (Table S8), whereas the simplified single-path fit yields a much poorer agreement, with an R-factor of 0.0737 (Table S9). These results indicate that a single-path model cannot adequately capture the chemically heterogeneous first-shell environment of the multimetallic HEA, and that incorporation of multiple Pt-Me scattering paths (Me = Ru, Fe, Co, Ni) is necessary to accurately describe the local coordination around Pt. Although the inclusion of additional scattering paths inevitably increases the uncertainty of the fitted parameters, all extracted values remain within physically reasonable ranges, and the substantially improved goodness-of-fit supports the robustness and reliability of the multipath fitting approach.

Sample	Bond	^{a}CN	^{b}R (Å)	$^c\sigma^2$	$^dS_0^2$	$^e\Delta E_0$ (eV)	^{f}R factor
Pt foil	Pt-Pt	12	2.76 (fcc)	0.003	0.752	7.469	0.0737
PtRuFeCoNi_Pt	Pt-Pt	7.3 ± 3.6	2.72 ± 0.03	0.009 ± 0.005			
	Pt-O	1.9 ± 1.9	2.05 ± 0.10	0.015 ± 0.020			

Table S10. The melting points, boiling points, reduction temperatures, and decomposition temperatures of various metal precursors.

**Metal precursors**	**Melting point (°C)**	**Boiling point (°C)**	**Reduction temperature (°C)**	**Decomposition temperature (°C)**
$FeCl_3 \cdot 6H_2O$	306	315	400-600	500-800
$CoCl_2 \cdot 6H_2O$	735	1049	300-505	600-900
$NiCl_2 \cdot 6H_2O$	1001	N/A	300-600	350-740
$MoCl_5$	194	268	350-480	500-800
ZnC_2	290	732	250-450	400-500
$CuCl_2$	630	993	500-700	400-1000
$RhCl_3 \cdot xH_2O$	450	717	100-130	500-900
$RuCl_3 \cdot xH_2O$	>500 (decomposes)	N/A	120-160	400-800
Na_2PdCl_4	N/A	N/A	153	600-740
$H_2PtCl_6 \cdot 6H_2O$	60	Decomposes	122	300-550

Table S11. A summary of the electrochemical protocol used for the electrocatalytic HER and HOR measurements, outlining the key conditions and steps involved in the experiments.

HER in 0.5 M H_2SO_4			
**Step**	**Electrochemical Technique**		**Parameters**
Electrochemical cleaning	Cyclic voltammograms (CV)	Gas purge (Flow rate)	N_2 (100 mL min^{-1})
		Potential limits	0.05 – 1.1 V vs RHE
		Scan rate	500 mV s^{-1}
		Number of cycles	150
ECSA determination (H_{upd})	CV	Gas purge (Flow rate)	N_2 (100 mL min^{-1})
		Potential limits	0.05 – 1.1 V vs RHE
		Scan rate	50 mV s^{-1}
		Number of cycles	3
Oxide reduction	Chronoamperometry (CA)	Gas purge (Flow rate)	N_2 (100mL min^{-1})
		Potential	-0.03 V vs RHE
		Time	120 min
Polarization curve (HER)	Linear sweep voltammetry (LSV)	Gas purge (Flow rate)	N_2 (100 mL min^{-1})
		Potential limits	-0.2 – 0.1 V vs RHE
		Scan rate	0.001 V s^{-1}
		Step	-0.00244 V
ECSA determination (CO-stripping)	CA	Gas purge (Flow rate)	CO (100 mL min^{-1})
		Potential	0.1 V vs RHE
		Time	10 min
		Gas purge (Flow rate)	N_2 (100 mL min^{-1})
		Potential	0.1 V vs RHE
		Time	20 min
	CV	Gas purge (Flow rate)	N_2 (0 mL min^{-1})
		Potential limits	0.05 – 1.1 V vs RHE
		Scan rate	50 mV s^{-1}
		Number of cycles	2
**HOR in 0.1 M KOH**			
**Step**	**Electrochemical Technique**		**Parameters**
Electrochemical cleaning	Cyclic voltammograms (CV)	Gas purge (Flow rate)	N_2 (100 mL min^{-1})
		Potential limits	0.05 – 1.1 V vs RHE
		Scan rate	500 mV s^{-1}
		Number of cycles	40
Polarization curve (HOR)	Linear sweep voltammetry (LSV)	Gas purge (Flow rate)	H_2 (100 mL min^{-1})
		Potential limits	-0.1 – 0.7 V vs RHE
		Scan rate	0.005 V s^{-1}
		Step	0.00244 V

Table S12. The atomic ratios of the obtained HEA nanoparticles, mono-, bi-, and tri-metallic nanoparticles on the N-doped CMK-3, PtRuFeCoNi HEA nanoparticles on XC-72R, and commercial Pt/C.

	Composition (at%)							Loading amount (wt%)	
**Sample name**	**Fe**	**Co**	**Ni**	**Ru**	**Pt**	**Rh**	**Pd**	**Noble metal**	**All**
PtRuFeCoNi-1.98%	24.9	20.4	21.3	15.6	17.8	0	0	1.98	3.49
PtPdFeCoNi-1.68%	26.7	19.3	20.1	0	16.1	0	17.8	1.68	2.99
PdRhFeCoNi-1.12%	26.4	19.9	22.1	0	0	16.1	15.5	1.12	2.46
PtRhFeCoNi-1.99%	25.8	20.8	21.2	0	16.0	16.2	0	1.99	3.62
RuPdFeCoNi-1.23%	28.0	20.3	20.9	15.7	0	0	15.0	1.23	2.75
RuRhFeCoNi-1.12%	24.0	18.3	22.3	15.4	0	20.0	0	1.12	2.43
PtRuFeCoNi-3.09%	22.2	16.2	16.9	26.9	17.8	0	0	3.09	4.68
PtRuFeCoNi-4.28%	17.1	18.2	17.5	25.5	21.8	0	0	4.28	6.19
PtRuFeCoNi-5.71%	18.6	21.1	22.6	14.6	23.2	0	0	5.71	9.16
FeCoNi	33.9	30.6	35.5	0	0	0	0	0	2.16
RuPt-3%	0	0	0	31.0	69.0	0	0	3.00	3.00
Pt-4.8%	0	0	0	0	100	0	0	4.80	4.80
PtRuFeCoNi on XC-72R-2.7%	22.5	21.0	23.3	14.9	18.3	0	0	2.70	4.76
Commercial Pt/C-20%	0	0	0	0	100	0	0	20.00	20.00

Table S13. The HER electrochemical measurements results of the catalysts measured in this work.

Catalyst	Electrolyte	η_{10} (mV)	CO stripping (cm^2)	Mass activity @50mV (A /mg $_{noble\ metal}$)	Compare with commercial Pt/C
PtRuFeCoNi-1.98%	0.5 M H_2SO	47.7	0.064	11.8	4.9
PtPdFeCoNi-1.68%	0.5 M H_2SO	103.6	0.047	3.6	1.5
PdRhFeCoNi-1.12%	0.5 M H_2SO	173.4	0.097	1.2	0.5
PtRhFeCoNi-1.99%	0.5 M H_2SO	89.7	0.383	4.1	1.7
RuPdFeCoNi-1.23%	0.5 M H_2SO	206.3	0.077	0.3	0.1
RuRhFeCoNi-1.12%	0.5 M H_2SO	167.2	0.413	1.4	0.6
PtRuFeCoNi-3.09%	0.5 M H_2SO_4	31.5	0.074	15.6	6.4
PtRuFeCoNi-4.28%	0.5 M H_2SO	27.5	0.119	14.2	5.9
PtRuFeCoNi-5.71%	0.5 M H_2SO	36.9	0.205	6.6	2.8
PtRu-3.00%	0.5 M H_2SO	34.2	0.313	6.1	2.5
Pt-4.80%	0.5 M H_2SO	119.2	0.340	0.6	0.3
PtRuFeCoNi on XC-72R-2.70%	0.5 M H_2SO	67.3	0.067	3.6	1.5
Commercial Pt/C-20%	0.5 M H_2SO	28.3	0.427	2.4	1

Table S14. The comparison of different catalysts in terms of overpotential and mass activity during the electrochemical HER test in 0.5 M H_2SO_4 solution.

Catalyst	Electrolyte	Mass activity (A /mg $_{noble\ metal}$)	Reference
1-nm PtRuFeCoNi-3.09%	0.5 M H_2SO_4	2.6 @20mV; 5.9 @30mV; 15.6@ 50 mV; 25.6@ 70 mV; 41.3 @100mV	This work
3%PtSn/ATO	0.5 M H_2SO_4	8.37 @50mV	*Small* **20**, 2307135 (2024).
$PtNi_{SA-NPs}$/NDCF	0.5 M H_2SO_4	7.7 @50mV	*Sustain. Mater. Technol.* **41**, e01068 (2024).
CoRuP	0.5 M H_2SO_4	1.03 @50mV	*Carbon Energy* **6**, e556 (2024).
Ru_1Pt_2@rGO	0.5 M H_2SO_4	8.39 @50mV	*Adv. Funct. Mater.* 2411081 (2024).
Ru@1T-MoS_2-MXene	0.5 M H_2SO_4	0.79 @100mV	*Adv. Funct. Mater.* **33**, 2212514 (2023).
Pt_3Fe/NMCS	0.5 M H_2SO_4	1.94 @30mV	*Adv. Mater.* **35**, 2303030 (2023).
PtNiNb	0.5 M H_2SO_4	7.2 @100mV	*Nat. Commun.* **14**, 5389 (2023).
Pt@Mn-SAs/N-C	0.5 M H_2SO_4	1.5 @30mV	*ACS Catal.* **13**, 4012-4020 (2023).
PtCu/WO_3@CF	0.5 M H_2SO_4	10.86 @100mV	*Adv. Funct. Mater.* **32**, 2112207 (2022).
Pt_{doped}@WC_x	0.5 M H_2SO_4	9.87 @70mV	*Adv. Mater.* **34**, 2206368 (2022).

Pt/MXene	0.5 M H_2SO_4	1.85 @50mV	*Adv. Funct. Mater.* **32**, 2110910 (2022).
Ru/Mo_2C@NC	0.5 M H_2SO_4	0.53 @50mV	*J. Mater. Chem. A* **9**, 20518-20529 (2021).
V_o-rich Pt/TiO_2	0.5 M H_2SO_4	45.28 @100mV	*Angew. Chem.* **133**, 16758-16763 (2021).
Pt_1/NMHCS	0.5 M H_2SO_4	2.07 @50mV	*Adv. Mater.* **33**, 2008599 (2021).
PtW_6O_{24}	0.5 M H_2SO_4	8.12 @70mV	*Nat. Commun.* **11**, 490 (2020).
PtW NPs/C	0.5 M H_2SO_4	0.57 @20mV	*J. Am. Chem. Soc.* **142**, 17250-17254 (2020).
Pt SASs/AG	0.5 M H_2SO_4	22.4 @50mV	*Energy Environ. Sci.* **12**, 1000-1007 (2019).
Pt SA-WO_3	0.5 M H_2SO_4	12.8 @50mV	*Angew. Chem.* **131**, 16184-16188 (2019).

References

1. Yuan, X.; Mayanovic, R. A. An empirical study on Raman peak fitting and its application to Raman quantitative research. *Appl. Spectrosc.* **2017**, *71* (10), 2325-2338.
2. Caumon, M. C. Determination of methane content in NaCl-H_2O fluid inclusions by Raman spectroscopy. Calibration and application to the external part of the Central Alps (Switzerland) *Chem. Geol.* **2014**, *378*, 52-61.
3. Sadezky, A.; Muckenhuber, H.; Grothe, H.; Niessner, R.; Pöschl, U. Raman microspectroscopy of soot and related carbonaceous materials: Spectral analysis and structural information. *Carbon* **2005**, *43* (8), 1731-1742.
4. Henry, D. G.; Jarvis, I.; Gillmore, G.; Stephenson, M. Raman spectroscopy as a tool to determine the thermal maturity of organic matter: Application to sedimentary, metamorphic and structural geology. *Earth Sci. Rev.* **2019**, *198*, 102936.
5. Gasteiger, H. A.; Kocha, S. S.; Sompalli, B.; Wagner, F. T. Activity benchmarks and requirements for Pt, Pt-alloy, and non-Pt oxygen reduction catalysts for PEMFCs. *Appl. Catal. B* **2005**, *56* (1-2), 9-35.
6. Maillard, F.; Schreier, S.; Hanzlik, M.; Savinova, E. R.; Weinkauf, S.; Stimming, U. Influence of particle agglomeration on the catalytic activity of carbon-supported Pt nanoparticles in CO monolayer oxidation. *Phys. Chem. Chem. Phys.* **2005**, *7* (2), 385-393.
7. Brett, D. J. L.; Atkins, S.; Brandon, N. P.; Vesovic, V.; Vasileiadis, N.; Kucernak, A. R. Investigation of reactant transport within a polymer electrolyte fuel cell using localised CO stripping voltammetry and adsorption transients. *J. Power Sources* **2004**, *133* (2), 205-213.
8. Glasscott, M. W. Classifying and benchmarking high-entropy alloys and associated materials for electrocatalysis: A brief review of best practices. *Curr. Opin. Electrochem.* **2022**, *34*, 100976.
9. Löffler, T.; Ludwig, A.; Rossmeisl, J.; Schumann, W. What makes high-entropy alloys exceptional electrocatalysts? *Angew. Chem. Int. Ed.* **2021**, *60* (52), 26894-26903.
10. Jiao, W., Ren, Z., Cui, Z., Ma, C., Chen, G., Lu, R., Gan, T., Wang, Z., Xiong, Y., Han, Y. All-round enhancement induced by oxophilic single Ru and W atoms for alkaline hydrogen oxidation of tiny Pt nanoparticles. *Nat. Commun.* **2025**, *16* (1), 833.
11. Li, J., Ghoshal, S., Bates, M. K., Miller, T. E., Davies, V., Stavitski, E., Attenkofer, K., Mukerjee, S., Ma, Z., Jia, Q. Experimental proof of the bifunctional mechanism for the hydrogen oxidation in alkaline media. *Angew. Chem. Int. Ed.* **2017**, *56* (49), 15594-15598.
12. St. John, S., Atkinson III, R. W., Unocic, R. R., Zawodzinski Jr, T. A., Papandrew, A. B. Ruthenium-alloy electrocatalysts with tunable hydrogen oxidation kinetics in alkaline electrolyte. *J. Phys. Chem. C* **2015**, *119* (24), 13481-13487.
13. Ciapina, E. G., Santos, S. F., Gonzalez, E. R. Electrochemical CO stripping on nanosized Pt surfaces in acid media: A review on the issue of peak multiplicity. *J. Electroanal. Chem.* **2018**, *815*, 47-60.

14. Vanysek, P. *CRC Handbook of Chemistry and Physics* (CRC Press: Boca Raton, **2002**).
